# A systematic review of marine macroalgal degradation: Toward a better understanding of macroalgal carbon sequestration potential

**DOI:** 10.1111/jpy.70031

**Published:** 2025-05-27

**Authors:** Jessica R. Kennedy, Caitlin O. Blain

**Affiliations:** ^1^ Leigh Marine Laboratory Institute of Marine Science, University of Auckland Leigh New Zealand; ^2^ Coastal People: Southern Skies Centre of Research Excellence University of Otago Dunedin New Zealand

**Keywords:** blue carbon, carbon sequestration, decomposition, degradation, detritus, dissolved organic carbon, macroalgae, particulate organic matter, seaweed, temperature

## Abstract

Although macroalgae are gaining recognition for their potential role in marine carbon sequestration, critical knowledge gaps related to the fate of macroalgal carbon limit our capacity to quantify rates of macroalgal carbon sequestration. Understanding the degradation dynamics of macroalgal‐derived biomaterials—including tissue/wrack, particulate organic matter/carbon (POM/POC), and dissolved organic carbon (DOC)—as well as the environmental drivers of decomposition are critical for assessing the longevity of macroalgal carbon and the potential storage capacity of macroalgae. Thus, a systematic literature review of macroalgal degradation studies was conducted to compile data, estimate the relative recalcitrance (i.e., relative stability) of macroalgal biomaterials, and elucidate key drivers of macroalgal decomposition dynamics. We found that macroalgal decay trajectories are highly variable and not always best described by the often‐cited exponential decay models. Our analysis demonstrated that temperature was a notable driver of decomposition, with higher temperatures eliciting faster rates of decomposition. Furthermore, we found that brown algae had significantly higher proportions of recalcitrant biomaterials when compared to red algae. The impact of other factors, including biomaterial type, degradation environment, and tissue carbon and nitrogen content on macroalgal degradation, is variable across contexts, warranting further study. These results help to provide a foundation from which to plan and assess future studies on macroalgal degradation, which will improve our understanding of how macroalgae contribute to marine carbon cycles, trophic subsidies, and, potentially, marine carbon sequestration.

**Abbreviations**
AIC: Akaike information criterion
C:N: carbon to nitrogen ratio
CSP: carbon sequestration capacity
DOC: dissolved organic carbon
DOM: dissolved organic matter
eDNA: environmental DNA
GLMM: generalized linear mixed effects model
LMM: linear mixed effects model
NPP: net primary productivity
POC: particulate organic carbon
POM: particulate organic matter
PRISMA: preferred reporting items for systematic reviews and meta‐analyses
RDOC: recalcitrant dissolved organic carbon

## INTRODUCTION

One of our most valuable assets in the fight against climate change is ecosystems that naturally sequester carbon (i.e., remove carbon from the atmosphere and store that carbon for 100 years or more; Lal, 2007). The ocean, which absorbs up to one third of anthropogenic CO_2_ emissions annually, has recently become widely recognized for its substantial role in global carbon cycles (Gruber et al., 2019; Intergovernmental Panel on Climate Change, 2013). Vegetated coastal ecosystems, including seagrass meadows, tidal marshes, and mangrove forests, are carbon sequestration hotspots and have, therefore, been deemed blue carbon ecosystems (Lovelock & Duarte, 2019; Macreadie et al., 2021). Yet, despite being highly productive ecosystems with expansive global distributions (Duarte et al., 2022), macroalgal (i.e., seaweed) forests are often not considered as blue carbon ecosystems due to a current lack of understanding surrounding the ultimate fate of macroalgal‐derived carbon (Dolliver & O'Connor, 2022a; Fujita et al., 2023; Gallagher et al., 2022; Macreadie et al., 2019; Pessarrodona et al., 2023). Most types of recognized blue carbon ecosystems occur within soft sediment habitats (including seagrasses, tidal marshes, and mangroves), which are conducive to the rapid burial of organic matter (which is the primary mode through which carbon sequestration in these ecosystems occurs; Atwood et al., 2020; Mcleod et al., 2011). Macroalgae, however, commonly grow on rocky reefs where the burial of detritus does not happen readily, making assessments of the ultimate fate of macroalgal carbon a much more complex endeavor (Hurd et al., 2022; Krause‐Jensen et al., 2018).

Macroalgae produce and release large amounts of organic matter to the surrounding environment via erosion, breakage, exudation, or total thalli dislodgement (de Bettignies et al., 2013; Duarte et al., 2022; Pessarrodona et al., 2018). Kelp forests, for instance, release more carbon via detritus/litterfall than most other vegetated ecosystems, at a rate comparable to that of tidal marshes (Pessarrodona et al., 2018). Macroalgae release carbon in many different forms, ranging from an entire algal thallus, tissue fragments, particulate organic matter (POM, which includes particulate organic carbon, POC), and dissolved organic matter (DOM, which includes dissolved organic carbon, DOC). Particulate organic matter is typically defined operationally as suspended organic matter collected on a filter (typically 0.2 or 0.7 μm pore size), while dissolved organic matter passes through a filter (again usually 0.2 or 0.7 μm pore size; see Glossary; Hansell, 2001; Kharbush et al., 2020; Lee et al., 2004; Verdugo et al., 2004). On average, 30% of macroalgal net primary productivity (NPP) is released into the water column as DOC, and 60% of macroalgal NPP is eventually released as tissues and/or POM (Pessarrodona et al., 2023), although these rates vary substantially with species, environment, and season. This continual supply of organic matter means that macroalgae are recognized as “carbon donors,” contributing to allochthonous carbon subsidies to other ecosystems or areas (such as seagrass meadows, mangrove forests, coastal/intertidal areas, the open ocean, or the deep sea; Cartraud et al., 2021; Filbee‐Dexter et al., 2018; Hill et al., 2015; Kennedy et al., 2010; Krumhansl & Scheibling, 2012b; Olson et al., 2019).

After being released, macroalgal‐derived carbon can be sequestered in the ocean through three established pathways. First, macroalgal biomaterials may be exported to and buried in neighboring soft sediment habitats, enabling the stable storage of the carbon contained within buried macroalgal biomaterials (Braeckman et al., 2019; Erlania et al., 2023; Moreda et al., 2024; Ørberg et al., 2023; Queirós et al., 2019; Wang et al., 2018). However, although it is commonly assumed that the degradation of marine detritus slows almost to zero in anoxic sediments, there is evidence that degradation continues after macroalgal biomaterials are incorporated into marine sediments (Braeckman et al., 2019; Haram et al., 2020; Rossi, 2006). Also, if the sediments are disturbed, any stored carbon that is released becomes susceptible to reintroduction to the atmosphere (Lovelock, Atwood, et al., 2017; Pendleton et al., 2012). Second, macroalgal biomaterials can be exported to the deep ocean, where extremely slow rates of mixing with surface waters mean that carbon is also likely to be sequestered (Chang et al., 2024; Filbee‐Dexter et al., 2024; Fischer & Wiencke, 1992; Kokubu et al., 2019; Schimani et al., 2022). Even so, quantifying sequestration of carbon in the deep sea comes with a number of uncertainties due to different rates and patterns of mixing that occur across ocean basins (Baker et al., 2022; Nowicki et al., 2022; Siegel et al., 2023). Last, macroalgal‐derived DOC may be sequestered within the ocean if that DOC avoids microbial‐ and photo‐degradation, persisting in seawater for timescales relevant to carbon sequestration (Chen et al., 2020; Feng et al., 2022; Hurd et al., 2022; Shank et al., 2010; Wada et al., 2015). Macroalgae may be important contributors to a substantial oceanic carbon reservoir of recalcitrant DOC (RDOC; Shen & Benner, 2018), which remains relatively stable over time and is comparable in size to the atmospheric carbon pool (Dittmar et al., 2021; Hansell et al., 2009). Although this pool of DOC is derived from a variety of sources, macroalgae are known to be important contributors to the coastal DOC pool (contributing up to 20% of total coastal DOC concentrations; Paine et al., 2021; Wada & Hama, 2013), and vegetated coastal ecosystems are one of the largest sources of marine DOC (Barrón & Duarte, 2015; Bauer & Druffel, 1998; Wagner et al., 2020). Therefore, although macroalgae are likely important contributors to the oceanic pool of RDOC, more study is needed to directly quantify the extent of their contribution. An important note is that microbes can also transform labile marine DOC into RDOC, under the microbial carbon pump hypothesis (Feng et al., 2022; Jiao et al., 2010; Ogawa et al., 2001). Thus, macroalgal‐produced DOC might not be inherently recalcitrant but may still ultimately be sequestered as RDOC after transformation by the microbial community (Jiao et al., 2010; Legendre et al., 2015).

The rate of and magnitude at which macroalgal‐derived carbon is sequestered through these pathways is currently poorly resolved (Krause‐Jensen et al., 2018; Pessarrodona et al., 2023). One key knowledge gap that hinders our ability to estimate the carbon sequestration potential of macroalgae is a lack of understanding of the patterns and drivers of macroalgal biomaterial degradation (Dolliver & O'Connor, 2022a; Paine et al., 2021). Like all organic materials, macroalgal biomaterials, once released, are susceptible to degradation/decomposition through microbial degradation (Boldreel et al., 2023; Feng et al., 2022; Morrison et al., 2017; Rieper‐Kirchner, 1989) and/or photodecomposition (Huang et al., 2023; Shank et al., 2010; Wada et al., 2015). Organic matter degradation is defined as the conversion of complex organic molecules contained within organic matter into simpler, inorganic molecules (LaRowe & Van Cappellen, 2011; Middelburg, 1989; Oades, 1989). In the context of carbon sequestration, the conversion of organic carbon into inorganic carbon via degradation means that inorganic carbon is susceptible to re‐introduction to the atmosphere (Lal, 2007). Macroalgal‐derived biomaterials are also susceptible to ingestion by grazers; this process also interacts with degradation dynamics (Bedford & Moore, 1984; Catenazzi & Donnelly, 2007; Gómez et al., 2018; Kotta et al., 2010; Kristensen & Mikkelsen, 2003; Salathe & Riera, 2012). Therefore, in order to assess the carbon sequestration potential of macroalgae, we need to understand whether macroalgal biomaterials can either resist degradation or ingestion for timescales relevant to climate change amelioration (i.e., 100+ years; Moreda et al., 2024) or for however long it takes for the biomaterials to reach areas conducive to carbon sequestration (e.g., soft sediment habitats or the deep ocean).

The degradation of organic matter is often best explained by an exponential decay model, which is characterized by an initial period of rapid biomaterial loss sometimes referred to as the leaching period, wherein labile, easily accessible portions of biomaterial (such as carbohydrates and amino acids) are quickly utilized by microbes (Berg & Laskowski, 2005; Wider & Lang, 1982). Subsequently, the degradation rate rapidly declines after the highly labile components are digested, leaving behind recalcitrant fractions of biomaterial that are harder for microbes to attack (de Bettignies et al., 2020; Jenny et al., 1949; Yang & Janssen, 2001). Recalcitrant organic matter is defined as organic matter which persists in a system, although the length of time for which the organic matter must resist degradation for it to be deemed recalcitrant varies substantially across and within different disciplines (Hansell, 2013; Kleber, 2010; McCann & Carpita, 2015). This recalcitrant portion of biomaterial may either have molecular characteristics that hinder microbial degradation—for example, by requiring many enzymes and/or a high activation energy to break down—or the recalcitrant biomaterial may be protected from microbial attack due to physical separation from microbes, environmental drivers, microbial food saturation, or inadequate microbial population and/or diversity (Dungait et al., 2012; Kleber, 2010; Kleber et al., 2011; Marschner et al., 2008; Schmidt et al., 2011). Research has shown that long‐term recalcitrance of organic matter in terrestrial soil systems is predominately controlled by extrinsic factors rather than by the molecular structure of the organic matter itself (Amelung et al., 2008; Dungait et al., 2012; Schmidt et al., 2011). Thus, in many instances across marine and terrestrial contexts, organic matter only resists degradation under certain biotic and abiotic conditions and will continue to degrade in novel conditions (Chabbi et al., 2009; Jiao et al., 2014; Pedersen et al., 2021; Shen & Benner, 2018).

In macroalgae, recalcitrant portions of biomaterial are often made up of structural cell wall compounds which may be difficult for microbes to digest; these compounds include polyphenolics, ulvans, alginates, carrageenan, and fucoidan (Adair et al., 2008; Becker et al., 2020; Bligh et al., 2022; Deniaud‐Bouët et al., 2017; Domozych, 2019; Imran et al., 2017; Shukla et al., 2016; Sichert et al., 2020). These compounds are absent from the cell walls of terrestrial plants, which are instead made up of cellulose, hemicellulose, pectin, and lignin (Domozych et al., 2012; Niklas et al., 2017; Popper et al., 2011). These unique algal polymers have a variety of functions, including protection against grazing and/or microbial attack, osmotic and/or ionic regulation, and protection from UV damage (Deniaud‐Bouët et al., 2017; Holzinger et al., 2015; Kloareg & Quatrano, 1988; Oren, 2007; Shtein et al., 2018). Brown, green, and red macroalgae have distinct cell‐wall polymers, likely attributable to their distinct evolutionary histories (Ciancia et al., 2020; Lee & Ho, 2022; Popper et al., 2011; Synytsya et al., 2015). Brown algae, in particular, contain many unique compounds that deter microbial attack and grazing, meaning that they are thought to be harder to degrade and, thus, may be more important contributors to marine carbon sequestration compared to other algal groups (Arnosti, 2011; Deniaud‐Bouët et al., 2017; Littler et al., 1983). These compounds include polyphenolics (including phlorotannins), laminarin, fucoidan and alginate, which are all highly resistant to microbial degradation, requiring complex and highly specialized suites of enzymes to degrade (Alderkamp et al., 2007; Armstrong & Patel, 1994; Bligh et al., 2022; Buck‐Wiese et al., 2023; Sichert et al., 2020; Targett et al., 1992; Zhang et al., 2021).

Assessing the relative contributions of various groups of macroalgae to marine sediments and/or the deep sea (through eDNA and/or stable isotope modeling approaches) can also give insights into the relative stability of different types of macroalgae. One study that characterized the prevalence of macroalgal DNA across a range of ocean depths around the world observed that red algae were the most prevalent overall (Ortega et al., 2019). However, other studies with more narrow geographic scopes have observed that brown algae are typically more prominent in sediments and/or the deep sea, as compared with red and green algae (Erlania et al., 2023; Kokubu et al., 2012; Ørberg et al., 2023; Queirós et al., 2023), and one study noted that green algae were the most common type of macroalgae in seagrass‐associated sediments (Arina et al., 2023). These trends, however, are likely somewhat influenced by the abundance of algal groups in certain areas and are likely not solely indicative of the relative stability of various algal groups (Erlania et al., 2023; Ortega et al., 2019).

There are many additional reasons why understanding macroalgal degradation dynamics is an important pursuit, apart from the carbon sequestration potential of macroalgae. First, the rate at which macroalgal biomaterial degrades has important implications for ecosystem functioning, as it impacts the flow of carbon, nitrogen, and other nutrients contained within the biomaterial both within and beyond macroalgal‐dominated ecosystems (Hanisak, 1993; Luo, Dai, et al., 2022; Mellbrand et al., 2011; Pedersen & Johnsen, 2017; Renaud et al., 2015). Macroalgal detritus is an important food source for various meio‐ and macrofaunal communities (Alkemade & Van Rijswijk, 1993; Chown, 1996; Hosen et al., 2011; Kristensen & Mikkelsen, 2003; Nedzarek & Rakusa‐Suszczewski, 2004), and decaying macroalgae can often harbor and support a diverse array of meiofauna (Duggins et al., 2016; Hwang et al., 2023; Olabarria et al., 2007; Rieper‐Kirchner, 1990; Urban‐Malinga & Burska, 2009). For instance, the input of beach‐cast macroalgal wrack to sandy beaches is a crucial source of nutrients and habitat for these ecosystems, since sandy beaches usually have little in situ primary production and little three‐dimensional habitat (Colombini et al., 2000; Griffiths et al., 1983; Hyndes et al., 2022; Ince et al., 2007; Salathe & Riera, 2012).

Other timely reasons to study marine macroalgal degradation dynamics include understanding the ecological ramifications of macroalgal blooms (Castaldelli et al., 2003; Conover et al., 2016; Lanari, Copertino, et al., 2018), non‐native macroalgal invasions (Haram et al., 2020; Krumhansl & Scheibling, 2012a; Lozada et al., 2022; Pedersen et al., 2005; Rodil et al., 2008), and large scale seaweed farming (Dolliver & O'Connor, 2022; Li et al., 2022; Luo et al., 2021, 2024), all of which are increasing in occurrence and intensity worldwide (Cai et al., 2021; Seebens et al., 2017; Xing et al., 2015; Zhang, Liao, et al., 2022). Furthermore, the replacement of kelp and fucoid‐dominated ecosystems with turf algae‐ and crustose coralline algae‐dominated systems is becoming increasingly common due to anthropogenic stressors (including eutrophication, ocean warming, and epiphytism; Filbee‐Dexter & Wernberg, 2018; Harrold & Reed, 1985; Krumhansl et al., 2016; Matsunaga et al., 1999; Rogers‐Bennett & Catton, 2019; Smith et al., 2024). Thus, understanding if there are differences in degradation dynamics across different macroalgal functional groups will give insight into the ecological ramifications of current and potential future changes to macroalgal community structures.

Despite being an ecologically important and relatively well‐studied process, there has yet to be a comprehensive, systematic review published on marine macroalgal degradation dynamics. This review synthesizes trends within the body of peer‐reviewed literature on marine macroalgal degradation to ascertain drivers of macroalgal biomaterial degradation and better understand the longevity and potential recalcitrance of macroalgal carbon. To this end, the rate of degradation was verified for 504 observations of macroalgal biomaterial degradation across 105 studies, and the trajectory of degradation was verified wherever possible. This enabled assessments of macroalgal half‐lives (i.e., the time it takes for 50% of initial biomaterial to degrade) and percent recalcitrance (i.e., relative stability; see Glossary). We then used our half‐life and percent recalcitrance estimates to statistically assess the influence of methodological and environmental factors on macroalgal degradation. Importantly, we have also summarized the results of studies that independently investigated the roles of various drivers on macroalgal degradation. We have also reported trends in study motivation, study location, macroalgal type, biomaterial type, and various experimental design choices across the body of literature. Lastly, we have highlighted key remaining knowledge gaps, suggested future directions, and recommended methodologies and approaches for future study, with a particular focus on macroalgal carbon sequestration potential. This review will improve our understanding of the ultimate fate of macroalgal‐derived carbon in the ocean, understanding that is needed in order to ascertain the contribution of macroalgae to marine carbon sequestration. In addition, understanding the drivers of degradation will provide important insights into the factors that influence the flow of macroalgal biomaterials (and the nutrients contained within those biomaterials) to other ecosystems and organisms, many of which rely on macroalgal trophic subsidies (Krumhansl & Scheibling, 2012b; Norderhaug et al., 2003; Raut & Capone, 2021).

## METHODS

### Literature search and inclusion criteria

A systematic search of published literature was conducted using the databases Web of Science Core Collection and Scopus (using a title‐abstract‐keyword search) using the following search string: “macroalga* OR seaweed OR kelp OR fucoid AND detrit* OR litter OR wrack OR POM OR DOM OR POC OR DOC AND decompos* OR degrad* OR decay.” We also conducted a secondary search in Google Scholar in which we screened the first 500 search results, out of 11,600 results. (This returned 20 unique studies we deemed appropriate for inclusion in our review.)

The titles, abstracts, and results were screened to determine if the study met our inclusion criteria (outlined in Figure S1). To be included in this study, publications needed to be peer‐reviewed, primary research on marine or estuarine macroalgal degradation, and include data on the loss of macroalgal biomaterials (in the form of tissues, POM, POC, DOM, or DOC) over time. Studies were discarded for numerous reasons, outlined in the PRISMA flowchart (Figure S1). Many studies were discarded as data pertaining to algal degradation (including an initial assessment biomaterial quantity) were not available in text, within figures, or within supplemental materials. We excluded studies that only analyzed degradation through thermogravimetric analysis or only measured changes in chromophoric dissolved organic matter concentration. We also excluded unpublished theses, preprints, and gray literature. Databases were screened by C.B. and J.K. independently, with the final literature search occurring in August 2024. (See Table S1 for details on when each search was conducted and by whom.) A full list of studies screened, included, and excluded is in Supplemental Datafiles 1, 2, and 5 (available at https://osf.io/vmtfd/files/osfstorage).

### Data extraction

We extracted macroalgal degradation data from 504 observations contained within 105 publications selected for inclusion in this review. We define an observation of macroalgal degradation as a data set that describes change in biomaterial mass or concentration, or percent loss of biomaterial, over time (i.e., an individual degradation trajectory). Where degradation data was not presented in text form, data was extracted from figures using the online software PlotDigitizer (PlotDigitizer, 2024). In most cases, the raw data were not available, so average values for each time point were extracted. Many papers did not include estimates of standard error or standard deviation and so were omitted. Data extraction was conducted by C.B. and J.K. independently. Extracted degradation data can be found in Supplemental Datafile 3.

All other relevant details from each study were extracted, including methodological details and biophysical parameters, and are available in Supplemental Datafile 1. We recorded what type of macroalgal‐derived biomaterial the authors of each study measured the decay trajectory of, based on whether study authors stated that they measured the degradation of: macroalgal tissue, carbon content within macroalgal tissue, POM, POC, or DOC. See Glossary for a definition of the various types of biomaterials. Since the definition of POM/POC and DOM/DOC can vary, for observations of POM/POC degradation, we recorded what size range of particles a study classified as being POM/POC, and for studies interested in DOC degradation, we recorded the filter pore size used by study authors to quantify DOC in water samples. Next, we classified studies on the basis of what the authors stated their motivation was for conducting their study, as described either in the abstract and/or discussion, binning studies into six categories of study motivations outlined in Table 1. Also, we noted if the authors chose to pre‐treat or pre‐kill macroalgae prior to the start of the degradation experiment. If so, we noted how they pre‐treated the algae. (Pre‐treatment methods included freezing the algae, placing the algae in a hot water bath, or drying the tissue out.) Other relevant study parameters we extracted included but were not limited to: study location (ex situ or in situ, latitude and longitude, study location characteristics) and methodological details (macroalgal species, study duration, number of replicates, temperature, light level, and others). We also recorded data on changes in carbon content, nitrogen content, and/or carbon: nitrogen ratios in macroalgal detritus throughout degradation, if authors measured and reported these data (Supplemental Datafile 4).

**TABLE 1:** Proportions of studies included in this review that fit into six broad categories of study motivation, along with the definition for each study motivation category.

Study motivation category	Category definition	Percentage of studies belonging to this category
Nutrient and/or heavy metal release and cycling	Many studies stated that understanding nutrient release rates (including carbon, nitrogen, phosphorous and others) during degradation was their main motive. This also includes authors interested in the rates of heavy metal release during macroalgal decay	29%
Meiofaunal community dynamics and/or impact of meiofauna on degradation	Authors were primarily interested in either understanding how wrack acts as a habitat for small invertebrates and/or how this meiofaunal community impacts algal degradation dynamics	19%
Carbon sequestration potential (CSP) of macroalgae	Studies' with the primary motivation to better understand the CSP of macroalgae by characterizing degradation dynamics (26% of studies identify macroalgal CSP at least once throughout their paper, often in the discussion to contextualize their results)	18%
Cross‐species comparison	Studies' with the central aim to compare how degradation rates changed across various species of macroalgae and/or aquatic plants. For example, comparing the degradation of invasive algal species to native species or bloom‐forming algae to longer‐lived algae	17%
Microbial community dynamics and/or impact of microbes on degradation	Studies that primarily focused on characterizing the microbial community throughout degradation and/or describing how microbes impact degradation	12%
Impact of abiotic factors on degradation	These abiotic factors include temperature, depth, oxygen availability, salinity, and others	6%

*Note*: Study motivation refers to what the authors state in their abstract and/or introduction as being their motive for conducting their research.

All taxonomy was confirmed and updated using the World Register of Marine Species (https://www.marinespecies.org/index.php). For analytical purposes, macroalgae were partitioned into functional groups including “kelps” from the order Laminariales (Jayathilake & Costello, 2020); “fucoids” from the order Fucales; “turf” including low‐lying, ephemeral, densely aggregating macroalgae (see Connell et al., 2014); and “foliose” macroalgae (i.e., macroalgae larger than turf and not within the kelp or fucoid functional groups; Steneck & Dethier, 1994). We also recorded macroalgal classification, which refers to whether the species is a brown algae (Class Phaeophyceae), green algae (Phylum Chlorophyta), or red algae (Phylum Rhodophyta). See Supplemental Datafile 6 for a full list of the 99 species of macroalgae included in this review and the corresponding functional group and classification we reported each species as.

### Data analysis

#### Verifying the trajectory of degradation within algal degradation studies

Across macroalgal degradation studies, two common models were used to describe biomaterial decomposition rate: exponential decay and linear loss. Exponential decay provides a decay constant or *k*‐value (Equations 1 and 2), and linear loss provides a constant degradation rate that estimates the percent loss per day (Equation 3). When presenting degradation data, some authors verified the best model fit for their data and then presented the corresponding summary statistic, while others presented both values, or just one value without verification of the best fit. For this reason, the trajectory of degradation was re‐analyzed for all observations collected in this study to determine what model best fit the data.

**FIGURE 1:**
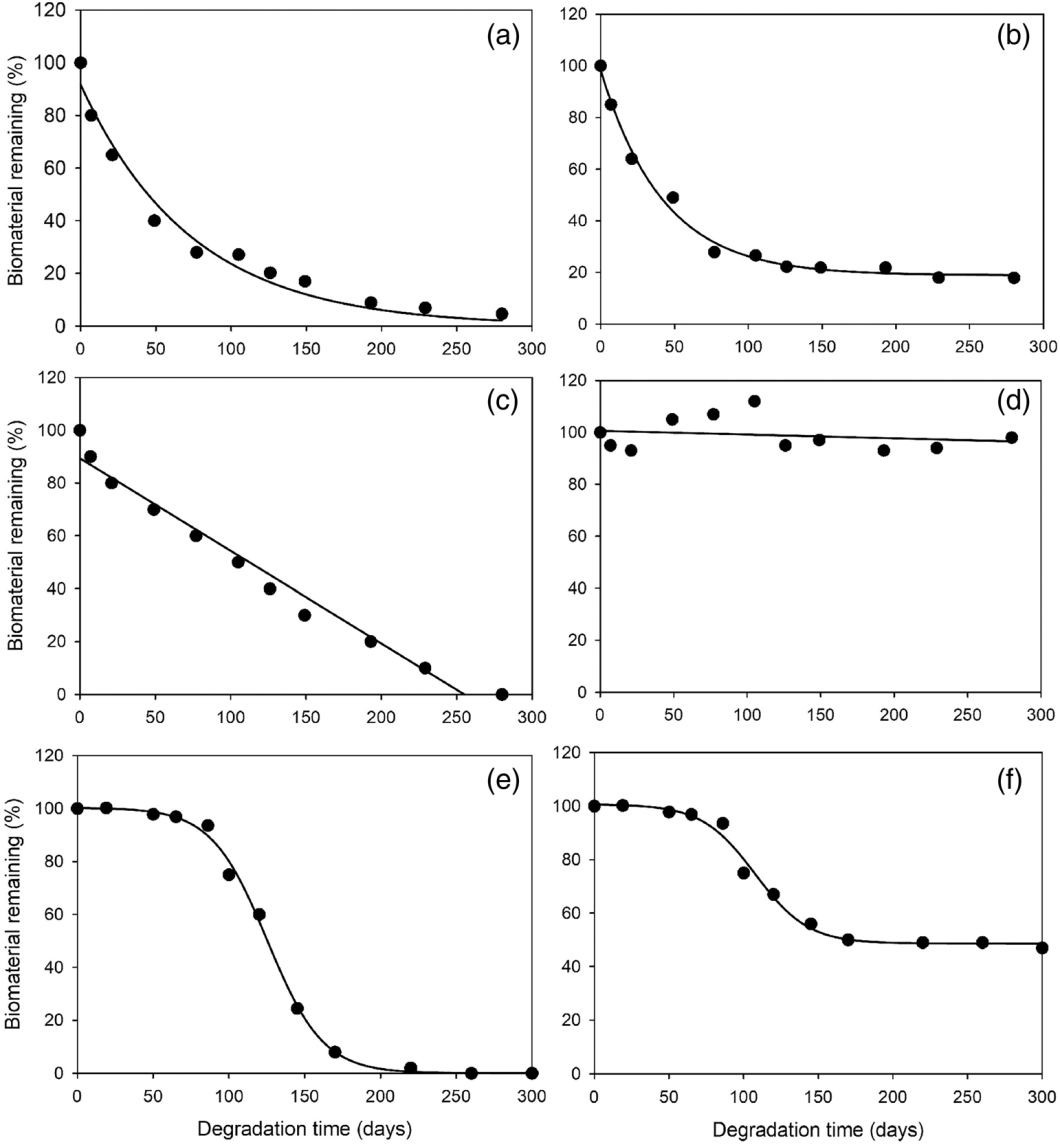
Example data plots which illustrate the types of models used to describe macroalgal decay in this review paper. (a) Simple (two‐parameter) exponential decay, (b) three‐parameter exponential decay, (c) linear loss, (d) confounded by growth (meaning no model could be fit to the data), (e) three‐parameter sigmoidal model, and (f) four‐parameter sigmoidal model.

Degradation trajectories could not be calculated in some cases. First, in some cases macroalgal growth (i.e., increase in biomass/concentration) outweighed/negated any signs of decay, meaning that degradation trajectory could not be easily described, as in these instances teasing apart growth and degradation was not possible (Figure 1d). These observations were designated as being “confounded by growth.” Furthermore, decay trajectories could not be verified if degradation was only assessed from an initial and final value. In these cases, the trajectory of degradation was assumed to be linear. In cases where study authors only included degradation rates and/or *k*‐values without including any raw degradation data, degradation trajectory verification was not possible, so only reported degradation rates and/or *k*‐values were used in our analyses.

For studies with at least two sampling events (plus an initial sample), we were able to characterize the degradation trajectory. First, units for biomass/concentration were converted into percent of the initial value remaining, if not done already. Using Sigmaplot's regression Wizard, we tested various curves for each data set to determine which curve had the highest *R* ^2^ value and, therefore, best described the data. We tested linear, sigmoidal, and exponential decay models for each data set (see Figure 1 for example data and fitted model types). We endeavored to avoid overfitting the data by avoiding adding unnecessary parameters, particularly for data sets with a limited number of sampling events. All extracted data, model types, equations, and associated *R* ^2^ values can be found in Supplemental Datafile 3.

Five model types were used to describe degradation data in this review:

1. Two‐parameter exponential decay model (Equation 1; Figure 1a): characterized by an initial period of rapid degradation, followed by a period of slower degradation until the curve reaches zero, where *y*_0_ = initial value, *k* = decay constant, *t* = degradation time in days.

(1) $$y=y_{0}e^{-\mathrm{kt}}$$

2 Three‐parameter exponential decay model (Equation 2; Figure 1b): an exponential decay function (as described above) where the curve never reaches zero and instead levels off at a horizontal asymptote (representing a portion of biomass that is considered recalcitrant). Here *R* = the asymptote (i.e., percent which is recalcitrant, if the asymptote is positive), *a* = initial value‐*R*, *k* = decay constant, *t* = degradation time in days.

(2) $$y={\mathrm{ae}}^{-\mathrm{kt}}+R$$

3 Linear model (Equation 3; Figure 1c): characterized by a relatively constant degradation rate throughout the experiment duration. Here *y*_0_ = initial value, *d* = degradation rate (in percent change per day), *t* = degradation time in days.

(3) $$y=y_{0}+\mathrm{dt}$$

4 Three‐parameter sigmoidal model (Equation 4; Figure 1e): characterized by a period of negligible degradation at the beginning of the experiment, then the degradation rate increases to an inflection point, after which degradation rate decreases. Here *a* = maximum degradation rate, *c* = time point of maximum degradation rate, *s* = scaling value, *t* = degradation time in days. Note: For data described by sigmoidal models, we calculated the “next best fit” by testing what model has the next‐highest *R* ^2^ value, so that we could estimate half‐life from these data.

(4) $$y=\frac{a}{1+e^{\left(t-c\right)/s}}$$

5 Four‐parameter sigmoidal model (Equation 5; Figure 1f): a sigmoidal curve (as described above) but with a positive horizontal asymptote, representing a portion of recalcitrant material. Here *a* = maximum degradation rate, *c* = time point of maximum degradation rate, *s* = scaling value, *t* = degradation time in days, *R* = the asymptote, or portion of recalcitrant material.

(5) $$y=\frac{a}{1+e^{\left(t-c\right)/s}}+R$$

#### Calculating macroalgal half‐life and percent recalcitrance

We estimated the half‐life of macroalgal biomaterial (i.e., time it takes in days for 50% of initial biomaterial to disappear) either using the formula half‐life = 50/degradation rate for linear relationships, or half‐life = ln(2)/*k* for two‐parameter exponential decay models. For data best described by three‐parameter exponential decay models, *y* = 50 was used in the best‐fit equation and then *t* was solved for. For sigmoidal curves, we used the next best fit model (i.e., next highest *R* ^2^ value) to calculate the half‐life. Where the asymptote value was greater than half of the initial material, the half‐life could not be calculated.

Determining what portion of macroalgal biomaterial is recalcitrant can be difficult since it is highly dependent on the duration and methodology of the experiment. Although researchers might report that a portion of initial biomaterial remains after their experiment concludes, if monitored for longer or exposed to different environmental conditions, the remaining biomaterial may continue to degrade (Figure S2). However, if the data follow a three‐parameter exponential decay model or a four‐parameter sigmoidal model with a positive *R* value (i.e., positive horizontal asymptote; Equations 2 and 5), there is likely a recalcitrant portion of biomaterial since the degradation rate has leveled off. Thus, we estimated percent recalcitrance as the value of the horizontal asymptote in cases where degradation data were best described by a model with a horizontal asymptote.

We assessed that there was zero percent recalcitrance if (a) there was no biomaterial left when the experiment ended or (b) the experiment duration was longer than 1 month and the degradation data were *not* best described by a model with a horizontal asymptote. We chose 1 month as a cut‐off after visually inspecting all degradation curves that followed a three‐parameter exponential decay model and observed that the overall average approximate time it took for the degradation rate to approach zero was 1 month. We were unable to confidently assess whether there was a portion of recalcitrant biomaterial in the following cases: (a) if the study did not provide degradation data and only provided an estimate of degradation rate and/or *k* value, (b) if the study only measured initial and final biomass/concentration *and* there was still remaining biomaterial when the experiment ended, and (c) if the degradation trajectory was *not* best described by a model with a positive horizontal asymptote *and* the experiment duration was less than 1 month *and* there was still remaining biomaterial at the end of the experiment.

#### Statistically analyzing trends across studies

All statistical analyses were performed in R (v4.2.3, R Core Team, 2023). To assess the potential influence of environmental and methodological factors on macroalgal half‐life (in days), we ran a multivariate linear mixed model (LMM) with study included as a random effect, using the lme4 package (Bates et al., 2015). Predictor variable selection was performed using the best subset regression modeling approach, based on Akaike information criterion (AIC) and Mallow's Cp values, using the package olsrr (Hebbali, 2024). We only considered the inclusion of predictor variables for which we had an a priori hypothesis as to why these factors may influence macroalgal degradation (see Table 2). We also ensured that none of the potential predictor variables were significantly correlated with each other to avoid multicollinearity in our model. This resulted in the selection of the following variables for inclusion in the half‐life LMM: algal functional group, algal class, experimental temperature (°C), pre‐treatment, litterbag mesh diameter (mm), degradation environment, and light availability (see Table 2 for a specification of categorical variable levels, groups means, and sample sizes). Half‐life and litterbag mesh size data were both log_10_ transformed prior to analysis. Four studies which analyzed the degradation of algal polycultures (i.e., a mixture of various algal species) were excluded from the analyses of functional group and algal class (by coding these cells as NAs in our data frame). Also note that since very few studies reported light intensity, we analyzed the effect of light as a binary variable (i.e., light was available or the experiment was done in darkness). Additionally, despite the fact that there are many different methods of pre‐treating macroalgae, we chose to analyze pre‐treatment as a binary variable since most pre‐treatment types were represented by very few studies. To understand the impacts of categorical variables on half‐life and percent recalcitrance, the emmeans package was used to extract pairwise contrasts from our models (Lenth, 2024). Model diagnostics for all models were assessed using the DHARMa package (Hartig, 2022).

**TABLE 2:** List of environmental and methodological factors that were investigated using statistical and qualitative approaches in this review.

Factor	A priori hypothesis	Half‐life (in days) group means ± standard error (sample size)	Percent recalcitrance (%) group mean ± standard error (sample size)
Biomaterial type^a^	Many studies have shown marine DOC from a variety of sources to be considerably recalcitrant, we might expect macroalgal derived DOC to be more recalcitrant and/or degrade slower than tissues or particulate matter	POC = 43.86 ± 12.92 (*n =* 16) POM = 37.45 ± 10.31 (*n =* 15) DOC = 11.8 ± 2.95 (*n =* 34) Tissue = 59.31 ± 6.70 (*n =* 349)	POC = 40.72 ± 18.62 (*n =* 4) POM = 42.77 ± 5.88 (*n =* 18) DOC = 30.88 ± 4.280 (*n =* 32) Tissue = 15.52 ± 1.85 (*n =* 166)
Functional group	Different functional groups of macroalgae differ in their cellular composition which may impact how they degrade	Foliose = 58.89 ± 13.88 (*n =* 49) Fucoid = 35.97 ± 9.78 (*n =* 76) Turf = 27.14 ± 3.04 (*n =* 118) Kelp = 74.74 ± 1.02 (*n =* 167) Removed: 14 “Mixed” observations	Foliose = 14.39 ± 4.072 (*n =* 32) Fucoid = 33.98 ± 4.469 (*n =* 38) Turf = 18.82 ± 2.906 (*n =* 80) Kelp = 18.46 ± 2.552 (*n =* 83) Removed: 1 “Mixed” observation
Classification	Taxonomic similarities within groups may drive differences in susceptibility to degradation	Red = 23.5 ± 3.99 (*n =* 62) Green = 26.7 ± 3.85 (*n =* 73) Brow*n* = 64.73 ± 8.26 (*n =* 276) Removed: 14 “Mixed” observations	Red = 14.29 ± 4.218 (*n =* 29) Green = 19.83 ± 3.501 (*n =* 53) Brown = 22.01 ± 2.114 (*n =* 151) Removed: 1 “Mixed” observation
Average reported temperature across experiment duration (°C)	Higher temperatures accelerate decomposition in terrestrial systems, so we expect that macroalgae will degrade quicker at higher temperatures	*n =* 319	*n =* 172
Was the detritus pre‐treated?	Pre‐killing algae is expected to kickstart the degradation process, resulting in faster degradation rates	Yes = 25.31 ± 2.77 (*n =* 190) No = 71.3 ± 9.48 (*n =* 236)	Yes = 25.49 ± 2.852 (*n =* 78) No = 17.95 ± 2.028 (*n =* 156)
Degradation environment	The environment where degradation takes place affects the type of microbes available to degrade algae, as well as various abiotic factors, which may impact degradation rate	Suspended = 55.78 ± 15.57 (*n =* 52) Intertidal = 33.4 ± 5.23 (*n =* 64) Seafloor = 68.31 ± 15.13 (*n =* 120) Lab = 44.2 ± 6.24 (*n =* 190)	Suspended = 9.72 ± 3.487 (*n =* 37) Intertidal = 30.22 ± 4.523 (*n =* 37) Seafloor = 8.06 ± 2.492 (*n =* 60) Lab = 28.27 ± 2.519 (*n =* 100)
Mesh size of detritus litterbag (in mm)	Litterbags made from smaller diameter mesh (i.e., more tightly woven mesh) may reduce the amount of contract that the detritus has with grazers and/or microbes and may reduce the amount of light which can penetrate the litterbag. Both of these factors may impact degradation	*n =* 274	*n =* 148
Experiment duration (in days)^a^	Since organic matter typically exhibits a rapid rate of degradation during the initial phases of degradation, shorter studies are expected to report faster degradation rates as compared to studies with longer durations which are expected to capture the final, slower stages of degradation	*n =* 426	*n =* 234
Light availability	If light is available, detritus is able to continue to photosynthesize, delaying degradation	Dark = 33.18 ± 3.39 (*n =* 158) Lit = 66.28 ± 9.23 (*n =* 244)	Dark = 26.63 ± 5.364 (*n =* 82) Lit = 13.16 ± 2.270 (*n =* 135)

*Note*: We also present an a priori hypothesis as to why each factor might impact macroalgal degradation. For categorical variables, we list the group mean values ± standard error (sample size) for both half‐life and percent recalcitrance.

^a^ The impact of biomaterial type on degradation could not be analyzed statistically, and the effect of experiment duration was only analyzed in terms of its effect on macroalgal percent recalcitrance, not half‐life.

We performed a similar model‐selection process (best subset regression modeling based on AIC and C(p) values using the package olsrr; Hebbali, 2024) to analyze the effects of various factors on macroalgal percent recalcitrance (%). Since the percent recalcitrance data were highly zero‐inflated, we analyzed these data using a multivariate generalized linear mixed effects model (GLMM) with a zero‐inflated negative binomial distribution using the glmmTMB package (Brooks et al., 2017). The following variables were selected for inclusion in the percent recalcitrance GLMM: experimental temperature (°C), litterbag mesh diameter (mm; log_10_ transformed), experiment duration (in days), light availability, pre‐treatment, algal functional group, algal class, and degradation environment (see Table 2). Study was also included as a random effect in this test.

Finally, because only select studies measured macroalgal carbon content, nitrogen content, and/or carbon:nitrogen ratios, we analyzed these variables in a separate test to determine if they predicted changes in macroalgal half‐life. We used a linear mixed effects model (using the lme4 package; Bates et al., 2015) to analyze the impact of initial percent carbon (%), initial percent nitrogen (%), and initial carbon:nitrogen ratio on macroalgal half‐life, with study included as a random effect.

## RESULTS AND DISCUSSION

### Motivations for studying macroalgal decay

**FIGURE 2:**
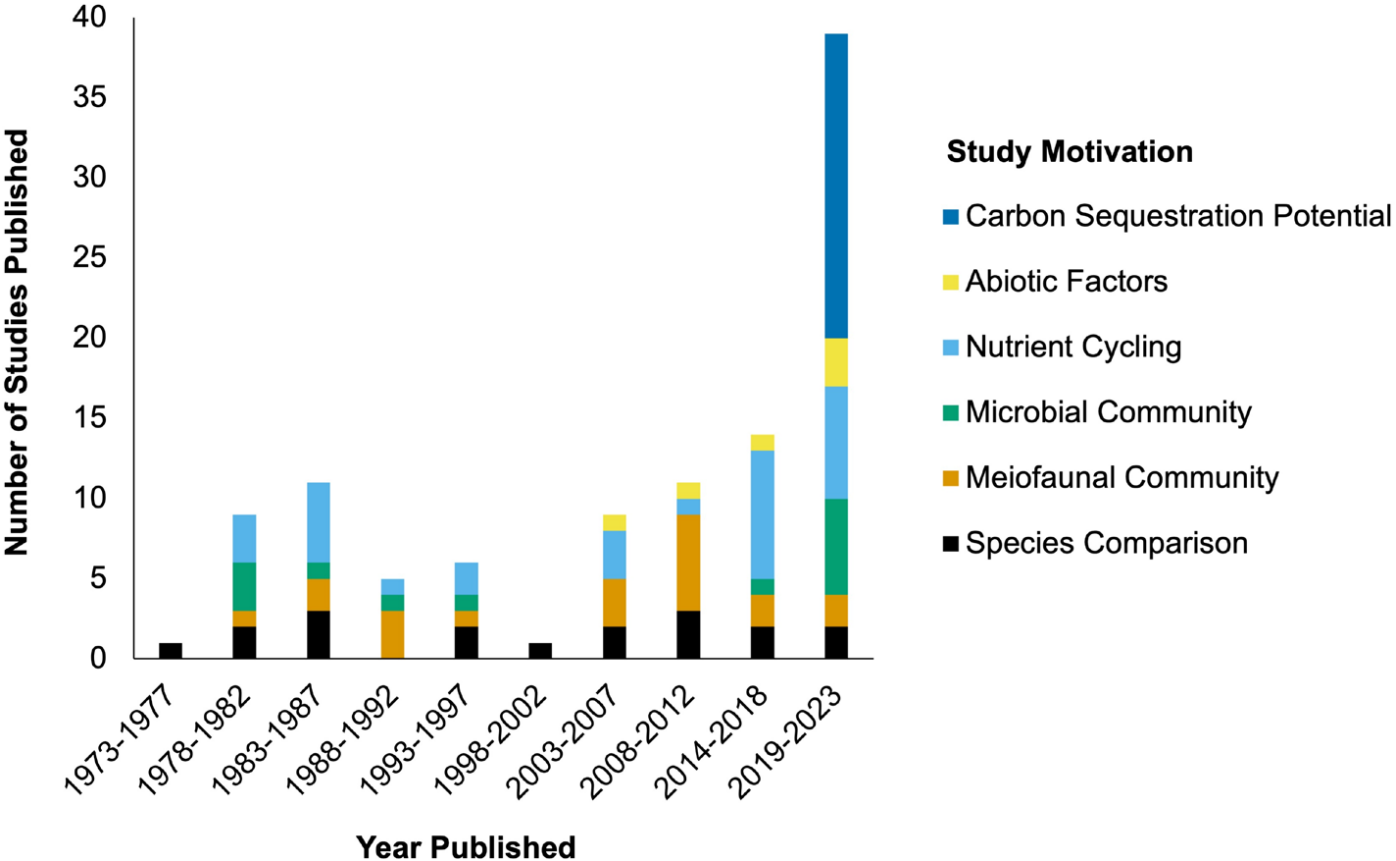
Histogram demonstrating the number of macroalgal degradation studies through time. Color‐coding represents the study motivation, meaning what the study authors state as their motive for conducting their study. Although interest in the topic of marine macroalgal degradation has been quite steady throughout time, there has been a recent uptick in studies that state that understanding macroalgal carbon sequestration potential (CSP) was a main aim of their study (*N* = 105 studies). Studies are binned into 5‐year periods based on publication date.

There has been sustained interest in the topic of macroalgal decomposition since 1976. Throughout this time, authors have described a variety of motives for studying macroalgal degradation dynamics, with the most common reasons for studying this topic being investigating nutrient and/or heavy metal release and cycling, the impact of meiofauna/changes to meiofaunal community throughout degradation, and the carbon sequestration potential of macroalgae (Table 1). The recent spike in publications on this topic is largely attributable to the current surge in interest in understanding the carbon sequestration potential of macroalgae (Figure 2).

### Methodological trends

**FIGURE 3:**
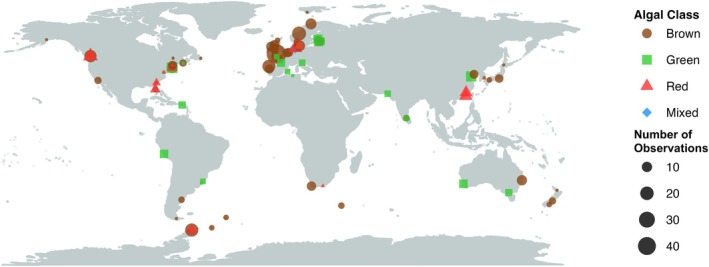
Map showing the global distribution of macroalgal degradation studies. Studies on marine macroalgal degradation have been conducted on all continents. Although there is a bias toward Europe and the East coast of North America (*N* = 504 observations). Larger sized points indicate a higher number of overlapping observations at that location. Algal class is depicted as differently shaped and colored points.

**FIGURE 4:**
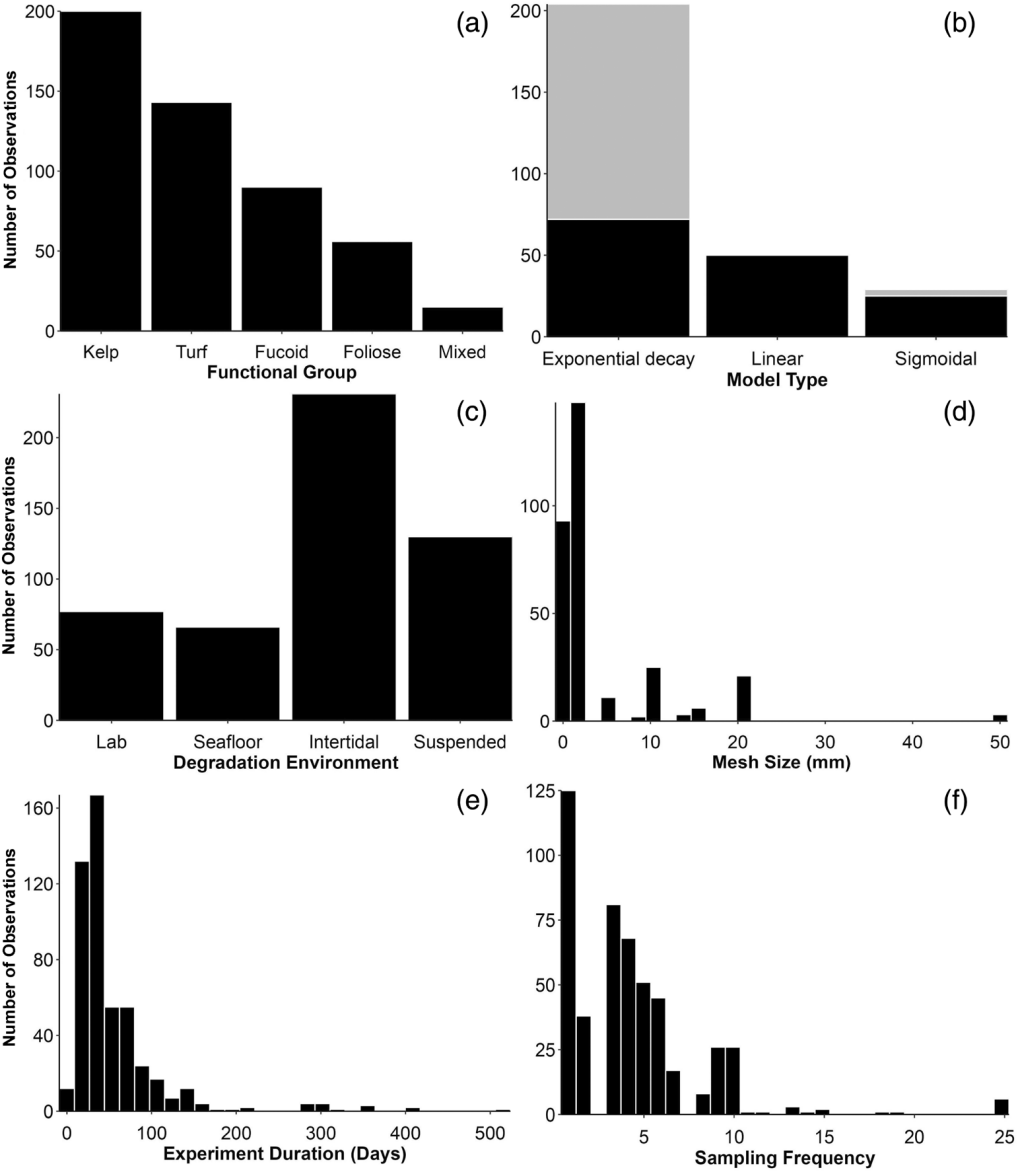
Frequency distributions of characteristics of macroalgal degradation studies. (a) macroalgal functional group, (b) general model type which best describes the degradation data, where gray coloring indicates models with a positive horizontal asymptote (indicating a recalcitrant portion of biomaterial), (c) habitat where degradation took place; in a lab or in situ either submerged on the seafloor, in the intertidal zone, or suspended in the water column, (d) mesh size (in mm) of bags containing macroalgal tissues (if applicable), (e) experiment duration in days, and (f) number of sampling events (not including the initial sample).

Although the body of knowledge on the topic of macroalgal degradation is large, research efforts have not been evenly spread across geographic regions, macroalgal groups, or biomaterial types. Geographically, although macroalgal degradation studies have been conducted on all continents, the majority of work has occurred in the Northern hemisphere (82% of all observations), with Europe being particularly over‐represented (54% of all observations; Figure 3). Kelps, perhaps the most enigmatic group of macroalgae, have been the most commonly studied macroalgal group for degradation experiments (42% of all observations; Figure 4a), followed by turf algae (27% of observations). Additionally, data regarding macroalgal tissue degradation have made up the majority of observations included in this review (81% of observations), while other biomaterials have been more commonly overlooked—only 9.5% of observations are from DOC degradation experiments and 9% of observations are from POC or POM degradation experiments.

All macroalgal exudate (DOC, POC, and POM) degradation studies included in this review were conducted in a laboratory. This reflects the logistical constraints of measuring exudate degradation in situ as constant water movements prevent experimenters from being able to realistically measure exudate degradation over time in the open water column. Containing a portion of seawater in a closed chamber in situ and measuring exudate degradation is possible and would expose the samples to natural daily light and temperature fluxes (Wada et al., 2007; Watanabe et al., 2020); however, this approach prevents water circulation, which likely influences degradation dynamics due to a lack of introduction of new microbes and/or grazers as well as the potential depletion of oxygen and/or nutrients by microbes within the chamber. Although tracer molecules such as radiolabeled isotopes, stable isotopes, or eDNA can be used to track the movement of macroalgal exudates in situ (Hill & McQuaid, 2009; Ørberg et al., 2023; Queirós et al., 2019; Sosik & Simenstad, 2013), many of these approaches are currently limited in quantitative scope and are more useful for qualitative assessments (Beng & Corlett, 2020; Fonseca, 2018; Roussel et al., 2015). For example, these methods can identify a pathway from source to sink but are limited in their ability to accurately quantify the amount of macroalgal exudates that reach a given sink (Dolliver & O'Connor, 2022a). Although the quantitative aspects of methodologies such as eDNA are improving (Lacoursière‐Roussel et al., 2016; Yates et al., 2019), there are still many logistical restraints surrounding analysis of macroalgal exudate degradation in situ.

It is also important to note that there are two general approaches experimenters have utilized to study macroalgal‐derived exudate degradation. The experimenters can assess the changes in exudate concentration as macroalgal tissue degrades in the same container, or the experimenters can take a sample of seawater containing macroalgal‐derived exudates and measure how the exudate degrades (i.e., without macroalgal tissue also present). The problem with measuring degradation of macroalgal tissue and exudates in the same container is that the leaching of exudates from the macroalgal tissue confounds the degradation of the exudates. A researcher's choice of methodology will depend on the research question asked, since isolated exudate studies will provide high‐quality data pertaining to exudate degradation; however, combined leaching and degradation studies may give a more holistic understanding of the patterns of macroalgal biomaterial release and longevity.

### Trajectory of degradation

For 37% of all observations (i.e., individual degradation trajectories), we were unable to assess the trajectory of macroalgal degradation, either because authors only assessed initial and final biomass/carbon change (25%) or because authors only included an estimate of degradation rate(s) and/or *k* value(s) without providing raw degradation data (12%). For observations wherein assessing degradation trajectory was possible, 23% followed a two‐parameter exponential decay model, 41% followed a three‐parameter exponential decay model (i.e., exponential decay with a horizontal asymptote), 11% of observations were confounded by algal growth, 9% were sigmoidal, and 16% followed a negative linear trend (Figure 4b). See Supplemental Datafiles 1 and 3 for all *R*‐squared values and model equations for each fitted model.

In the majority of cases, it was observed that an exponential decay model was the best fit for decomposition, affirming that exponential decay is usually suitable to describe macroalgal decay (Figure 4b). However, for 36% of all observations for which model fitting was possible, exponential decay was not the best fit. This demonstrates the substantial variation in the trajectories of macroalgal degradation and highlights the complexity of understanding drivers of macroalgal biomaterial longevity. This may be because, unlike most other forms of organic detritus, macroalgal biomaterial does not necessarily senesce once it is dislodged from the substrate or the main plant and can continue to photosynthesize and grow, which creates unpredictable degradation dynamics (Frontier et al., 2021; Wright & Foggo, 2021; Wright & Kregting, 2023). Indeed, we categorized 11% of observations as being confounded by growth, meaning that macroalgal biomaterials increased in biomass during the experiment, creating difficulties with data analysis (Figure 1d). The findings of these studies are still important in the context of carbon sequestration, as resisting degradation by continuing to grow may be a pathway through which algae can eventually become sequestered (Frontier et al., 2021, 2022; Wright & Foggo, 2021). More specifically, if macroalgal tissue or detached whole plants can continue to photosynthesize and grow, thereby avoiding degradation for a significant amount of time, this may increase the transport distance that those macroalgal biomaterials are able to cover, increasing the likelihood that those biomaterials end up in an area where sequestration is possible (i.e., soft sediments or the deep sea; Ager et al., 2023; Johnson & Richardson, 1977; Kokubu et al., 2019; Krause‐Jensen & Duarte, 2016). This is particularly true if the macroalgal biomaterials remain relatively buoyant (Wernberg & Filbee‐Dexter, 2018).

The prevalence of sigmoidal and linear degradation trajectories (accounting for 25% of observations for which model fitting was possible) was surprising (Figure 4b). A sigmoidal curve was the best fit for 9% of all observations, despite the fact that it is not commonly used to describe organic matter decomposition (Figure 1e,f). Here, perhaps a mixture of degradation and growth was occurring simultaneously during the first portion of the experiment, which may be why there was negligible apparent change in biomass through time for the initial period of the experiment. Then, as a greater portion of the macroalgal matter senesces, the degradation rate increases as labile components are utilized, as in exponential decay models. Alternatively, microbial colonization may be delayed in these instances, and once a critical number of microbes colonize the macroalgal matter, degradation progresses more quickly. In cases where a linear model best described the data (16%), this might indicate that microbes and consumers were able to steadily degrade macroalgal biomaterial over time, perhaps due to high microbial density and/or diversity. However, this data may only appear to be linear because there was a low number of sampling events and/or a short experiment duration, which makes it difficult to discern the “true” trajectory of degradation (Figure S2).

### Factors impacting macroalgal degradation

Overall, mean macroalgal half‐life was 50.79 ± 5.5 days (*N* = 426; Table S2), and mean percent recalcitrance (i.e., relative stability; see Glossary) was 20.44 ± 1.66% (*N* = 235). For 15% of observations, we could not estimate half‐life since more than 50% of the initial biomaterials remained once the experiment ended. Also, for the majority of observations (54%), we were not able to accurately assess recalcitrance of macroalgal biomaterials, which was due to either a lack of data or an experiment duration that was less than 1 month. Percent recalcitrance data were highly zero‐inflated; out of the 235 observations for which calculating percent recalcitrance was possible, 45% reported 0% recalcitrance. It is important to note that many studies included in this review (88% of in situ tissue degradation studies) utilized the litterbag technique (allowing macroalgal tissue to decay in a bag made of mesh); however, a shortfall of this approach is that it cannot account for the degradation of tissue fragments or exudates that escape through the gaps in the mesh, and these biomaterials may or may not, in fact, persist in the water column (Barrón et al., 2014; Krause‐Jensen & Duarte, 2016; Paine et al., 2021).

Our statistical analysis of macroalgal half‐life determined that increased temperatures predict shorter macroalgal half‐lives and that larger litterbag mesh sizes predict longer half‐lives (Table S3). Our analysis of macroalgal percent recalcitrance showed that brown algae had significantly higher mean percent recalcitrance than red algae and that pre‐treating macroalgae leads to a larger percentage of recalcitrant biomaterials (Table S4). However, the unequal and/or low sample sizes across certain groups may limit our statistical power in these tests (see Table 2). Also, the random effect of study was highly significant for the half‐life analysis (*p* < 0.00001), but there was no significant random effect of study for the percent recalcitrance model (*p* = 1). This indicates that although individual study characteristics often have a significant impact on a study's half‐life estimates, percent recalcitrance estimates do not seem to be impacted by distinct study methodologies/contexts.

#### Biomaterial type (DOC, POC, POM, or tissues)

**FIGURE 5:**
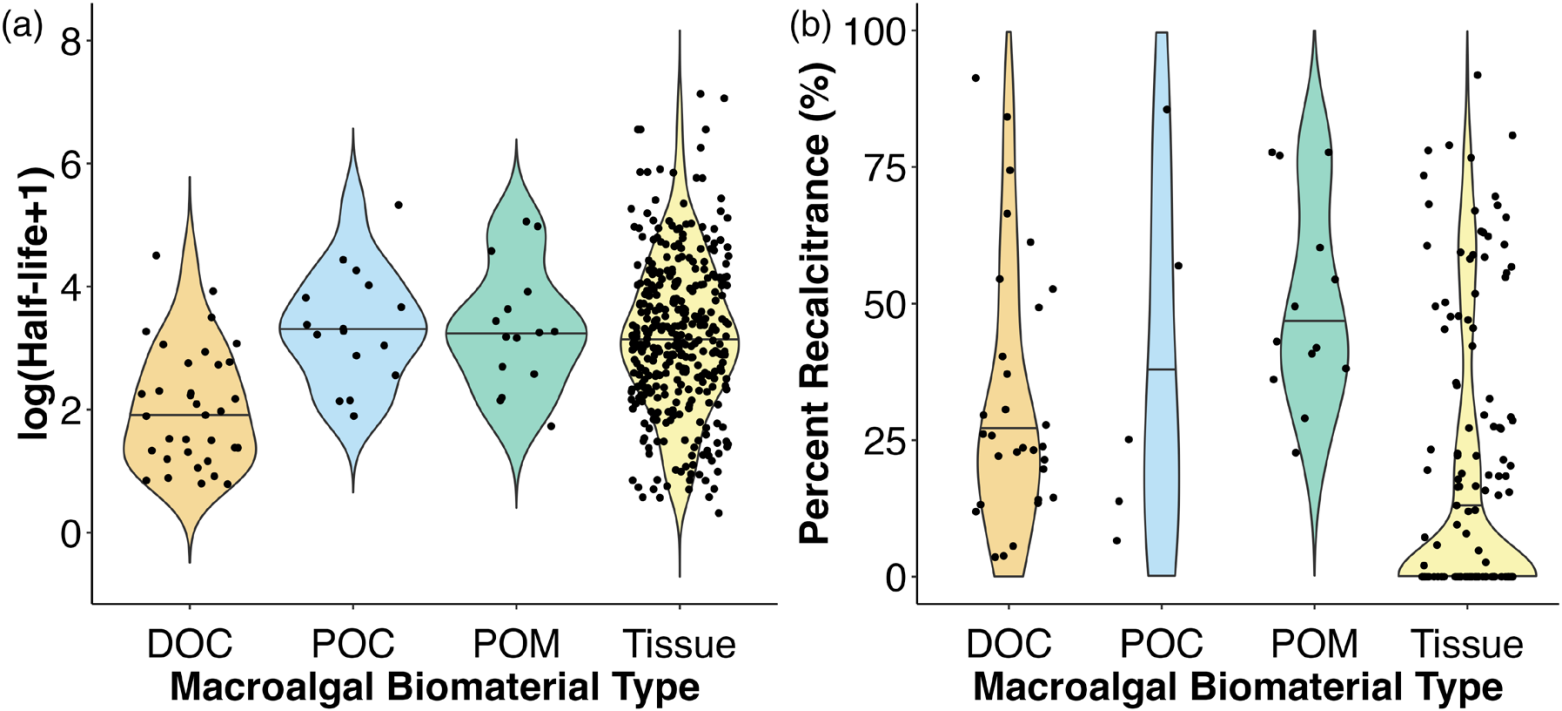
Violin plot displaying how macroalgal half‐life in days (panel a; data are log_10_ transformed) and percent recalcitrance (panel b) vary across biomaterial types (dissolved organic carbon, DOC, particulate organic carbon, POC, particulate organic matter, POM, or macroalgal tissues).

Macroalgal‐derived DOC generally decays faster than tissues, POM, and POC (as evidenced by shorter mean half‐life; Table 2; Figure 5). Although DOC, POC, and POM generally had higher mean proportions of recalcitrant biomaterial compared to tissues (Table 2) the starkly uneven sample sizes across groups and heterogeneity of variances in this data prevented us from being able to statistically analyze these data.

#### Macroalgal taxonomy

Given that different types of macroalgae have quite distinct molecular constitutions, which potentially impact susceptibility to microbial attack (Lee & Ho, 2022; Popper et al., 2011; Shtein et al., 2018), we had expected that macroalgal taxonomy would be an important predictor of degradation dynamics. However, although select studies included in this review investigated how algal taxonomy impacts algal degradation within their individual studies, their results were contradictory. One study reported that four turf algal tissue species (*Ulva compressa, U. rigida, Gracilaria vermiculophylla*, and *Agardhiella subulata*) all decayed faster than the fucoid *Fucus vesiculosus* (Conover et al., 2016). Conversely, two other studies reported that brown algae (*F. vesiculosus* and *Undaria pinnatifida*) decayed faster than turf algae (*Ulva* spp. in Hwang et al., 2023; *Corallina officinalis* and *Ulva intestinalis* in Kim et al., 2021; Table S5).

**FIGURE 6:**
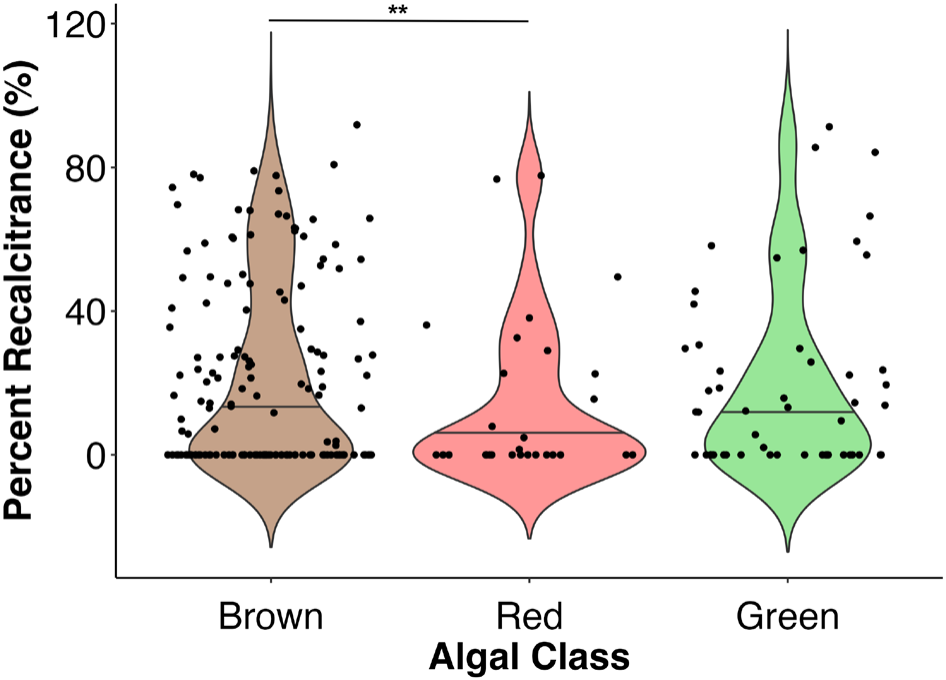
Violin plot displaying how macroalgal percent recalcitrance varies with macroalgal class. Brown algae are significantly more recalcitrant, as compared to red algae.

Importantly, both Buchsbaum et al. (1991) and Hwang et al. (2023) have reported that brown algae had higher proportions of recalcitrant biomaterial, as compared with turf algae (Table S5). Furthermore, our analysis uncovered that brown algae had significantly higher proportions of recalcitrant biomaterials, but only when compared to red algae (*p* = 0.043; brown algae – green algae *p* = 0.2639; green algae – red algae *p* = 0.6424; Table S4; Figure 6). This finding is supported by the prevalence of particularly recalcitrant compounds in brown algae, such as polyphenolics, fucoidan, and alginate (Bligh et al., 2022; Buck‐Wiese et al., 2023; Deniaud‐Bouët et al., 2014; Zhang et al., 2021). However, we did not detect a significant effect of macroalgal taxonomy on macroalgal half‐life through our analysis (Table S3; Figure S3).

#### Temperature

**FIGURE 7:**
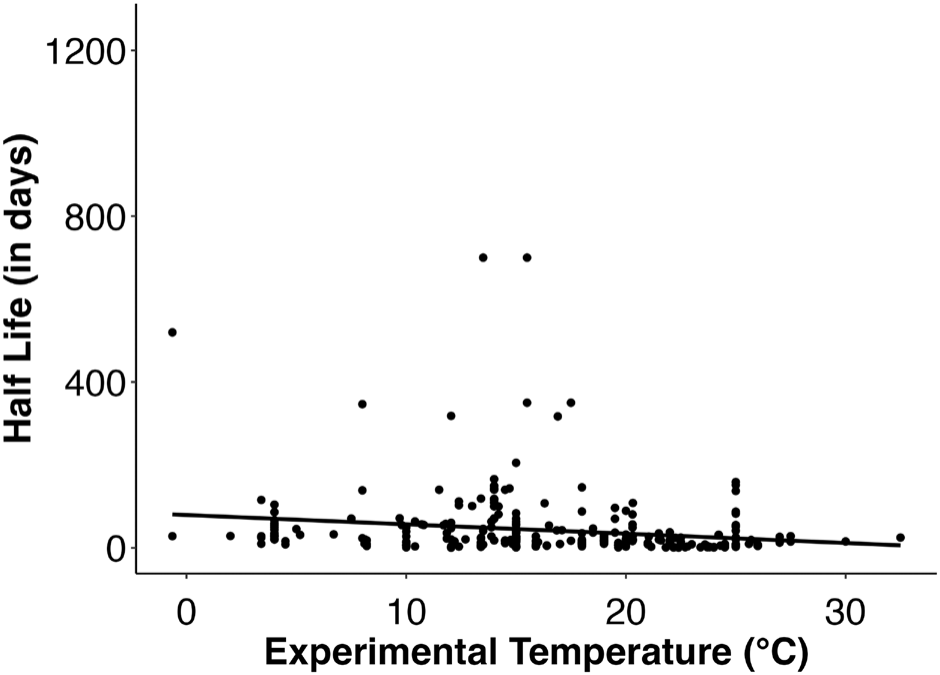
Scatterplot displaying how macroalgal half‐life (in days) varies with experimental temperature (*n* = 319). Macroalgal half‐life significantly decreases with increasing experimental mean temperature; therefore, higher temperatures accelerate macroalgal decay.

Increased temperature caused macroalgal degradation to progress faster, both within and across studies. Eleven studies included in this review independently reported that macroalgal decay quickened at higher temperatures (Table 3). For instance, Boldreel et al. (2023) observed that the decay constant (*k* value) was five times lower at 8°C than at 15°C for *Saccharina latissima* and 9.5 times lower for *Alaria esculenta*. Additionally, Conover et al. (2016) reported a three‐fold increase in the decay constant of *Ulva rigida* with an increase of only 2°C. However, four studies noted that temperature did not play a significant role in macroalgal degradation dynamics (Gao et al., 2021; Kotta et al., 2010; Pedersen et al., 2021; Smale et al., 2022), with Kotta et al. (2010) being the only study to observe that macroalgae decayed significantly faster in cooler water temperatures than warmer water temperatures (only for *Pilayella littoralis*; *Cladophora glomerata* decay was unaffected by temperature). In some cases (Smale et al., 2022), a narrow range of experimental temperatures (9.8–12.4°C) may have limited the detection of a difference in degradation caused by variation in temperature. Across all studies that reported experimental temperature, studies that exposed algae to higher mean temperatures resulted in significantly smaller half‐life values, meaning that macroalgal biomaterial degraded faster in warmer temperatures (*p* < 0.00001; Table S3; Figure 7). More specifically, our model predicted that a 1°C increase in temperature resulted in a 0.078 day decrease in macroalgal half‐life (Table S3). This may mean that ocean warming may lower the carbon sequestration potential of macroalgae (Filbee‐Dexter et al., 2022), although we did not find that temperature significantly influenced percent recalcitrance in our analysis (*p* = 0.3528; Table S4).

**TABLE 3:** Summary of studies which investigated how continuous abiotic variables affect macroalgal degradation dynamics.

Abiotic factor	Positively correlated with degradation	Not correlated with degradation	Negatively correlated with degradation
Temperature	11 studies (Boldreel et al., 2023*; Conover et al., 2016; Dufour et al., 2012; Filbee‐Dexter et al., 2022; Frontier et al., 2022; Hanisak, 1993*; Kristensen et al., 1992; Litchfield et al., 2020; Paalme et al., 2002*; Pedersen & Johnsen, 2017*; Zielinski, 1981*) 15 species tested (9 browns, 3 reds and 3 greens)	4 studies (Gao et al., 2021; Kotta et al., 2010; Pedersen et al., 2021; Smale et al., 2022) 3 species tested (2 browns and 1 green)	1 study (Kotta et al., 2010) 1 species tested (1 brown)
Depth	3 studies (Duggins et al., 2016*; Frontier et al., 2022; Pedersen et al., 2021*) 3 species tested (3 browns)	1 study (Duggins et al., 2016*) 1 species tested (1 brown)	1 study (Salovius & Bonsdorff, 2004) 1 species tested (1 green)
Light	No studies	1 study (Filbee‐Dexter et al., 2022) 2 species tested (2 browns)	2 studies (Frontier et al., 2021, 2022) 2 species tested (2 browns)
Oxygen availability	2 studies (Paalme et al., 2002*; Pedersen et al., 2021) 2 species tested (2 browns)	1 study (Paalme et al., 2002*) 1 species tested (1 brown)	No studies
Salinity	2 studies (Franzitta et al., 2015; Lopes et al., 2011) 1 species tested (1 brown)	2 studies (Conover et al., 2016; Josselyn & Mathieson, 1980*) 2 species tested (1 brown and 1 green)	1 study (Josselyn & Mathieson, 1980*) 1 species tested (1 brown)
pH	No studies	1 study (Litchfield et al., 2020) 1 species tested (1 brown)	No studies
Microplastic concentration	No studies	No studies	1 study (Litchfield et al., 2020) 1 species tested (1 brown)
Water movement	No studies	1 study (Filbee‐Dexter et al., 2022) 2 species tested (2 browns)	No studies
Intertidal shore height	No studies	2 studies (Rodil et al., 2008*; Urban‐Malinga et al., 2008, ^a^) 3 species tested (3 browns)	No studies
Nutrient enrichment	2 studies (Robinson et al., 1982; Yang et al., 2021) 2 species tested (1 brown and 1 green)	No studies	No studies

*Note*: Studies which found that degradation rate was positively correlated, negatively correlated, or not correlated with an increase in the factor listed in that row are pooled. Asterisks denote studies which did not verify statistically whether the factor had a significant effect on degradation rate. Note that some studies reported differing results based on algal species, which is why some studies are listed in multiple columns within the same row.

^a^ This study observed that algal biomaterial degraded fastest at mid‐intertidal shore heights and degraded slowest at the lowest and highest intertidal shore locations.

It has been well established in the terrestrial literature that increased temperatures accelerate organic matter decomposition, since higher temperatures increase the metabolic rate of decomposing organisms (Burke et al., 2004; Conant et al., 2011; Cornwell et al., 2008). Similar review papers on other types of marine macrophyte degradation also identified that higher temperatures increased degradation rates across various studies included in these reviews (mangroves, seagrasses, and tidal marshes in Ouyang et al., 2023; seagrasses in Trevathan‐Tackett et al., 2020). This contrasts with the findings of another review on mangrove degradation in which the authors did not report a significant effect of absolute latitude on degradation rates, which may be due to the narrower range in latitudes and, therefore, temperatures that mangroves grow in (Simpson et al., 2023). It is important to note that in addition to higher temperatures accelerating macroalgal degradation, higher temperatures also often increase the rate at which macroalgae release biomaterials into the water column via erosion and/or DOC release (Bennett et al., 2024; Endo et al., 2020; Krumhansl & Scheibling, 2012b; Pessarrodona et al., 2018). Ocean warming is therefore expected to increase the supply of macroalgal biomaterials to the surrounding environment while also increasing the rate at which those biomaterials degrade.

#### Depth, light, and oxygen availability

The water depth at which macroalgal degradation takes place may impact macroalgal degradation dynamics in situ, since light, temperature, and oxygen availability all typically decline with depth, and microbial and meiofaunal communities can also change with depth (Duggins et al., 2016; Matear & Hirst, 2003; Pawlowicz, 2013; Reynolds & Lutz, 2001; Zhang et al., 2018). Three individual studies included in this review reported that macroalgal degradation accelerated at deeper depths (Duggins et al., 2016; Frontier et al., 2022; Pedersen et al., 2021). One study documented that the effect of depth was dependent on algal species (Duggins et al., 2016) and another study observed that degradation slowed with increasing depth, which the authors attributed to the lower temperatures (~10°C cooler) to which deeper algal detritus was exposed (Salovius & Bonsdorff, 2004).

The acceleration of macroalgal degradation at deeper depths observed in prior studies may be attributed to the decrease in light availability deeper in the water column. This is because detached macroalgal tissues (i.e., detritus) can continue to photosynthesize and grow when there is adequate light (Frontier et al., 2021; Rothäusler et al., 2018; Tala et al., 2019; van Hees et al., 2018; Wright et al., 2024; Wright & Foggo, 2021; Wright & Kregting, 2023). Three studies included in this review assessed the impact of light on macroalgal degradation, and two of the three studies observed that increased light increased the longevity of macroalgal tissues (Table 3; Frontier et al., 2021, 2022). In contrast, Filbee‐Dexter et al. (2022) did not find that light was not a significant predictor of macroalgal degradation in their field study. When we tested directly if light availability significantly impacted macroalgal half‐life or percent recalcitrance across studies (either dark or lit), a significant effect of light was not observed (*p* = 0.4077 for half‐life; *p* = 0.6163 for precent recalcitrance). However, we were not able to include observations that were confounded by growth (i.e., where growth negated degradation) in these analyses, since we were unable to extract half‐life or percent recalcitrance estimates from these data. Interestingly, all observations of macroalgal tissue degradation that were classified as being confounded by growth occurred in experiments wherein adequate light was available: Either experiments were performed at relatively shallow subtidal depths in situ (<10 m) or in a laboratory where artificial light sources were used. It is, therefore, evident that sustained detrital photosynthesis is an important aspect of macroalgal tissue degradation dynamics that should not be overlooked.

Oxygen availability also generally decreases with ocean depth (Lalli & Parsons, 1993). Two individual studies identified that decreased oxygen availability decreased macroalgal degradation rates, although this effect may be species‐dependent (Table 3; Paalme et al., 2002; Pedersen et al., 2021). The slowing of degradation in low or no oxygen conditions is likely due to how restricting oxygen excludes microbes that cannot tolerate hypoxic or anoxic conditions, reducing the diversity and abundance of microbes available to degrade macroalgae (Bertagnolli & Stewart, 2018; Fenchel & Finlay, 2008; Glud, 2008). Trevathan‐Tackett et al. (2020) also documented that increasing oxygen availability increases seagrass degradation rates in their review.

#### Degradation environment

Macroalgal degradation has been investigated in a wide variety of conditions and contexts. Laboratory studies have been equally common as in situ studies. (Forty‐seven percent of studies were laboratory or mesocosm studies, 44% were conducted in situ, and 7% of studies included data from both in situ and laboratory experiments.) Macroalgal degradation can occur intertidally (after macroalgal tissues wash up on shore) or it can occur subtidally (either on the seafloor or while macroalgal biomaterials are suspended in the water column), and authors have investigated macroalgal degradation in all of these contexts (Figure 4c). Nedzarek and Rakusa‐Suszczewski (2004) and Zielinski (1981) both observed that intertidally placed algal biomaterial degraded faster than submerged biomaterial, but Josselyn and Mathieson (1980) documented that continuously submerged detritus degraded faster than detritus exposed to air (Table S6). Perhaps exposure to the air accelerates the senescence process, since this desiccates the tissue (Luo, Xie, et al., 2022). However, our statistical analysis did not indicate that degradation environment had a significant impact on degradation dynamics (Tables S3 and S4). Simpson et al. (2023) also did not find that the placement of litterbags containing mangrove litter (submerged, buried, or exposed to air) had a significant impact on degradation dynamics in their review.

Select studies included in this review observed that macroalgal biomaterial that is in direct contact with the seafloor sediment degrades faster compared to macroalgal biomaterial that is suspended in the water column (Table S6; Salovius & Bonsdorff, 2004; Williams, 1984). This may be attributable to the biomaterial being in closer contact with the microbial and benthic grazer communities in the sediment, although this mechanism has yet to be validated. Furthermore, three studies included in this review investigated how burying macroalgal detritus versus placing detritus on the sediment surface impacts degradation dynamics (Table S6). Boldreel et al. (2023) observed that macroalgae degraded faster on the sediment surface, while Haram et al. (2020) and Kristensen and Mikkelsen (2003) both observed that macroalgae degraded slower when buried in sediment. Haram et al. (2020) and Kristensen and Mikkelsen (2003) both conducted their studies in the presence of grazers, whereas Boldreel et al. (2023) conducted their study in a lab in the absence of grazers. Thus, the presence of grazers is likely an important confounding factor in this context, since burying detritus presumably limits its availability to grazers that inhabit the sediment surface. Relatedly, in the one study directly investigating substratum type (rocky reef compared to soft sediment) on degradation rate, substratum did not impact degradation rate (Hunter, 1976).

#### Litterbag mesh diameter

Eighty‐eight percent of in situ macroalgal tissue degradation studies chose to use mesh bags (litterbags) or mesh cages to contain macroalgal biomaterial in the field, with litterbag mesh size ranging from 53 μm to 5 cm in diameter (median = 1 mm, mean = 4.4 mm; Figure 4d). Litterbag mesh size may influence degradation rate since extremely small, tight mesh can reduce the amount of water and/or sediment in contact with the biomaterial, limiting the exposure of biomaterials to microbes and/or grazers, and reducing light availability (Handa et al., 2014; Lecerf, 2017; Xie, 2020). Larger sized mesh may increase the biomaterial's susceptibility to grazing and microbial attack, since it increases the amount of contact the biomaterial has with the sediment and/or water column, as well as potentially allowing more small/particulate detrital matter to escape through the mesh (Chassain et al., 2021; Harrison, 1989). Hence, the litterbag mesh diameter chosen by a researcher may impact that researchers' subsequent measurements of degradation dynamics.

**FIGURE 8:**
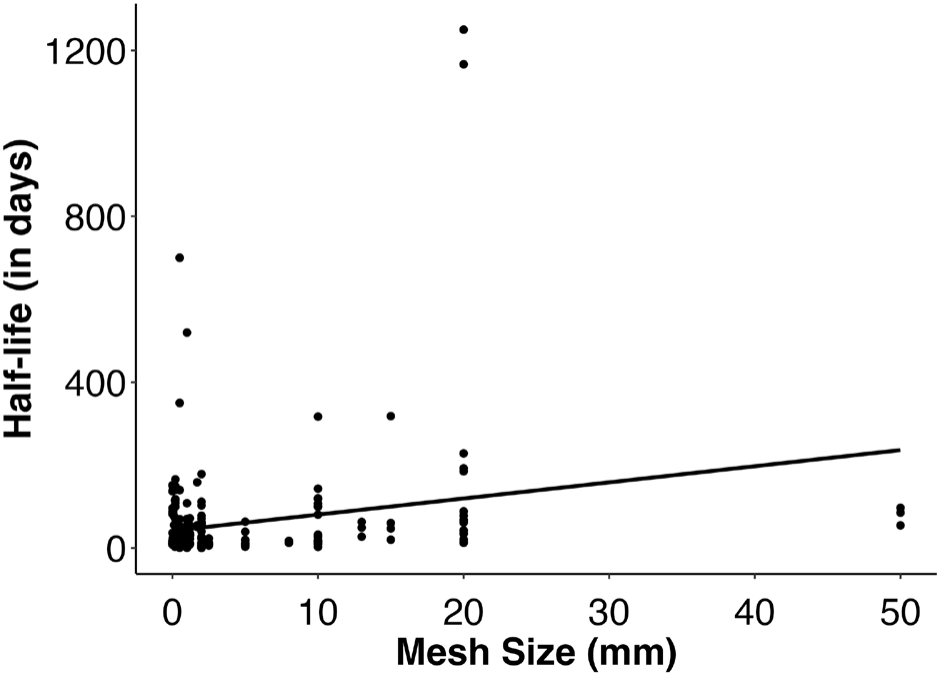
Scatter plot demonstrating the relationship between litterbag mesh size (mm) and macroalgal half‐life (in days); half‐life increases (meaning degradation slows) with increasing mesh size.

Three studies included in this review independently assessed if litterbag mesh size impacted macroalgal degradation dynamics. Two of the studies observed that mesh size had no impact on degradation dynamics (Catenazzi & Donnelly, 2007; Wright & Kregting, 2023), but Bedford and Moore (1984) observed that macroalgae degraded faster in 1‐mm diameter mesh bags, compared to 50‐mm diameter mesh bags (Table S6). Our analysis showed that increasing litterbag mesh diameter leads to slower macroalgal decay (*p* = 0.0411), wherein a 1‐mm increase in mesh size predicts a 0.44 day increase in macroalgal half‐life (Table S3; Figure 8). This may be because larger‐sized mesh allows more light to reach macroalgal detritus, which allows detrital tissue to continue to photosynthesize and avoid degradation (Frontier et al., 2021). However, litterbag mesh diameter was not a significant predictor of macroalgal percent recalcitrance (*p* = 0.0776). Contrastingly, a systematic review of terrestrial organic matter degradation studies observed that increasing litterbag mesh size significantly accelerates terrestrial leaf litter degradation, as evidenced by higher *k*‐values (Xie, 2020).

#### Experiment duration

Most studies included in this review had relatively short durations (mean = 54 days, median = 35 days; Figure 4e). Short experiment durations lower the confidence with which we can incorporate macroalgal degradation data into predictive models, which could estimate the ultimate fate of macroalgal biomaterials. This is because there is wide variation in coastal residence time (i.e., time it takes for coastal waters to mix with the open ocean), ranging from just a few days to weeks, sometimes up to years (Filbee‐Dexter et al., 2024; Liu et al., 2019; Safak et al., 2015), so understanding the long‐term degradation dynamics of macroalgae is crucial in the context of carbon sequestration. Furthermore, experiment duration may impact our perception of degradation dynamics since short experiments may only capture the initial stages of degradation, excluding the posterior stages of degradation which are typically characterized by slower degradation rates (Figure S2). Longer experiment durations also enable researchers to make more accurate estimates of how much macroalgal biomaterial is recalcitrant. However, our analysis did not uncover a significant effect of experiment duration on macroalgal percent recalcitrance (*p* = 0.3993; Table S4; half‐life data could not be analyzed). Trevathan‐Tackett et al. (2020) noted in their review that studies on seagrass degradation with durations of <120 days had a two‐fold increase in average half‐life, compared to studies >120 days in duration. Our macroalgal data show a similar, albeit much less pronounced trend, wherein studies that were longer than 120 days reported a mean half‐life of 61.40 ± 5.79 days, whereas studies shorter than 120 days reported a shorter mean half‐life of 50.34 ± 18.9 days.

#### Pre‐treatment of macroalgae

**FIGURE 9:**
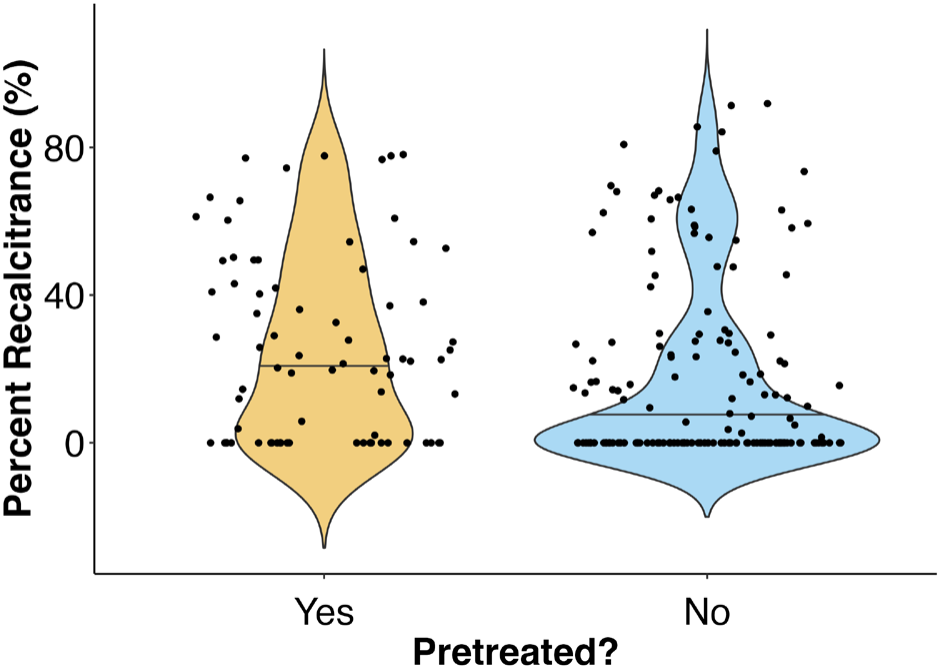
Violin plot displaying how macroalgae which had been pre‐treated have higher percent recalcitrance (%) as compared to macroalgae which were not pre‐treated.

Almost half of all studies pre‐treated macroalgal biomaterial before experimentation began (42%), commonly by oven‐drying or freeze‐drying macroalgae (Josselyn & Mathieson, 1980; Luo, Dai, et al., 2022; Smith & Foreman, 1984). This is often done with the justification of ensuring that the macroalgae is dead, as well as minimizing morphological differences between species for cross‐species comparisons of degradation rates (Kim et al., 2021; Lopes et al., 2011). It also enables researchers to record initial dry weight measurements of macroalgal tissue. Eight studies included in this review independently assessed the impact of pre‐treating macroalgae on subsequent macroalgal degradation dynamics, and more than half (57%) observed that degradation quickened after macroalgae were pre‐treated, compared to un‐treated macroalgae, whereas 28% documented a negligible effect (Table S6). Our analysis showed that macroalgal half‐life was not significantly impacted by pre‐treatment (*p* = 0.3549). Interestingly, our analysis did find that macroalgae that were pre‐treated had significantly higher proportions of recalcitrant biomaterials compared to macroalgae that were not pre‐treated (*p* = 0.00691; Figure 9). The mechanism behind this difference is unknown, although it is possible that different pre‐treatment methods (including oven drying, air drying, freezing, and others) impact the stability of recalcitrant biomaterials, ultimately altering degradation dynamics.

### Comparison to other marine macrophytes

Many studies included in this review compared tissue degradation dynamics between vascular marine plants and macroalgae, and all of these studies observed that macroalgae degraded faster than seagrasses (Bourguès et al., 1996; Buchsbaum et al., 1991; Gladstone‐Gallagher et al., 2016; Josselyn & Mathieson, 1980; Kristensen, 1994; Lanari, Claudino, et al., 2018; Liu et al., 2020; Lopes et al., 2011; Rice, 1982; Rice & Tenore, 1981; Thomson et al., 2020; Twilley et al., 1986; Vichkovitten & Holmer, 2004) and faster than mangroves (Gladstone‐Gallagher et al., 2016; Kristensen, 1994; Rice, 1982; Rice & Tenore, 1981). Buchsbaum et al. (1991) observed that phenolic content decreased at a faster rate in the algae *Fucus vesiculosis* than in seagrasses throughout degradation time, which might explain this trend. In other words, macroalgal phenolic compounds may be more susceptible to degradation as compared to seagrass phenolic compounds. Also, seagrasses and mangroves contain particularly recalcitrant cell structural compounds such as lignocellulose, which are especially hard for microbes to degrade and are absent from macroalgae (Benner & Hodson, 1985; Klap et al., 2000).

**FIGURE 10:**
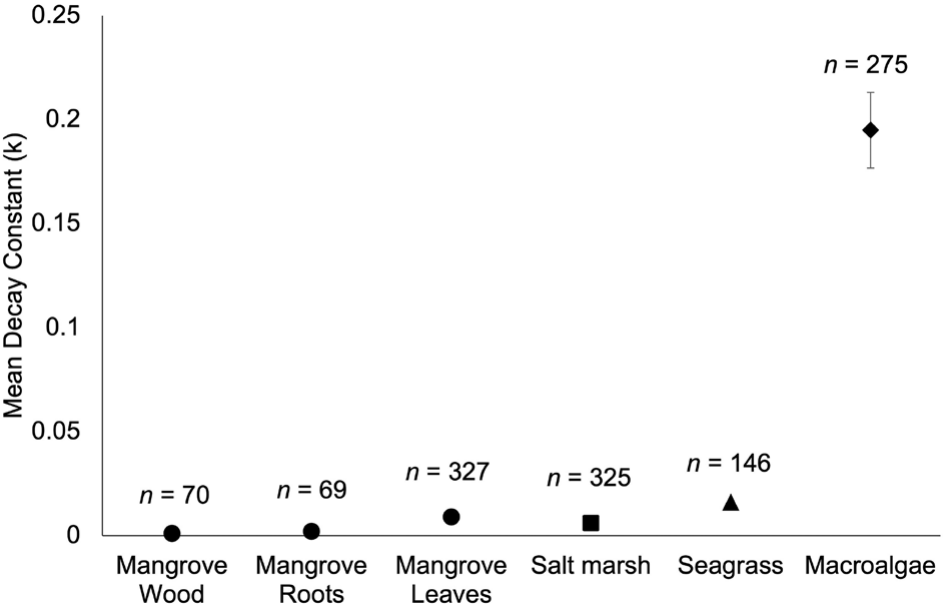
Comparison of mean decay constant values reported by similar systematic reviews of marine macrophyte degradation. Mangrove litter mean *k* values are extracted from (Simpson et al., 2023), salt marsh and seagrass litter mean *k* values are derived from (Ouyang et al., 2023), and macroalgae mean *k* value is taken from this review. Error bars are standard error; sample sizes are included above each point.

Furthermore, after comparing the results of our study to those of similar review papers on marine macrophyte degradation, macroalgae decay faster than other forms of marine macrophyte detritus, on average, as evidenced both by a faster mean degradation rate and a larger mean decay constant (Lovelock et al., 2017; Ouyang et al., 2017, 2023; Simpson et al., 2023; Table S7; Figure 10). Ouyang et al. (2023) compiled litter degradation data across different types of coastal vegetated ecosystems in their review and observed that macroalgae decay was, on average, the fastest, as evidenced by a higher mean decay constant (*k*). The mean *k* value for macroalgae was highest at 20 ± 2 × 10^−2^ · day^−1^, while the mean *k* value for mangroves, seagrasses, and tidal marsh plants were 1.5 ± 0.1 × 10^−2^ · day^−1^, 1.6 ± 0.1 × 10^−2^ · day^−1^, and 6.0 ± 0.5 × 10^−3^ · day^−1^, respectively (Ouyang et al., 2023).

### Macroalgal tissue composition

Carbon and nitrogen content, as well as carbon:nitrogen (C:N) ratios, were often measured in macroalgal detritus degradation studies, as this gives insight into the quality of macroalgae as a food source, as well as the rate that these two key nutrients are released to the ecosystem throughout degradation (Norderhaug et al., 2006). Thirty‐seven percent of studies included in this review reported how carbon and nitrogen content changed during degradation. We had predicted that higher initial nitrogen content and lower initial carbon content would predict faster degradation, since high nitrogen content in primary producer tissues is thought to be more palatable to microbes and grazers who are often nitrogen limited (Enríquez et al., 1993; Sterner & Hessen, 1994). Filbee‐Dexter et al. (2022) and Wright et al. (2022) both observed that higher initial carbon content in macroalgal tissues predicted slower degradation, although in both cases initial carbon content only partially explained variations in macroalgal degradation across sites and/or species. When we tested whether initial carbon content, initial nitrogen content, or initial carbon:nitrogen ratios significantly predicted macroalgal biomaterial half‐life using a LMM with study as a random effect, there were no significant effects (*p* = 0.66 for C:N; *p* = 0.82 for carbon; *p* = 0.43 for nitrogen; Table S8).

Across marine, aquatic, and terrestrial plants, C:N ratios typically decline with degradation time (Enríquez et al., 1993). Many studies on macroalgal degradation have reported that macroalgal detritus becomes enriched in nitrogen as decomposition progresses, thus reducing the C:N ratio (Birch et al., 1983; Brouwer, 1996; Hwang et al., 2023; Krumhansl & Scheibling, 2012a; Norderhaug et al., 2006; Smith & Foreman, 1984; Twilley et al., 1986). Nitrogen enrichment of detritus is usually attributed to the microbial transformation of detritus throughout degradation (Norderhaug et al., 2003; Tenore et al., 1979), although some macroalgal degradation studies have reported an increase in the C:N ratio throughout degradation (Pedersen et al., 2021; Urban‐Malinga et al., 2008; Vichkovitten & Holmer, 2004). Across all studies included in this review that measured C:N, 64% reported a decrease of greater than 10% in C:N, whereas 16% reported an increase in C:N of more than 10%. The average percent change in C:N across the entire degradation experiment is −21.37% (*N* = 140), which may mean that macroalgae become less palatable to consumers throughout degradation time since grazers prefer C:N ratios that are not too high nor too low (Norderhaug et al., 2006).

Brown algae synthesize polyphenolics (including phlorotannins), which are thought to protect their tissues from UV damage, microbial attack, and grazing (Duggins & Eckman, 1997; Geiselman & McConnell, 1981; Paul et al., 2006). Thus, many researchers have been interested in whether polyphenolic content has a predictive or explanatory role in brown algal degradation. Wright et al. (2022) observed that higher polyphenolic content predicted slower decomposition rates both across various Laminarian species as well as between specific individuals of the same species. Similarly, Pedersen et al. (2021) observed that kelp stipes contained higher portions of polyphenolics and decayed slower than kelp blades, which had lower polyphenolic concentrations. However, Gilson et al. (2021) observed that polyphenolic content did not predict differences in degradation rates between kelp species. Therefore, while measuring polyphenolic content can give some insight into brown algal degradation dynamics, polyphenolic concentrations do not fully explain brown algal degradation dynamics. This may be because there are many forms of polyphenolics, and some forms are more labile than others (Paul et al., 2006). However, polyphenolics may leach from the algae during degradation, removing their protective capacity during the later stages of degradation (Norderhaug et al., 2006; Pedersen et al., 2021). In summary, although C:N ratio typically declines as macroalgal degradation progresses, reducing the palatability of macroalgal detritus, the decline in polyphenolics during brown algal degradation may make detritus less chemically defended and therefore more accessible to consumers as degradation progresses.

### Impact of microbial community on degradation

The microbial community is very likely an important driver of macroalgal degradation, as many studies have shown that degradation progresses faster when microbes are present or present in higher concentrations (Li, Feng, Xiong, He, et al., 2023; Li, Feng, Xiong, Shao, et al., 2023; Lucas et al., 1981; Perkins et al., 2023; Stuart et al., 1981). Moreover, the primary potential mechanism behind the strong impact of temperature on organic matter degradation is that temperature accelerates microbial metabolisms (Burke et al., 2004; Cornwell et al., 2008). Also, the high variability in macroalgal degradation dynamics across studies may be partially attributable to variations in microbial communities caused by certain experimental choices that have been taken in laboratory studies (including whether natural or artificial seawater was used, whether microbes were artificially enriched/spiked, or whether natural sediment was included in experimental tanks; Huang et al., 2023; Li et al., 2022; Rieper‐Kirchner, 1989).

Previous studies have investigated how the microbial community changes throughout macroalgal degradation. Bacterial abundance usually increases during the early stages of degradation, and then begins to gradually decline, presumably since the amount and accessibility of biomaterial decrease with time (Chen et al., 2020; Lucas et al., 1981; Manikandan et al., 2021; Rieper‐Kirchner, 1989). Bacteria from the phylum Proteobacteria typically dominate the bacterial community in the preliminary stages of degradation, then the bacterial community generally diversifies as degradation progresses (Brunet et al., 2021; Chen et al., 2020; Feng et al., 2022; Hu et al., 2023; Ihua et al., 2019; Liang et al., 2021; Lozada et al., 2023; Xie et al., 2024; Zhang et al., 2024; Zhang, Qin, et al., 2022). Across these studies, algal‐degrading bacterial communities vary substantially with environment, location, oxygen availability, and algal species. Common algal‐degrading bacteria that increase in prominence in later stages of degradation include bacteria from the genera *Flavobacterium* (known for their fucoidan‐degrading capacity; Brunet et al., 2021; Liang et al., 2021; Lozada et al., 2023; Sakai et al., 2002) as well as bacteria from the phylum Planctomycetes (known for their ability to degrade sulphated polysaccharides; Hu et al., 2023; Lage & Bondoso, 2014; Lozada et al., 2023; Wegner et al., 2013; Xie et al., 2024). Also, Perkins et al. (2023) noted that fungi play an important role in kelp degradation, since after the addition of fungicide, kelp degradation progressed significantly slower.

### Key knowledge gaps

It remains unclear how persistent macroalgal‐derived carbon is within the ocean, preventing macroalgae from being included in current blue carbon budgets (Pessarrodona et al., 2023). One key area of uncertainty that remains is the understanding of macroalgal‐derived exudate (DOC, POC, and POM) degradation dynamics. There is reason to suspect that macroalgal‐derived DOC persisting in the ocean may be a key pathway for macroalgal carbon sequestration (Pessarrodona et al., 2023); there is a vast oceanic DOC pool that has remained relatively stable throughout time (Baltar et al., 2021; Cai & Jiao, 2023), and macroalgae are one of the main sources of DOC that contribute to coastal DOC pools (Lønborg et al., 2020; Wada & Hama, 2013). Additionally, although the effect of temperature on macroalgal degradation is quite well established, the effects of other abiotic factors like depth, oxygen concentration, substratum type, salinity, and pH on degradation have been variable across contexts and need further study (Table 3 and Table S6). This is particularly important considering that we may expect these abiotic factors to change as climate change progresses, necessitating more study on how these factors impact macroalgal degradation dynamics.

Macroalgae represent a highly diverse, polyphyletic group, in contrast with other vegetated blue carbon ecosystems that are relatively more homogeneous (mangroves, seagrasses, and tidal marshes; Krause‐Jensen et al., 2018). This diversity likely causes variations in degradation trajectories, since different macroalgal taxonomic and functional groups have distinct cell wall compounds (Lee & Ho, 2022; Popper et al., 2011; Synytsya et al., 2015). This diversity may explain why macroalgae *k*‐values are more variable across the literature when compared with other more uniform types of marine macrophytes (Figure 10). Our analysis showed that brown algae are more recalcitrant when compared to red algae (with no significant difference when compared to green algae). This finding should be interpreted with caution, however, due to the over‐representation of brown algae in the data set (and corresponding under‐representation of other algal groups), highlighting the need for more study on other algal groups. Indeed, only 99 species of macroalgae have been assessed for their degradation dynamics (for a full list of species see Supplemental Datafile S6), which is only a tiny fraction of the thousands of species of macroalgae currently recognized (Guiry, 2024). Thus, many species of macroalgae remain unstudied, limiting our ability to make well‐founded conclusions about trends in degradation across all macroalgae. Particularly, due to the loss of kelps and fucoids in many ecosystems, species of turfing algae and crustose coralline algae are becoming increasingly dominant (Filbee‐Dexter & Wernberg, 2018; Matsunaga et al., 1999; Rogers‐Bennett & Catton, 2019; Smith et al., 2024), so understanding how these groups of algae contribute to coastal carbon cycling is paramount. Additionally, coralline algae are very thermally stable (Trevathan‐Tackett et al., 2015), meaning that they may be important contributors to carbon sequestration; however, whether the carbon dioxide produced by coralline algae during CaCO_3_ production exceeds their contributions to carbon sequestration is contested (Smeaton et al., 2017; van der Heijden & Kamenos, 2015).

### Recommended methods

In future studies, striving to fit models that accurately explain macroalgal degradation data is imperative in order to understand the longevity of macroalgal biomaterials in the ocean (Lian et al., 2023; Pessarrodona et al., 2023). This review has shown that macroalgal degradation is not always best explained by exponential decay models, despite the common assumption that all organic matter degrades following an exponential decay curve (Berg & McClaugherty, 2003; Wider & Lang, 1982). Crucially, carefully fit models will enable us to accurately assess if there is recalcitrant matter, which will lead to a better understanding of the carbon sequestration potential of macroalgae (Hurd et al., 2022; Pessarrodona et al., 2023). In this vein, increasing the experimental duration of degradation experiments to at least 1 month, preferably longer, would enable more precise model fitting and estimation of recalcitrance while also reducing the risk of overfitting models. It is also important to sample decaying macroalgal biomaterials frequently throughout their degradation times, as this improves our ability to fit models to degradation trajectories; at least four to five datapoints is ideal for subsequent model fitting. Currently, 25% of studies included in this review made only one final assessment of degradation (plus an initial assessment), which severely limited our ability to understand degradation trajectory and estimate percent recalcitrance (Figure 4f).

Pre‐treating macroalgae prior to experimentation may limit the confidence with which studies' findings can be used to understand how algae decay in situ, particularly since we determined that pre‐treating algae significantly influences macroalgal percent recalcitrance. Thus, we do not recommend pre‐treating macroalgae prior to degradation if one's goal is to understand the carbon sequestration potential of macroalgae better. Likewise, since litterbag mesh size impacts macroalgal degradation dynamics, size should be considered carefully to enhance repeatability and ecological relevance. The median mesh diameter used by studies included in this review was 1 mm, so moving forward, using 1‐mm diameter mesh in macroalgal degradation litterbag studies is a reasonable choice.

Lastly, in the endeavor to estimate the carbon sequestration potential of macroalgae, one key aspect future researchers should focus on is the relative recalcitrance of macroalgal organic matter. There are many alternate approaches researchers can use to assess the relative recalcitrance of organic matter, which include thermogravimetric analyses of tissue stability (Trevathan‐Tackett et al., 2015), quantifying the amount of already known recalcitrant compounds in tissues (which can be done through a vast variety of methods, reviewed in Cai & Jiao, 2023), or taking marine sediment core samples to categorize the age and source of carbon contained within them (Hansen et al., 2022; Ørberg et al., 2023; Queirós et al., 2023). The method used in this review, fitting models to degradation data and determining at what point the degradation rate approaches zero, is beneficial, as it can give us an idea of recalcitrance by making use of already collected data. However, it is important to note that an inherent issue with the common in situ litterbag/mesh bag approach is that these studies cannot track the fate of the biomaterial (whether it be tissue, POM, POC, or DOC) that escapes through the holes in the mesh. Conducting more studies that measure the longevity of macroalgal POM, POC, and DOC will enable a better understanding of the recalcitrance of all types of macroalgal biomaterials. However, recalcitrance is not a straightforward concept, as it can depend on a myriad of factors (Jiao et al., 2014; Schmidt et al., 2011; Shen & Benner, 2018). Therefore, more work is necessary to determine the mechanisms that confer recalcitrance in macroalgae, including the relative importance of molecular stability, abiotic factors, and microbial community.

## CONCLUSIONS

Macroalgal degradation is a complex process with a variety of important implications for ecosystem functioning and marine carbon cycling (Lastra et al., 2008; Ouyang et al., 2023; Renaud et al., 2015; Watanabe et al., 2020). By summarizing the current literature on the topic of macroalgal degradation, we aimed to shed light on this important topic and highlight future directions of research. One of our main findings was that there is substantial variation in macroalgal decay trajectories, meaning that authors should select and compare appropriate macroalgal decay curves in the future to garner a more precise understanding of macroalgal degradation and relative recalcitrance (i.e., longevity). Next, we identified a variety of factors that impact degradation dynamics in macroalgae: Increasing temperature accelerates macroalgal degradation; larger litterbag mesh sizes slow macroalgal degradation; brown algae are more recalcitrant than reds; and pre‐treating macroalgae prior to experimentation impacts estimates of percent recalcitrance. The impacts of other drivers on macroalgal degradation (including biomaterial type, functional group, degradation environment, and experiment duration) vary across contexts and, therefore, ought to be investigated further. In addition, we highlighted that detrital photosynthesis is an important aspect of macroalgal degradation, that macroalgal tissue composition (including carbon content and polyphenolic content) does not fully explain variations in macroalgal degradation dynamics, and that the microbial community is a major driver of macroalgal degradation. Lastly, we emphasized that many knowledge gaps persist, such as the lack of geographic and taxonomic diversity in the literature and the lack of study on macroalgal DOC and POM/POC degradation.

The extent of the contribution of macroalgae to marine carbon sequestration remains uncertain due to the complexities involved in tracking macroalgal‐derived carbon from its source to a sink where carbon sequestration is possible (i.e., soft sediments or the deep sea; Dolliver & O'Connor, 2022a; Fujita et al., 2023; Krause‐Jensen et al., 2018). Garnering a better understanding of macroalgal degradation will advance our understanding of macroalgal carbon sequestration because the longevity of macroalgal biomaterials influences the likelihood that those biomaterials reach habitats where carbon sequestration is probable (Filbee‐Dexter et al., 2024; Pessarrodona et al., 2023; Queirós et al., 2023). In the case of macroalgal‐derived DOC, we lack evidence that a substantial amount of macroalgal DOC can persist in the ocean for 100 years or more (Paine et al., 2021). Therefore, understanding the long‐term persistence of macroalgal DOC will greatly expand our capacity to quantify the contribution of macroalgae to marine carbon sequestration (Cai & Jiao, 2023; Lønborg et al., 2020). Thus, further targeted study of the degradation dynamics and recalcitrance of a broad range of macroalgal biomaterials under a broad range of conditions will improve our understanding of the carbon sequestration capacity of macroalgae in addition to furthering our knowledge of the role of macroalgal biomaterials as trophic subsidies and as crucial habitat and food sources for meiofaunal communities.

## GLOSSARY

*Blue carbon*: carbon which is sequestered by marine ecosystems, examples of blue carbon ecosystems include mangroves, seagrass meadows and salt marshes.

*Carbon sequestration*: the capture and storage of carbon in an inert form, usually for at least 100 years.

*Detritus*: here we use this term to refer to macroscopic tissue debris released from macroalgae which could range from small tissue fragments to an entire, dislodged algae (alternate terms: wrack or litter).

*Degradation*: the transformation of organic molecules contained within organic matter into inorganic molecules, typically done by microorganisms. This process results in the release of nutrients to the system, as well as the release of carbon dioxide (alternate terms: decomposition or decay).

*Dissolved organic carbon (DOC)*: organic carbon that is dissolved in a body of water, typically referring to carbon that can pass through either a 0.2‐ or 0.7‐μm filter.

*Dissolved organic matter (DOM)*: refers to all fractions of dissolved organic matter in a body of water that can pass through a either a 0.2‐ or 0.7‐μm filter. Note that all studies included in this review chose to analyze change in macroalgal‐derived DOC, rather than DOM.

*Exudates*: defined here as macroalgal‐derived DOC, DOM, POM, and POC inclusively.

*Foliose algae*: defined here as macroalgae larger than turf and not within the kelp or fucoid functional groups.

*Fucoid*: brown algae from the order Fucales.

*Kelp*: brown algae from the order Laminariales.

*Labile*: organic matter which is susceptible to degradation.

*Macroalgal biomaterial*: we use this term throughout to refer to any organic matter derived from macroalgae that is released into the ocean. This includes the macroalgal exudates—DOM (including DOC), POM (including POC)—as well as detritus (macroalgal tissue fragments and whole detached plants).

*Meiofauna*: small invertebrates which live in benthic marine and/or freshwater environments that are smaller than macrofauna but larger than microfauna.

*Particulate organic carbon (POC)*: particulate organic carbon from a freshwater or marine source. The operational definition of particulate organic matter/carbon varies substantially across the body of literature included in this review. Some authors define particulate matter/carbon as suspended particles that are caught on a filter (either 0.2 or 0.7 μm) or they define particulate matter/carbon as being smaller than a certain threshold size, ranging between 10 and 750 μm.

*Particulate organic matter (POM)*: refers to all fractions of particulate organic matter from a freshwater or marine source. The operational definition of particulate matter varies across the literature included in this review, see above.

*Recalcitrant*: organic material which persists in the environment, avoiding degradation or ingestion (alternate term: refractory). There is no standard length of time for which organic matter must resist degradation for it to be deemed recalcitrant. In this paper, we designated that there is a portion of recalcitrant biomaterial if the degradation trajectory had a positive horizontal asymptote *and* the experiment lasted longer than 1 month.

*Turf algae*: low lying, dense aggregations of foliose or filamentous algae of various taxonomic groups.

## AUTHOR CONTRIBUTIONS

**Jessica R. Kennedy:** Data curation (equal); formal analysis (lead); investigation (equal); methodology (equal); visualization (lead); writing – original draft (lead); writing – review and editing (equal). **Caitlin O. Blain:** Conceptualization (lead); data curation (equal); funding acquisition (lead); investigation (equal); methodology (equal); supervision (lead); writing – review and editing (equal).

## Supporting information


**Figure S1.** PRISMA (Preferred Reporting Items for Systematic Reviews and Meta‐Analyses) flow diagram for this systematic review.
**Figure S2.** Example degradation trajectory that exhibits a three‐parameter exponential decay relationship (black circles). If the experimental duration is confined to the initial decay period (e.g., red triangles), the degradation trajectory may appear linear, and recalcitrance is challenging to estimate. Longer experiment durations are beneficial as it enables us to understand the longevity of macroalgal biomaterial.
**Figure S3.** Violin plots demonstrating how macroalgal half‐life (log_10_ transformed) varies across macroalgal functional groups (a) and macroalgal classification (b). Neither class nor functional group was a significant predictor of half‐life.
**Table S1.** Details of the systematic search conducted by the authors including details on who conducted searches and when and the exact search terms used for each database.
**Table S2.** Global mean values for all macroalgal degradation studies included in this review. Decay constant values were only extracted from models that were best described by exponential decay curves and degradation rate values were only estimated from data described by linear models. Data are presented as mean ± standard error, sample size refers to the number of individual observations.
**Table S3.** To analyze the impacts of various factors on macroalgal half‐life we used a multifactor linear mixed effects model, with study included as a random effect. The model formula was: half‐life ~ functional group + class + experimental temperature + degradation environment + litterbag mesh size + light availability + pretreatment + (1|reference). Both half‐life and litterbag mesh size data were log‐transformed. The final row refers to the significance of the random effect of study.
**Table S4.** To analyze the impacts of various factors on macroalgal recalcitrance (%) we used a multifactor generalized linear mixed effects model with a zero‐inflated negative binomial distribution, and study included as a random effect. The model formula was: percent recalcitrance (%) ~ functional group + class + experimental temperature + experiment duration + degradation environment + litterbag mesh size + light availability + pretreatment + (1|reference). Litterbag mesh size data were log‐transformed. The final row refers to the significance of the random effect of study.
**Table S5.** Findings of studies which compared how algae from different functional groups degrade. In the half‐life column, species are listed in order of shortest to longest half‐life, in days. In the recalcitrance column, species are listed in order of smallest to biggest portion of percent recalcitrant material. Comparisons are based on this review's independent estimates of half‐life and percent recalcitrance. Turf algal species are in green text, kelps are brown, foliose are black, and fucoids are blue. Asterisks denote studies which did not test if the difference in degradation dynamics were statistically significant.
**Table S6.** A summary of the findings of prior studies which tested how a variety of discrete methodological and environmental factors impact macroalgal degradation dynamics. Asterisks denote studies which did not verify statistically whether the factor effected degradation.
**Table S7.** Comparison of the results of our review and the findings other comprehensive reviews on marine biomaterial decomposition. Data are presented as mean ± standard error *or* as mean (5th percentile‐95th percentile) *or* as just the mean value. N.S. stands for not specified.
**Table S8.** Linear mixed effects model analyzing the effect of carbon and nitrogen content on macroalgal half‐life. Formula: Half‐life ~ Initial C:N ratio + Initial percent carbon + Initial percent nitrogen + (1|Study).

**Appendix S1.** Systematic review reference list.

## Data Availability

All datas (Supplemental Datafiles 1–6) have been uploaded to the following link and are publicly accessible for viewing and downloading: https://osf.io/vmtfd/files/osfstorage; DOI: 10.17605/OSF.IO/VMTFD

## References

[jpy70031-bib-0001] Adair, E. C. , Parton, W. J. , Del Grosso, S. J. , Silver, W. L. , Harmon, M. E. , Hall, S. A. , Burke, I. C. , & Hart, S. C. (2008). Simple three‐pool model accurately describes patterns of long‐term litter decomposition in diverse climates. Global Change Biology, 14(11), 2636–2660. 10.1111/j.1365-2486.2008.01674.x

[jpy70031-bib-0002] Ager, T. G. , Krause‐Jensen, D. , Olesen, B. , Carlson, D. F. , Winding, M. H. S. , & Sejr, M. K. (2023). Macroalgal habitats support a sustained flux of floating biomass but limited carbon export beyond a Greenland fjord. Science of the Total Environment, 872, 162224. 10.1016/j.scitotenv.2023.162224 36804986

[jpy70031-bib-0003] Alderkamp, A.‐C. , Van Rijssel, M. , & Bolhuis, H. (2007). Characterization of marine bacteria and the activity of their enzyme systems involved in degradation of the algal storage glucan laminarin. FEMS Microbiology Ecology, 59(1), 108–117. 10.1111/j.1574-6941.2006.00219.x 17233748

[jpy70031-bib-0004] Alkemade, R. , & Van Rijswijk, P. (1993). Path analyses of the influence of substrate composition on nematode numbers and on decomposition of stranded seaweed at an Antarctic coast. Netherlands Journal of Sea Research, 31(1), 63–70. 10.1016/0077-7579(93)90018-N

[jpy70031-bib-0005] Amelung, W. , Brodowski, S. , Sandhage‐Hofmann, A. , & Bol, R. (2008). Combining biomarker with stable isotope analyses for assessing the transformation and turnover of soil organic matter. In Advances in agronomy (Vol. 100, pp. 155–250). Academic Press. 10.1016/S0065-2113(08)00606-8

[jpy70031-bib-0006] Arina, N. , Hidayah, N. , Hazrin‐Chong, N. H. , & Rozaimi, M. (2023). Algal contribution to organic carbon sequestration and its signatures in a tropical seagrass meadow. Deep Sea Research Part II: Topical Studies in Oceanography, 210, 105307. 10.1016/j.dsr2.2023.105307

[jpy70031-bib-0007] Armstrong, S. M. , & Patel, T. R. (1994). Microbial degradation of phloroglucinol and other polyphenolic compounds. Journal of Basic Microbiology, 34(2), 123–135. 10.1002/jobm.3620340208 8014845

[jpy70031-bib-0008] Arnosti, C. (2011). Microbial extracellular enzymes and the marine carbon cycle. Annual Review of Marine Science, 3, 401–425. 10.1146/annurev-marine-120709-142731 21329211

[jpy70031-bib-0009] Atwood, T. B. , Witt, A. , Mayorga, J. , Hammill, E. , & Sala, E. (2020). Global patterns in marine sediment carbon stocks. Frontiers in Marine Science, 7, 165. 10.3389/fmars.2020.00165

[jpy70031-bib-0010] Baker, C. A. , Martin, A. P. , Yool, A. , & Popova, E. (2022). Biological carbon pump sequestration efficiency in the North Atlantic: A leaky or a long‐term sink? Global Biogeochemical Cycles, 36(6), e2021GB007286. 10.1029/2021GB007286

[jpy70031-bib-0011] Baltar, F. , Alvarez‐Salgado, X. A. , Arístegui, J. , Benner, R. , Hansell, D. A. , Herndl, G. J. , & Lønborg, C. (2021). What is refractory organic matter in the ocean? Frontiers in Marine Science, 8, 247–253. 10.3389/fmars.2021.642637

[jpy70031-bib-0012] Barrón, C. , Apostolaki, E. T. , & Duarte, C. M. (2014). Dissolved organic carbon fluxes by seagrass meadows and macroalgal beds. Frontiers in Marine Science, 1, 42. 10.3389/fmars.2014.00042

[jpy70031-bib-0013] Barrón, C. , & Duarte, C. M. (2015). Dissolved organic carbon pools and export from the coastal ocean. Global Biogeochemical Cycles, 29, 1725–1738. 10.1002/2014GB005056

[jpy70031-bib-0014] Bates, D. , Mächler, M. , Bolker, B. , & Walker, S. (2015). Fitting linear mixed‐effects models using lme4. Journal of Statistical Software, 67(1), 1–48. 10.18637/jss.v067.i01

[jpy70031-bib-0015] Bauer, J. E. , & Druffel, E. R. M. (1998). Ocean margins as a significant source of organic matter to the deep open ocean. Nature, 392(6675), 482–485. 10.1038/33122

[jpy70031-bib-0016] Becker, S. , Tebben, J. , Coffinet, S. , Wiltshire, K. , Iversen, M. H. , Harder, T. , Hinrichs, K.‐U. , & Hehemann, J.‐H. (2020). Laminarin is a major molecule in the marine carbon cycle. Proceedings of the National Academy of Sciences, 117(12), 6599–6607. 10.1073/pnas.1917001117 PMC7104365 32170018

[jpy70031-bib-0017] Bedford, A. P. , & Moore, P. G. (1984). Macrofaunal involvement in the sublittoral decay of kelp debris: The detritivore community and species interactions. Estuarine, Coastal and Shelf Science, 18(1), 97–111. 10.1016/0272-7714(84)90009-X

[jpy70031-bib-0018] Beng, K. C. , & Corlett, R. T. (2020). Applications of environmental DNA (eDNA) in ecology and conservation: Opportunities, challenges and prospects. Biodiversity and Conservation, 29(7), 2089–2121. 10.1007/s10531-020-01980-0

[jpy70031-bib-0019] Benner, R. , & Hodson, R. (1985). Microbial degradation of the leachable and lignocellulosic components of leaves and wood from *Rhizophora mangle* in a tropical mangrove swamp. Marine Ecology Progress Series, 23, 221–230. 10.3354/meps023221

[jpy70031-bib-0020] Bennett, E. , Paine, E. R. , Britton, D. , Schwoerbel, J. , & Hurd, C. L. (2024). The effect of temperature on rates of dissolved organic carbon (DOC) release by the kelp *Ecklonia radiata* (phylum Ochrophyta): Implications for the future coastal ocean carbon cycle. Journal of Phycology, 60(6), 1471–1484. 10.1111/jpy.13518 39660554

[jpy70031-bib-0021] Berg, B. , & Laskowski, R. (2005). Methods in studies of organic matter decay. In Advances in ecological research (Vol. 38, pp. 291–331). Elsevier. 10.1016/S0065-2504(05)38009-3

[jpy70031-bib-0022] Berg, B. , & McClaugherty, C. (2003). Plant litter: Decomposition, humus formation, carbon sequestration. Springer Verlag.

[jpy70031-bib-0023] Bertagnolli, A. D. , & Stewart, F. J. (2018). Microbial niches in marine oxygen minimum zones. Nature Reviews Microbiology, 16(12), 723–729. 10.1038/s41579-018-0087-z 30250271

[jpy70031-bib-0024] Birch, P. B. , Gabrielson, J. O. , & Hamel, K. S. (1983). Decomposition of *Cladophora*. I. Field studies in the Peel‐Harvey estuarine system, Western Australia. Botanica Marina, 26(4), 165–171. 10.1515/botm.1983.26.4.165

[jpy70031-bib-0025] Bligh, M. , Nguyen, N. , Buck‐Wiese, H. , Vidal‐Melgosa, S. , & Hehemann, J. H. (2022). Structures and functions of algal glycans shape their capacity to sequester carbon in the ocean. Current Opinion in Chemical Biology, 71, 102204. 10.1016/j.cbpa.2022.102204 36155346

[jpy70031-bib-0026] Boldreel, E. , Attard, K. , Hancke, K. , & Glud, R. (2023). Microbial degradation dynamics of farmed kelp deposits from *Saccharina latissima* and *Alaria esculenta* . Marine Ecology Progress Series, 709, 1–15. 10.3354/meps14285

[jpy70031-bib-0027] Bourguès, S. , Auby, I. , De Wit, R. , & Labourg, P. (1996). Differential anaerobic decomposition of seagrass (*Zostera noltii*) and macroalgal (*Monostroma obscurum*) biomass from Arcachon Bay (France). Hydrobiologia, 329, 121–131. 10.1007/BF00034552

[jpy70031-bib-0028] Braeckman, U. , Pasotti, F. , Vázquez, S. , Zacher, K. , Hoffmann, R. , Elvert, M. , Marchant, H. , Buckner, C. , Quartino, M. L. , Mác Cormack, W. , Soetaert, K. , Wenzhöfer, F. , & Vanreusel, A. (2019). Degradation of macroalgal detritus in shallow coastal Antarctic sediments. Limnology and Oceanography, 64(4), 1423–1441. 10.1002/lno.11125 31598006 PMC6774326

[jpy70031-bib-0029] Brooks, M. E. , Kristensen, K. , van Benthem, K. J. , Magnusson, A. , Berg, C. W. , Nielsen, A. , Skaug, H. J. , Maechler, M. , & Bolker, B. M. (2017). glmmTMB balances speed and flexibility among packages for zero‐inflated generalized linear mixed modeling. The R Journal, 9(2), 378–400. 10.32614/RJ-2017-066

[jpy70031-bib-0030] Brouwer, P. E. M. (1996). Decomposition in situ of the sublittoral Antarctic macroalga *Desmarestia anceps* Montagne. Polar Biology, 16(2), 129–137. 10.1007/BF02390433

[jpy70031-bib-0031] Brunet, M. , de Bettignies, F. , Le Duff, N. , Tanguy, G. , Davoult, D. , Leblanc, C. , Gobet, A. , & Thomas, F. (2021). Accumulation of detached kelp biomass in a subtidal temperate coastal ecosystem induces succession of epiphytic and sediment bacterial communities. Environmental Microbiology, 23(3), 1638–1655. 10.1111/1462-2920.15389 33400326 PMC8248336

[jpy70031-bib-0032] Buchsbaum, R. , Valiela, I. , Swain, T. , Dzierzeski, M. , & Allen, S. (1991). Available and refractory nitrogen in detritus of coastal vascular plants and macroalgae. Marine Ecology Progress Series, 72, 131–143. 10.3354/meps072131

[jpy70031-bib-0033] Buck‐Wiese, H. , Andskog, M. A. , Nguyen, N. P. , Bligh, M. , Asmala, E. , Vidal‐Melgosa, S. , Liebeke, M. , Gustafsson, C. , & Hehemann, J. H. (2023). Fucoid brown algae inject fucoidan carbon into the ocean. Proceedings of the National Academy of Sciences of the United States of America, 120(1), e2210561119. 10.1073/pnas.2210561119 36584294 PMC9910443

[jpy70031-bib-0034] Burke, I. C. , Kaye, J. P. , Bird, S. P. , Hall, S. A. , McCulley, R. L. , & Sommerville, G. L. (2004). Evaluating and testing models of terrestrial biogeochemistry: The role of temperature in controlling decomposition. In C. D. Canham , J. J. Cole , & W. K. Lauenroth (Eds.), Models in ecosystem science (pp. 225–253). Princeton University Press. 10.1515/9780691228846-015

[jpy70031-bib-0035] Cai, J. , Lovatelli, A. , Aguilar‐Manjarrez, J. , Cornish, L. , Dabbadie, L. , Desrochers, A. , Diffey, S. , Garrido Gamarro, E. , Geehan, J. , Hurtado, A. , Lucente, D. , Mair, G. , Miao, W. , Potin, P. , Przybyla, C. , Reantaso, M. , Roubach, R. , Tauati, M. , Yuan, X. , … Yuan, X. (2021). Seaweeds and microalgae: An overview for unlocking their potential in global aquaculture development. *FAO Fisheries and Aquaculture Circular*, *1229*. 10.4060/cb5670en

[jpy70031-bib-0036] Cai, R. , & Jiao, N. (2023). Recalcitrant dissolved organic matter and its major production and removal processes in the ocean. Deep Sea Research Part I: Oceanographic Research Papers, 191, 103922. 10.1016/j.dsr.2022.103922

[jpy70031-bib-0037] Cartraud, A. E. , Lavery, P. S. , Rae, C. M. , & Hyndes, G. A. (2021). Pathways to spatial subsidies by kelp in seagrass meadows. Estuaries and Coasts, 44(2), 468–480. 10.1007/s12237-020-00860-8

[jpy70031-bib-0038] Castaldelli, G. , Welsh, D. T. , Flachi, G. , Zucchini, G. , Colombo, G. , Rossi, R. , & Fano, E. A. (2003). Decomposition dynamics of the bloom forming macroalga *Ulva rigida* C. Agardh determined using a 14C‐carbon radio‐tracer technique. Aquatic Botany, 75(2), 111–122. 10.1016/S0304-3770(02)00167-5

[jpy70031-bib-0039] Catenazzi, A. , & Donnelly, M. A. (2007). Role of supratidal invertebrates in the decomposition of beach‐cast green algae *Ulva* sp. Marine Ecology Progress Series, 349, 33–42. 10.3354/meps07106

[jpy70031-bib-0040] Chabbi, A. , Kögel‐Knabner, I. , & Rumpel, C. (2009). Stabilised carbon in subsoil horizons is located in spatially distinct parts of the soil profile. Soil Biology and Biochemistry, 41(2), 256–261. 10.1016/j.soilbio.2008.10.033

[jpy70031-bib-0041] Chang, Y.‐E. , Liao, C.‐H. , Hsieh, H.‐H. , Gradngay Abedneko, V. , Hung, C.‐C. , & Lee, T.‐M. (2024). Lateral carbon export of macroalgae and seagrass from diverse habitats contributes particulate organic carbon in the deep sea of the northern South China Sea. Marine Pollution Bulletin, 206, 116672. 10.1016/j.marpolbul.2024.116672 39047601

[jpy70031-bib-0042] Chassain, J. , Vieublé Gonod, L. , Chenu, C. , & Joimel, S. (2021). Role of different size classes of organisms in cropped soils: What do litterbag experiments tell us? A meta‐analysis. Soil Biology and Biochemistry, 162, 108394. 10.1016/j.soilbio.2021.108394

[jpy70031-bib-0043] Chen, J. , Li, H. , Zhang, Z. , He, C. , Shi, Q. , Jiao, N. , & Zhang, Y. (2020). DOC dynamics and bacterial community succession during long‐term degradation of *Ulva prolifera* and their implications for the legacy effect of green tides on refractory DOC pool in seawater. Water Research, 185, 116268. 10.1016/j.watres.2020.116268 32784034

[jpy70031-bib-0044] Chown, S. L. (1996). Kelp degradation by *Paractora trichosterna* (Thomson) (Diptera: Helcomyzidae) at sub‐Antarctic South Georgia. Polar Biology, 16(3), 171–178. 10.1007/s003000050042

[jpy70031-bib-0045] Ciancia, M. , Fernández, P. V. , & Leliaert, F. (2020). Diversity of sulfated polysaccharides from cell walls of coenocytic green algae and their structural relationships in view of green algal evolution. Frontiers in Plant Science, 11, 554585. 10.3389/fpls.2020.554585 33133113 PMC7550628

[jpy70031-bib-0046] Colombini, I. , Aloia, A. , Fallaci, M. , Pezzoli, G. , & Chelazzi, L. (2000). Temporal and spatial use of stranded wrack by the macrofauna of a tropical sandy beach. Marine Biology, 136(3), 531–541. 10.1007/s002270050713

[jpy70031-bib-0047] Conant, R. T. , Ryan, M. G. , Ågren, G. I. , Birge, H. E. , Davidson, E. A. , Eliasson, P. E. , Evans, S. E. , Frey, S. D. , Giardina, C. P. , Hopkins, F. M. , Hyvönen, R. , Kirschbaum, M. U. F. , Lavallee, J. M. , Leifeld, J. , Parton, W. J. , Megan Steinweg, J. , Wallenstein, M. D. , Martin Wetterstedt, J. Å. , & Bradford, M. A. (2011). Temperature and soil organic matter decomposition rates—Synthesis of current knowledge and a way forward. Global Change Biology, 17(11), 3392–3404. 10.1111/j.1365-2486.2011.02496.x

[jpy70031-bib-0048] Connell, S. , Foster, M. , & Airoldi, L. (2014). What are algal turfs? Towards a better description of turfs. Marine Ecology Progress Series, 495, 299–307. 10.3354/meps10513

[jpy70031-bib-0049] Conover, J. , Green, L. A. , & Thornber, C. S. (2016). Biomass decay rates and tissue nutrient loss in bloom and non‐bloom‐forming macroalgal species. Estuarine, Coastal and Shelf Science, 178, 58–64. 10.1016/j.ecss.2016.05.018

[jpy70031-bib-0050] Cornwell, W. K. , Cornelissen, J. H. C. , Amatangelo, K. , Dorrepaal, E. , Eviner, V. T. , Godoy, O. , Hobbie, S. E. , Hoorens, B. , Kurokawa, H. , Pérez‐Harguindeguy, N. , Quested, H. M. , Santiago, L. S. , Wardle, D. A. , Wright, I. J. , Aerts, R. , Allison, S. D. , Van Bodegom, P. , Brovkin, V. , Chatain, A. , … Westoby, M. (2008). Plant species traits are the predominant control on litter decomposition rates within biomes worldwide. Ecology Letters, 11(10), 1065–1071. 10.1111/j.1461-0248.2008.01219.x 18627410

[jpy70031-bib-0051] de Bettignies, F. , Dauby, P. , Thomas, F. , Gobet, A. , Delage, L. , Bohner, O. , Loisel, S. , & Davoult, D. (2020). Degradation dynamics and processes associated with the accumulation of *Laminaria hyperborea* (Phaeophyceae) kelp fragments: An in situ experimental approach. Journal of Phycology, 56(6), 1481–1492. 10.1111/jpy.13041 32557584

[jpy70031-bib-0052] de Bettignies, T. , Wernberg, T. , Lavery, P. S. , Vanderklift, M. A. , & Mohring, M. B. (2013). Contrasting mechanisms of dislodgement and erosion contribute to production of kelp detritus. Limnology and Oceanography, 58(5), 1680–1688. 10.4319/lo.2013.58.5.1680

[jpy70031-bib-0053] Deniaud‐Bouët, E. , Hardouin, K. , Potin, P. , Kloareg, B. , & Hervé, C. (2017). A review about brown algal cell walls and fucose‐containing sulfated polysaccharides: Cell wall context, biomedical properties and key research challenges. Carbohydrate Polymers, 175, 395–408. 10.1016/j.carbpol.2017.07.082 28917882

[jpy70031-bib-0054] Deniaud‐Bouët, E. , Kervarec, N. , Michel, G. , Tonon, T. , Kloareg, B. , & Hervé, C. (2014). Chemical and enzymatic fractionation of cell walls from Fucales: Insights into the structure of the extracellular matrix of brown algae. Annals of Botany, 114(6), 1203–1216. 24875633 10.1093/aob/mcu096 PMC4195554

[jpy70031-bib-0055] Dittmar, T. , Lennartz, S. T. , Buck‐Wiese, H. , Hansell, D. A. , Santinelli, C. , Vanni, C. , Blasius, B. , & Hehemann, J.‐H. (2021). Enigmatic persistence of dissolved organic matter in the ocean. Nature Reviews Earth and Environment, 2(8), 570–583. 10.1038/s43017-021-00183-7

[jpy70031-bib-0056] Dolliver, J. , & O'Connor, N. (2022a). Whole system analysis is required to determine the fate of macroalgal carbon: A systematic review. Journal of Phycology, 58(3), 364–376. 10.1111/jpy.13251 35397178 PMC9325415

[jpy70031-bib-0057] Dolliver, J. , & O'Connor, N. E. (2022). Estimating growth, loss and potential carbon sequestration of farmed kelp: A case study of *Saccharina latissima* at Strangford lough, Northern Ireland. Applied Phycology, 3(1), 324–339. 10.1080/26388081.2022.2081934

[jpy70031-bib-0058] Domozych, D. (2019). Algal cell walls. In Encyclopedia of life sciences. John Wiley & Sons, Ltd. 10.1002/9780470015902.a0000315.pub4

[jpy70031-bib-0059] Domozych, D. , Ciancia, M. , Fangel, J. U. , Mikkelsen, M. D. , Ulvskov, P. , & Willats, W. G. (2012). The cell walls of green algae: A journey through evolution and diversity. Frontiers in Plant Science, 3, 82. 10.3389/fpls.2012.00082 22639667 PMC3355577

[jpy70031-bib-0060] Duarte, C. M. , Gattuso, J. P. , Hancke, K. , Gundersen, H. , Filbee‐Dexter, K. , Pedersen, M. F. , Middelburg, J. J. , Burrows, M. T. , Krumhansl, K. A. , Wernberg, T. , Moore, P. , Pessarrodona, A. , Ørberg, S. B. , Pinto, I. S. , Assis, J. , Queirós, A. M. , Smale, D. A. , Bekkby, T. , Serrão, E. A. , & Krause‐Jensen, D. (2022). Global estimates of the extent and production of macroalgal forests. Global Ecology and Biogeography, 31(7), 1422–1439. 10.1111/geb.13515

[jpy70031-bib-0061] Dufour, C. , Probert, P. , & Savage, C. (2012). Macrofaunal colonisation of stranded *Durvillaea* *a* *ntarctica* on a southern New Zealand exposed sandy beach. New Zealand Journal of Marine and Freshwater Research, 46(3), 369–383. 10.1080/00288330.2012.676557

[jpy70031-bib-0062] Duggins, D. O. , & Eckman, J. E. (1997). Is kelp detritus a good food for suspension feeders? Effects of kelp species, age and secondary metabolites. Marine Biology, 128(3), 489–495. 10.1007/s002270050115

[jpy70031-bib-0063] Duggins, D. O. , Gómez‐Buckley, M. C. , Buckley, R. M. , Lowe, A. T. , Galloway, A. W. E. , & Dethier, M. N. (2016). Islands in the stream: Kelp detritus as faunal magnets. Marine Biology, 163(1), 17. 10.1007/s00227-015-2781-y

[jpy70031-bib-0064] Dungait, J. A. J. , Hopkins, D. W. , Gregory, A. S. , & Whitmore, A. P. (2012). Soil organic matter turnover is governed by accessibility not recalcitrance. Global Change Biology, 18(6), 1781–1796. 10.1111/j.1365-2486.2012.02665.x

[jpy70031-bib-0065] Endo, H. , Inomata, E. , Gao, X. , Kinoshita, J. , Sato, Y. , & Agatsuma, Y. (2020). Heat stress promotes nitrogen accumulation in meristems via apical blade erosion in a brown macroalga with intercalary growth. Frontiers in Marine Science, 7, 1–10. 10.3389/fmars.2020.575721 32802822

[jpy70031-bib-0066] Enríquez, S. , Duarte, C. M. , & Sand‐Jensen, K. (1993). Patterns in decomposition rates among photosynthetic organisms: The importance of detritus C:N:P content. Oecologia, 94(4), 457–471. 10.1007/BF00566960 28313985

[jpy70031-bib-0067] Erlania , Bellgrove, A. , Macreadie, P. I. , Young, M. A. , Holland, O. J. , Clark, Z. , Ierodiaconou, D. , Carvalho, R. C. , Kennedy, D. , & Miller, A. D. (2023). Patterns and drivers of macroalgal ‘blue carbon’ transport and deposition in near‐shore coastal environments. Science of the Total Environment, 890, 164430. 10.1016/j.scitotenv.2023.164430 37247743

[jpy70031-bib-0068] Fenchel, T. , & Finlay, B. (2008). Oxygen and the spatial structure of microbial communities. Biological Reviews, 83(4), 553–569. 10.1111/j.1469-185X.2008.00054.x 18823390

[jpy70031-bib-0069] Feng, X. , Li, H. , Zhang, Z. , Xiong, T. , Shi, X. , He, C. , Shi, Q. , Jiao, N. , & Zhang, Y. (2022). Microbial‐mediated contribution of kelp detritus to different forms of oceanic carbon sequestration. Ecological Indicators, 142(July), 109186. 10.1016/j.ecolind.2022.109186

[jpy70031-bib-0070] Filbee‐Dexter, K. , Feehan, C. J. , Smale, D. A. , Krumhansl, K. A. , Augustine, S. , de Bettignies, F. , Burrows, M. T. , Byrnes, J. E. K. , Campbell, J. , Davoult, D. , Dunton, K. H. , Franco, J. N. , Garrido, I. , Grace, S. P. , Hancke, K. , Johnson, L. E. , Konar, B. , Moore, P. J. , Norderhaug, K. M. , … Wernberg, T. (2022). Kelp carbon sink potential decreases with warming due to accelerating decomposition. PLoS Biology, 20(8), 1–22. 10.1371/journal.pbio.3001702 PMC9352061 35925899

[jpy70031-bib-0071] Filbee‐Dexter, K. , Pessarrodona, A. , Pedersen, M. F. , Wernberg, T. , Duarte, C. M. , Assis, J. , Bekkby, T. , Burrows, M. T. , Carlson, D. F. , Gattuso, J.‐P. , Gundersen, H. , Hancke, K. , Krumhansl, K. A. , Kuwae, T. , Middelburg, J. J. , Moore, P. J. , Queirós, A. M. , Smale, D. A. , Sousa‐Pinto, I. , … Krause‐Jensen, D. (2024). Carbon export from seaweed forests to deep ocean sinks. Nature Geoscience, 17(6), 552–559. 10.1038/s41561-024-01449-7

[jpy70031-bib-0072] Filbee‐Dexter, K. , & Wernberg, T. (2018). Rise of turfs: A new battlefront for globally declining kelp forests. Bioscience, 68(2), 64–76. 10.1093/biosci/bix147

[jpy70031-bib-0073] Filbee‐Dexter, K. , Wernberg, T. , Norderhaug, K. M. , Ramirez‐Llodra, E. , & Pedersen, M. F. (2018). Movement of pulsed resource subsidies from kelp forests to deep fjords. Oecologia, 187(1), 291–304. 10.1007/s00442-018-4121-7 29605871

[jpy70031-bib-0074] Fischer, G. , & Wiencke, C. (1992). Stable carbon isotope composition, depth distribution and fate of macroalgae from the Antarctic peninsula region. Polar Biology, 12(3–4), 341–348. 10.1007/BF00243105

[jpy70031-bib-0075] Fonseca, V. G. (2018). Pitfalls in relative abundance estimation using eDNA metabarcoding. Molecular Ecology Resources, 18(5), 923–926. 10.1111/1755-0998.12902

[jpy70031-bib-0076] Franzitta, G. , Hanley, M. E. , Airoldi, L. , Baggini, C. , Bilton, D. T. , Rundle, S. D. , & Thompson, R. C. (2015). Home advantage? Decomposition across the freshwater‐estuarine transition zone varies with litter origin and local salinity. Marine Environmental Research, 110, 1–7. 10.1016/j.marenvres.2015.07.012 26247807

[jpy70031-bib-0077] Frontier, N. , De Bettignies, F. , Foggo, A. , & Davoult, D. (2021). Sustained productivity and respiration of degrading kelp detritus in the shallow benthos: Detached or broken, but not dead. Marine Environmental Research, 166, 105277. 10.1016/j.marenvres.2021.105277 33592375

[jpy70031-bib-0078] Frontier, N. , Mulas, M. , Foggo, A. , & Smale, D. A. (2022). The influence of light and temperature on detritus degradation rates for kelp species with contrasting thermal affinities. Marine Environmental Research, 173(November), 105529. 10.1016/j.marenvres.2021.105529 34800869

[jpy70031-bib-0079] Fujita, R. , Augyte, S. , Bender, J. , Brittingham, P. , Buschmann, A. H. , Chalfin, M. , Collins, J. , Davis, K. A. , Gallagher, J. B. , Gentry, R. , Gruby, R. L. , Kleisner, K. , Moritsch, M. , Price, N. , Roberson, L. , Taylor, J. , & Yarish, C. (2023). Seaweed blue carbon: Ready? Or not? Marine Policy, 155, 105747. 10.1016/j.marpol.2023.105747

[jpy70031-bib-0080] Gallagher, J. B. , Shelamoff, V. , & Layton, C. (2022). Seaweed ecosystems may not mitigate CO_2_ emissions. ICES Journal of Marine Science, 79(3), 585–592. 10.1093/icesjms/fsac011

[jpy70031-bib-0081] Gao, Y. , Zhang, Y. , Du, M. , Lin, F. , Jiang, W. , Li, W. , Li, F. , Lv, X. , Fang, J. , & Jiang, Z. (2021). Dissolved organic carbon from cultured kelp *Saccharina japonica*: Production, bioavailability, and bacterial degradation rates. Aquaculture Environment Interactions, 13, 101–110. 10.3354/AEI00393

[jpy70031-bib-0082] Geiselman, J. A. , & McConnell, O. J. (1981). Polyphenols in brown algae *Fucus vesiculosus* and *Ascophyllum nodosum*: Chemical defences against the marine herbivorous snail, *Littorina littorea* . Journal of Chemical Ecology, 7(6), 1115–1133. 10.1007/BF00987632 24420835

[jpy70031-bib-0083] Gilson, A. R. , Smale, D. A. , Burrows, M. T. , & O'Connor, N. E. (2021). Spatio‐temporal variability in the deposition of beach‐cast kelp (wrack) and inter‐specific differences in degradation rates. Marine Ecology Progress Series, 674, 89–102. 10.3354/meps13825

[jpy70031-bib-0084] Gladstone‐Gallagher, R. V. , Lohrer, A. M. , Lundquist, C. J. , & Pilditch, C. A. (2016). Effects of detrital subsidies on soft‐sediment ecosystem function are transient and source‐dependent. PLoS ONE, 11(5), e0154790. 10.1371/journal.pone.0154790 27138563 PMC4854381

[jpy70031-bib-0085] Glud, R. N. (2008). Oxygen dynamics of marine sediments. Marine Biology Research, 4(4), 243–289. 10.1080/17451000801888726

[jpy70031-bib-0086] Gómez, M. , Barreiro, F. , López, J. , & Lastra, M. (2018). Effect of upper beach macrofauna on nutrient cycling of sandy beaches: Metabolic rates during wrack decay. Marine Biology, 165(8), 133. 10.1007/s00227-018-3392-1

[jpy70031-bib-0087] Griffiths, C. L. , Stenton‐Dozey, J. M. E. , & Koop, K. (1983). Kelp wrack and the flow of energy through a sandy beach ecosystem. In A. McLachlan , & T. Erasmus (Eds.), Sandy beaches as ecosystems (pp. 547–556). Springer. 10.1007/978-94-017-2938-3_42

[jpy70031-bib-0088] Gruber, N. , Clement, D. , Carter, B. R. , Feely, R. A. , van Heuven, S. , Hoppema, M. , Ishii, M. , Key, R. M. , Kozyr, A. , Lauvset, S. K. , Lo Monaco, C. , Mathis, J. T. , Murata, A. , Olsen, A. , Perez, F. F. , Sabine, C. L. , Tanhua, T. , & Wanninkhof, R. (2019). The oceanic sink for anthropogenic CO_2_ from 1994 to 2007. Science, 363(6432), 1193–1199. 10.1126/science.aau5153 30872519

[jpy70031-bib-0089] Guiry, M. D. (2024). How many species of algae are there? A reprise. Four kingdoms, 14 phyla, 63 classes and still growing. Journal of Phycology, 60(2), 214–228. 10.1111/jpy.13431 38245909

[jpy70031-bib-0090] Handa, I. T. , Aerts, R. , Berendse, F. , Berg, M. P. , Bruder, A. , Butenschoen, O. , Chauvet, E. , Gessner, M. O. , Jabiol, J. , Makkonen, M. , McKie, B. G. , Malmqvist, B. , Peeters, E. T. H. M. , Scheu, S. , Schmid, B. , van Ruijven, J. , Vos, V. C. A. , & Hättenschwiler, S. (2014). Consequences of biodiversity loss for litter decomposition across biomes. Nature, 509(7499), 218–221. 10.1038/nature13247 24805346

[jpy70031-bib-0091] Hanisak, M. D. (1993). Nitrogen release from decomposing seaweeds: Species and temperature effects. Journal of Applied Phycology, 5(2), 175–181. 10.1007/BF00004014

[jpy70031-bib-0092] Hansell, D. A. (2001). Marine dissolved organic matter and the carbon cycle. Oceanography, 14(4), 41–49. 10.5670/oceanog.2001.05

[jpy70031-bib-0093] Hansell, D. A. (2013). Recalcitrant dissolved organic carbon fractions. Annual Review of Marine Science, 5(1), 421–445. 10.1146/annurev-marine-120710-100757 22881353

[jpy70031-bib-0094] Hansell, D. A. , Carlson, C. A. , Repeta, D. J. , & Schlitzer, R. (2009). Dissolved organic matter in the ocean: A controversy stimulates new insights. Oceanography, 22(4), 202–211. 10.5670/oceanog.2009.109

[jpy70031-bib-0095] Hansen, K. E. , Giraudeau, J. , Limoges, A. , Massé, G. , Rudra, A. , Wacker, L. , Sanei, H. , Pearce, C. , & Seidenkrantz, M.‐S. (2022). Characterization of organic matter in marine sediments to estimate age offset of bulk radiocarbon dating. Quaternary Geochronology, 67, 101242. 10.1016/j.quageo.2021.101242

[jpy70031-bib-0096] Haram, L. , Sotka, E. , & Byers, J. (2020). Effects of novel, non‐native detritus on decomposition and invertebrate community assemblage. Marine Ecology Progress Series, 643, 49–61. 10.3354/meps13335

[jpy70031-bib-0097] Harrison, P. G. (1989). Detrital processing in seagrass systems: A review of factors affecting decay rates, remineralization and detritivory. Aquatic Botany, 35(3), 263–288. 10.1016/0304-3770(89)90002-8

[jpy70031-bib-0098] Harrold, C. , & Reed, D. C. (1985). Food availability, sea urchin grazing, and kelp forest Community structure. Ecology, 66(4), 1160–1169. 10.2307/1939168

[jpy70031-bib-0099] Hartig, F. (2022). *DHARMa: Residual Diagnostics for Hierarchical (Multi‐Level/Mixed) Regression Models* [Computer software]. https://CRAN.R‐project.org/package=DHARMa

[jpy70031-bib-0100] Hebbali, A. (2024). *olsrr: Tools for Building OLS Regression Models*. (Version 0.6.1) [Computer software]. https://CRAN.R‐project.org/package=olsrr

[jpy70031-bib-0101] Hill, J. M. , & McQuaid, C. D. (2009). Variability in the fractionation of stable isotopes during degradation of two intertidal red algae. Estuarine, Coastal and Shelf Science, 82(3), 397–405. 10.1016/j.ecss.2009.02.001

[jpy70031-bib-0102] Hill, R. , Bellgrove, A. , Macreadie, P. I. , Petrou, K. , Beardall, J. , Steven, A. , & Ralph, P. J. (2015). Can macroalgae contribute to blue carbon? An Australian perspective. Limnology and Oceanography, 60(5), 1689–1706. 10.1002/lno.10128

[jpy70031-bib-0103] Holzinger, A. , Herburger, K. , Kaplan, F. , & Lewis, L. A. (2015). Desiccation tolerance in the chlorophyte green alga *Ulva compressa*: Does cell wall architecture contribute to ecological success? Planta, 242(2), 477–492. 10.1007/s00425-015-2292-6 25896374 PMC4498240

[jpy70031-bib-0104] Hosen, M. J. , Hossain, M. , Martinez, J. G. , Sumaya, N. H. , Mei, Y. , & Manandhar, S. (2011). Effect of bacterivorous and predatory nematodes on macroalgal detritus decomposition. Proceedings of the Pakistan Academy of Sciences, 48(3), 137–142.

[jpy70031-bib-0105] Hu, X. , Cao, Y. , Zhao, X. , Su, H. , Wen, G. , & Yang, Y. (2023). Effect of bacterial community succession on environmental factors during litter decomposition of the seaweed *Gracilaria lemaneiformis* . Marine Pollution Bulletin, 197, 115797. 10.1016/j.marpolbul.2023.115797 37984092

[jpy70031-bib-0106] Huang, X. , An, S. , Chen, S. , Dai, J. , Liu, J. , Wen, S. , Li, T. , Xing, P. , & Du, Y. (2023). Transformation of algal‐dissolved organic matter via sunlight‐induced photochemical and microbial processes: Interactions between two processes. Environmental Science and Pollution Research International, 30(18), 52969–52981. 10.1007/s11356-023-26024-2 36843169

[jpy70031-bib-0107] Hunter, R. D. (1976). Changes in carbon and nitrogen content during decomposition of three macrophytes in freshwater and marine environments. Hydrobiologia, 51, 9–128.

[jpy70031-bib-0108] Hurd, C. L. , Law, C. S. , Bach, L. T. , Britton, D. , Hovenden, M. , Paine, E. R. , Raven, J. A. , Tamsitt, V. , & Boyd, P. W. (2022). Forensic carbon accounting: Assessing the role of seaweeds for carbon sequestration. Journal of Phycology, 58(3), 347–363. 10.1111/jpy.13249 35286717

[jpy70031-bib-0109] Hwang, J.‐M. , Kim, H.‐G. , Kim, H. , Hwang, C.‐H. , & Oh, C.‐W. (2023). Meiofaunal assemblages associated with macroalgal detritus decomposition. Regional Studies in Marine Science, 68, 103285. 10.1016/j.rsma.2023.103285

[jpy70031-bib-0110] Hyndes, G. A. , Berdan, E. L. , Duarte, C. , Dugan, J. E. , Emery, K. A. , Hambäck, P. A. , Henderson, C. J. , Hubbard, D. M. , Lastra, M. , Mateo, M. A. , Olds, A. , & Schlacher, T. A. (2022). The role of inputs of marine wrack and carrion in sandy‐beach ecosystems: A global review. Biological Reviews, 97(6), 2127–2161. 10.1111/brv.12886 35950352 PMC9804821

[jpy70031-bib-0111] Ihua, M. W. , Guihéneuf, F. , Mohammed, H. , Margassery, L. M. , Jackson, S. A. , Stengel, D. B. , Clarke, D. J. , & Dobson, A. D. W. (2019). Microbial population changes in decaying *Ascophyllum nodosum* result in macroalgal‐polysaccharide‐degrading bacteria with potential applicability in enzyme‐assisted extraction technologies. Marine Drugs, 17(4), 200. 10.3390/md17040200 30934874 PMC6520818

[jpy70031-bib-0112] Imran, M. , Poduval, P. B. , & Ghadi, S. C. (2017). Bacterial degradation of algal polysaccharides in marine ecosystem. In M. Naik , & S. Dubey (Eds.), Marine pollution and microbial remediation (pp. 189–203). Springer. 10.1007/978-981-10-1044-6_12

[jpy70031-bib-0113] Ince, R. , Hyndes, G. , Lavery, P. , & Vanderklift, M. (2007). Marine macrophytes directly enhance abundances of sandy beach fauna through provision of food and habitat. ECU Publications, 74(1–2), 77–86. 10.1016/j.ecss.2007.03.029

[jpy70031-bib-0114] Intergovernmental Panel on Climate Change (IPCC) . (2013). Climate change 2013: The physical science basis. Contribution of working group I (WGI) to the fifth assessment report (AR5) of the intergovernmental panel on climate change (IPCC). Cambridge University Press.

[jpy70031-bib-0115] Jayathilake, D. R. M. , & Costello, M. J. (2020). A modelled global distribution of the kelp biome. Biological Conservation, 252, 108815. 10.1016/j.biocon.2020.108815

[jpy70031-bib-0116] Jenny, H. , Gessel, S. P. , & Bingham, F. T. (1949). Comparative study of decomposition rates of organic matter in temperate and tropical regions. Soil Science, 68(6), 419–432.

[jpy70031-bib-0117] Jiao, N. , Herndl, G. J. , Hansell, D. A. , Benner, R. , Kattner, G. , Wilhelm, S. W. , Kirchman, D. L. , Weinbauer, M. G. , Luo, T. , Chen, F. , & Azam, F. (2010). Microbial production of recalcitrant dissolved organic matter: Long‐term carbon storage in the global ocean. Nature Reviews Microbiology, 8(8), 593–599. 10.1038/nrmicro2386 20601964

[jpy70031-bib-0118] Jiao, N. , Robinson, C. , Azam, F. , Thomas, H. , Baltar, F. , Dang, H. , Hardman‐Mountford, N. J. , Johnson, M. , Kirchman, D. L. , Koch, B. P. , Legendre, L. , Li, C. , Liu, J. , Luo, T. , Luo, Y. W. , Mitra, A. , Romanou, A. , Tang, K. , Wang, X. , … Zhang, R. (2014). Mechanisms of microbial carbon sequestration in the ocean – future research directions. Biogeosciences, 11(19), 5285–5306. 10.5194/bg-11-5285-2014

[jpy70031-bib-0119] Johnson, D. L. , & Richardson, P. L. (1977). On the wind‐induced sinking of *Sargassum* . Journal of Experimental Marine Biology and Ecology, 28(3), 255–267. 10.1016/0022-0981(77)90095-8

[jpy70031-bib-0120] Josselyn, M. N. , & Mathieson, A. C. (1980). Seasonal influx and decomposition of autochthonous macrophyte litter in a north temperate estuary. Hydrobiologia, 71(3), 197–208. 10.1007/BF03216236

[jpy70031-bib-0121] Kennedy, H. , Beggins, J. , Duarte, C. M. , Fourqurean, J. W. , Holmer, M. , Marbà, N. , & Middelburg, J. J. (2010). Seagrass sediments as a global carbon sink: Isotopic constraints. Global Biogeochemical Cycles, 24(4), GB4026. 10.1029/2010GB003848

[jpy70031-bib-0122] Kharbush, J. J. , Close, H. G. , Van Mooy, B. A. S. , Arnosti, C. , Smittenberg, R. H. , Le Moigne, F. A. C. , Mollenhauer, G. , Scholz‐Böttcher, B. , Obreht, I. , Koch, B. P. , Becker, K. W. , Iversen, M. H. , & Mohr, W. (2020). Particulate organic carbon deconstructed: Molecular and chemical composition of particulate organic carbon in the ocean. Frontiers in Marine Science, 7, 518. 10.3389/fmars.2020.00518

[jpy70031-bib-0123] Kim, H.‐G. , Hawkins, L. E. , Godbold, J. A. , Bohn, K. , Khim, J. S. , & Hawkins, S. J. (2021). The influence of the composition of algal detritus on nematode assemblages. Regional Studies in Marine Science, 48, 102004. 10.1016/j.rsma.2021.102004

[jpy70031-bib-0124] Klap, V. , Hemminga, M. , & Boon, J. (2000). Retention of lignin in seagrasses: Angiosperms that returned to the sea. Marine Ecology Progress Series, 194, 1–11. 10.3354/meps194001

[jpy70031-bib-0125] Kleber, M. (2010). What is recalcitrant soil organic matter? Environmental Chemistry, 7(4), 320. 10.1071/EN10006

[jpy70031-bib-0126] Kleber, M. , Nico, P. S. , Plante, A. , Filley, T. , Kramer, M. , Swanston, C. , & Sollins, P. (2011). Old and stable soil organic matter is not necessarily chemically recalcitrant: Implications for modeling concepts and temperature sensitivity. Global Change Biology, 17(2), 1097–1107. 10.1111/j.1365-2486.2010.02278.x

[jpy70031-bib-0127] Kloareg, B. , & Quatrano, R. S. (1988). Structure of the cell walls of marine algae and ecophysiological functions of the matrix polysaccharides. In M. Barnes (Ed.), Oceanography and marine biology, an annual review (Vol. 26, pp. 221–272). Aberdeen University Press.

[jpy70031-bib-0128] Kokubu, Y. , Komatsu, T. , Ito, M. , Hattori, T. , & Narimatsu, Y. (2012). Biomass of marine macrophyte debris on the ocean floor southeast of Hokkaido Island adjusted by experimental catch efficiency estimates. La Mer, 50(1–2), 11–22.

[jpy70031-bib-0129] Kokubu, Y. , Rothäusler, E. , Filippi, J. B. , Durieux, E. D. H. , & Komatsu, T. (2019). Revealing the deposition of macrophytes transported offshore: Evidence of their long‐distance dispersal and seasonal aggregation to the deep sea. Scientific Reports, 9(1), 4331. 10.1038/s41598-019-39982-w 30858431 PMC6411727

[jpy70031-bib-0130] Kotta, J. , Orav‐Kotta, H. , & Paalme, T. (2010). In situ evidence on the role of benthic invertebrates on the decomposition of drifting algal mats in a brackish water ecosystem. *2010 International Conference on Biosciences*, 139–144. 10.1109/BioSciencesWorld.2010.25.

[jpy70031-bib-0131] Krause‐Jensen, D. , & Duarte, C. M. (2016). Substantial role of macroalgae in marine carbon sequestration. Nature Geoscience, 9(10), 737–742. 10.1038/ngeo2790

[jpy70031-bib-0132] Krause‐Jensen, D. , Lavery, P. , Serrano, O. , Marba, N. , Masque, P. , & Duarte, C. M. (2018). Sequestration of macroalgal carbon: The elephant in the blue carbon room. Biology Letters, 14(6), 20180236. 10.1098/rsbl.2018.0236 29925564 PMC6030603

[jpy70031-bib-0133] Kristensen, E. (1994). Decomposition of macroalgae, vascular plants and sediment detritus in seawater: Use of stepwise thermogravimetry. Biogeochemistry, 26(1), 1–24. 10.1007/BF02180401

[jpy70031-bib-0134] Kristensen, E. , Andersen, F. Ø. , & Blackburn, T. H. (1992). Effects of benthic macrofauna and temperature on degradation of macroalgal detritus: The fate of organic carbon. Limnology and Oceanography, 37(7), 1404–1419. 10.4319/lo.1992.37.7.1404

[jpy70031-bib-0135] Kristensen, E. , & Mikkelsen, O. (2003). Impact of the burrow‐dwelling polychaete *Nereis diversicolor* on the degradation of fresh and aged macroalgal detritus in a coastal marine sediment. Marine Ecology Progress Series, 265, 141–153. 10.3354/meps265141

[jpy70031-bib-0136] Krumhansl, K. A. , Okamoto, D. K. , Rassweiler, A. , Novak, M. , Bolton, J. J. , Cavanaugh, K. C. , Connell, S. D. , Johnson, C. R. , Konar, B. , Ling, S. D. , Micheli, F. , Norderhaug, K. M. , Pérez‐Matus, A. , Sousa‐Pinto, I. , Reed, D. C. , Salomon, A. K. , Shears, N. T. , Wernberg, T. , Anderson, R. J. , … Byrnes, J. E. K. (2016). Global patterns of kelp forest change over the past half‐century. Proceedings of the National Academy of Sciences of the United States of America, 113(48), 13785–13790. 10.1073/pnas.1606102113 27849580 PMC5137772

[jpy70031-bib-0137] Krumhansl, K. A. , & Scheibling, R. (2012a). Detrital subsidy from subtidal kelp beds is altered by the invasive green alga *Codium fragile* ssp. *f* *ragile* . Marine Ecology Progress Series, 456, 73–85. 10.3354/meps09671

[jpy70031-bib-0138] Krumhansl, K. A. , & Scheibling, R. E. (2012b). Production and fate of kelp detritus. Marine Ecology Progress Series, 467, 281–302. 10.3354/meps09940

[jpy70031-bib-0139] Lacoursière‐Roussel, A. , Côté, G. , Leclerc, V. , & Bernatchez, L. (2016). Quantifying relative fish abundance with eDNA: A promising tool for fisheries management. Journal of Applied Ecology, 53(4), 1148–1157. 10.1111/1365-2664.12598

[jpy70031-bib-0140] Lage, O. M. , & Bondoso, J. (2014). Planctomycetes and macroalgae, a striking association. Frontiers in Microbiology, 5, 267. 10.3389/fmicb.2014.00267 24917860 PMC4042473

[jpy70031-bib-0141] Lal, R. (2007). Carbon sequestration. Philosophical Transactions of the Royal Society, B: Biological Sciences, 363(1492), 815–830. 10.1098/rstb.2007.2185 PMC2610111 17761468

[jpy70031-bib-0142] Lalli, C. , & Parsons, T. (1993). Biological oceanography: An introduction. Butterworth‐Heinemann.

[jpy70031-bib-0143] Lanari, M. , Claudino, M. C. , Garcia, A. M. , & Copertino, M. d. S. (2018). Changes in the elemental (C, N) and isotopic (δ13C, δ15N) composition of estuarine plants during diagenesis and implications for ecological studies. Journal of Experimental Marine Biology and Ecology, 500, 46–54. 10.1016/j.jembe.2017.12.013

[jpy70031-bib-0144] Lanari, M. , Copertino, M. S. , Colling, L. A. , & Bom, F. C. (2018). The impact of short‐term depositions of macroalgal blooms on widgeon‐grass meadows in a river‐dominated estuary. Harmful Algae, 78, 36–46. 10.1016/j.hal.2018.07.006 30196923

[jpy70031-bib-0145] LaRowe, D. E. , & Van Cappellen, P. (2011). Degradation of natural organic matter: A thermodynamic analysis. Geochimica et Cosmochimica Acta, 75(8), 2030–2042. 10.1016/j.gca.2011.01.020

[jpy70031-bib-0146] Lastra, M. , Page, H. M. , Dugan, J. E. , Hubbard, D. M. , & Rodil, I. F. (2008). Processing of allochthonous macrophyte subsidies by sandy beach consumers: Estimates of feeding rates and impacts on food resources. Marine Biology, 154(1), 163–174. 10.1007/s00227-008-0913-3

[jpy70031-bib-0147] Lecerf, A. (2017). Methods for estimating the effect of litterbag mesh size on decomposition. Ecological Modelling, 362, 65–68. 10.1016/j.ecolmodel.2017.08.011

[jpy70031-bib-0148] Lee, C. , Wakeham, S. , & Arnosti, C. (2004). Particulate organic matter in the sea: The composition conundrum. Ambio: A Journal of the Human Environment, 33(8), 565–575. 10.1579/0044-7447-33.8.565 15666690

[jpy70031-bib-0149] Lee, W.‐K. , & Ho, C.‐L. (2022). Ecological and evolutionary diversification of sulphated polysaccharides in diverse photosynthetic lineages: A review. Carbohydrate Polymers, 277, 118764. 10.1016/j.carbpol.2021.118764 34893214

[jpy70031-bib-0150] Legendre, L. , Rivkin, R. B. , Weinbauer, M. G. , Guidi, L. , & Uitz, J. (2015). The microbial carbon pump concept: Potential biogeochemical significance in the globally changing ocean. Progress in Oceanography, 134, 432–450. 10.1016/j.pocean.2015.01.008

[jpy70031-bib-0151] Lenth, R. V. (2024). *emmeans: Estimated Marginal Means, aka Least‐Squares Means* [Computer software]. https://CRAN.R‐project.org/package=emmeans

[jpy70031-bib-0152] Li, H. , Feng, X. , Xiong, T. , He, C. , Wu, W. , Shi, Q. , Jiao, N. , & Zhang, Y. (2023). Green tides significantly alter the molecular composition and properties of coastal DOC and perform dissolved carbon sequestration. Environmental Science & Technology, 57(1), 770–779. 10.1021/acs.est.2c05684 36511764

[jpy70031-bib-0153] Li, H. , Feng, X. , Xiong, T. , Shao, W. , Wu, W. , & Zhang, Y. (2023). Particulate organic carbon released during macroalgal growth has significant carbon sequestration potential in the ocean. Environmental Science & Technology, 57(48), 19723–19731. 10.1021/acs.est.3c04959 37963337

[jpy70031-bib-0154] Li, H. , Zhang, Z. , Xiong, T. , Tang, K. , He, C. , Shi, Q. , Jiao, N. , & Zhang, Y. (2022). Carbon sequestration in the form of recalcitrant dissolved organic carbon in a seaweed (kelp) farming environment. Environmental Science and Technology, 56(12), 9112–9122. 10.1021/acs.est.2c01535 35686906

[jpy70031-bib-0155] Lian, Y. , Wang, R. , Zheng, J. , Chen, W. , Chang, L. , Li, C. , & Yim, S. C. (2023). Carbon sequestration assessment and analysis in the whole life cycle of seaweed. Environmental Research Letters, 18(7), 074013. 10.1088/1748-9326/acdae9

[jpy70031-bib-0156] Liang, J. , Liu, J. , Zhan, Y. , Zhou, S. , Xue, C. X. , Sun, C. , Lin, Y. , Luo, C. , Wang, X. , & Zhang, X. H. (2021). Succession of marine bacteria in response to *Ulva prolifera*‐derived dissolved organic matter. Environment International, 155, 106687. 10.1016/j.envint.2021.106687 34144477

[jpy70031-bib-0157] Litchfield, S. G. , Schulz, K. G. , & Kelaher, B. P. (2020). The influence of plastic pollution and ocean change on detrital decomposition. Marine Pollution Bulletin, 158, 111354. 10.1016/j.marpolbul.2020.111354 32753168

[jpy70031-bib-0158] Littler, M. M. , Littler, D. S. , & Taylor, P. R. (1983). Evolutionary strategies in a tropical barrier reef system: Functional‐form groups of marine macroalgae. Journal of Phycology, 19(2), 229–237. 10.1111/j.0022-3646.1983.00229.x

[jpy70031-bib-0159] Liu, S. , Trevathan‐Tackett, S. M. , Ewers Lewis, C. J. , Huang, X. , & Macreadie, P. I. (2020). Macroalgal blooms trigger the breakdown of seagrass blue carbon. Environmental Science & Technology, 54(22), 14750–14760. 10.1021/acs.est.0c03720 33103882

[jpy70031-bib-0160] Liu, X. , Dunne, J. P. , Stock, C. A. , Harrison, M. J. , Adcroft, A. , & Resplandy, L. (2019). Simulating water residence time in the coastal ocean: A global perspective. Geophysical Research Letters, 46(23), 13910–13919. 10.1029/2019GL085097

[jpy70031-bib-0161] Lønborg, C. , Carreira, C. , Jickells, T. , & Álvarez‐Salgado, X. A. (2020). Impacts of global change on ocean dissolved organic carbon (DOC) cycling. Frontiers in Marine Science, 7, 466. 10.3389/fmars.2020.00466

[jpy70031-bib-0162] Lopes, M. L. , Martins, P. , Ricardo, F. , Rodrigues, A. M. , & Quintino, V. (2011). In situ experimental decomposition studies in estuaries: A comparison of *Phragmites australis* and *Fucus vesiculosus* . Estuarine, Coastal and Shelf Science, 92(4), 573–580. 10.1016/j.ecss.2011.02.014

[jpy70031-bib-0163] Lovelock, C. E. , Atwood, T. , Baldock, J. , Duarte, C. M. , Hickey, S. , Lavery, P. S. , Masque, P. , Macreadie, P. I. , Ricart, A. M. , Serrano, O. , & Steven, A. (2017). Assessing the risk of carbon dioxide emissions from blue carbon ecosystems. Frontiers in Ecology and the Environment, 15(5), 257–265. 10.1002/fee.1491

[jpy70031-bib-0164] Lovelock, C. E. , & Duarte, C. M. (2019). Dimensions of blue carbon and emerging perspectives. Biology Letters, 15(3), 20180781. 10.1098/rsbl.2018.0781 30836882 PMC6451379

[jpy70031-bib-0165] Lovelock, C. E. , Fourqurean, J. W. , & Morris, J. T. (2017). Modeled CO_2_ emissions from coastal wetland transitions to other land uses: Tidal marshes, mangrove forests, and seagrass beds. Frontiers in Marine Science, 4, 143. 10.3389/fmars.2017.00143

[jpy70031-bib-0166] Lozada, M. , Diéguez, M. C. , García, P. E. , & Dionisi, H. M. (2023). Microbial communities associated with kelp detritus in temperate and subantarctic intertidal sediments. Science of the Total Environment, 857(Pt 1), 159392. 10.1016/j.scitotenv.2022.159392 36240919

[jpy70031-bib-0167] Lozada, M. , Zabala, M. S. , García, P. E. , Diéguez, M. C. , Bigatti, G. , Fermani, P. , Unrein, F. , & Dionisi, H. M. (2022). Microbial assemblages associated with the invasive kelp *Undaria pinnatifida* in Patagonian coastal waters: Structure and alginolytic potential. Science of the Total Environment, 830, 154629. 10.1016/j.scitotenv.2022.154629 35337861

[jpy70031-bib-0168] Lucas, M. I. , Newell, R. C. , & Velimirov, B. (1981). Heterotrophic utilisation of mucilage released during fragmentation of kelp (*Ecklonia maxima* and *Laminaria pallida*). II. Differential utilisation of dissolved organic components from kelp mucilage. Marine Ecology Progress Series, 4, 43–55.

[jpy70031-bib-0169] Luo, H. , Dai, X. , Yang, Y. , & Xie, S. (2022). The evaluation of C, N, P release and contribution to the water environment during *Gracilaria* litters biomass decay. Estuarine, Coastal and Shelf Science, 276, 108052. 10.1016/j.ecss.2022.108052

[jpy70031-bib-0170] Luo, H. , Wang, Q. , Zhang, C. , Zhang, L. , & Yang, Y. (2021). Bioaccumulation and release of heavy metals during growth and decomposition of cultivated *Gracilaria lemaneiformis* . Marine Pollution Bulletin, 173, 113130. 10.1016/j.marpolbul.2021.113130 34814002

[jpy70031-bib-0171] Luo, H. , Xie, S. , Dai, X. , Wang, Q. , & Yang, Y. (2022). Biomass decomposition and heavy metal release from seaweed litter, *Gracilaria lemaneiformis*, and secondary pollution evaluation. Journal of Environmental Management, 310, 114729. 10.1016/j.jenvman.2022.114729 35192981

[jpy70031-bib-0172] Luo, J. , Wang, N. , Zhu, Y. , Wu, Z. , Ye, Z. , Christakos, G. , & Wu, J. (2024). Seasonal effects of fish, seaweed and abalone cultures on dissolved organic matter and carbon sequestration potential in Sansha Bay, China. Science of the Total Environment, 945, 174144. 10.1016/j.scitotenv.2024.174144 38901588

[jpy70031-bib-0173] Macreadie, P. I. , Anton, A. , Raven, J. A. , Beaumont, N. , Connolly, R. M. , Friess, D. A. , Kelleway, J. J. , Kennedy, H. , Kuwae, T. , Lavery, P. S. , Lovelock, C. E. , Smale, D. A. , Apostolaki, E. T. , Atwood, T. B. , Baldock, J. , Bianchi, T. S. , Chmura, G. L. , Eyre, B. D. , Fourqurean, J. W. , … Duarte, C. M. (2019). The future of blue carbon science. Nature Communications, 10(1), 3998. 10.1038/s41467-019-11693-w PMC6728345 31488846

[jpy70031-bib-0174] Macreadie, P. I. , Costa, M. D. P. , Atwood, T. B. , Friess, D. A. , Kelleway, J. J. , Kennedy, H. , Lovelock, C. E. , Serrano, O. , & Duarte, C. M. (2021). Blue carbon as a natural climate solution. Nature Reviews Earth and Environment, 2(12), 826–839. 10.1038/s43017-021-00224-1

[jpy70031-bib-0175] Manikandan, B. , Thomas, A. M. , Shetye, S. S. , Balamurugan, S. , Mohandass, C. , & Nandakumar, K. (2021). Macroalgal release of dissolved organic carbon in coral reef and its interaction with the bacteria associated with the coral *Porites lutea* . Environmental Science and Pollution Research, 28(47), 66998–67010. 10.1007/s11356-021-15096-7 34240306

[jpy70031-bib-0176] Marschner, B. , Brodowski, S. , Dreves, A. , Gleixner, G. , Gude, A. , Grootes, P. M. , Hamer, U. , Heim, A. , Jandl, G. , Ji, R. , Kaiser, K. , Kalbitz, K. , Kramer, C. , Leinweber, P. , Rethemeyer, J. , Schäffer, A. , Schmidt, M. W. I. , Schwark, L. , & Wiesenberg, G. L. B. (2008). How relevant is recalcitrance for the stabilization of organic matter in soils? Journal of Plant Nutrition and Soil Science, 171(1), 91–110. 10.1002/jpln.200700049

[jpy70031-bib-0177] Matear, R. J. , & Hirst, A. C. (2003). Long‐term changes in dissolved oxygen concentrations in the ocean caused by protracted global warming. Global Biogeochemical Cycles, 17(4), 1–17. 10.1029/2002GB001997

[jpy70031-bib-0178] Matsunaga, K. , Kawaguchi, T. , Suzuki, Y. , & Nigi, G. (1999). The role of terrestrial humic substances on the shift of kelp community to crustose coralline algae community of the southern Hokkaido Island in the Japan Sea. Journal of Experimental Marine Biology and Ecology, 241(2), 193–205. 10.1016/S0022-0981(99)00077-5

[jpy70031-bib-0179] McCann, M. C. , & Carpita, N. C. (2015). Biomass recalcitrance: A multi‐scale, multi‐factor, and conversion‐specific property. Journal of Experimental Botany, 66(14), 4109–4118. 10.1093/jxb/erv267 26060266

[jpy70031-bib-0180] Mcleod, E. , Chmura, G. L. , Bouillon, S. , Salm, R. , Björk, M. , Duarte, C. M. , Lovelock, C. E. , Schlesinger, W. H. , & Silliman, B. R. (2011). A blueprint for blue carbon: Toward an improved understanding of the role of vegetated coastal habitats in sequestering CO_2_ . Frontiers in Ecology and the Environment, 9(10), 552–560. 10.1890/110004

[jpy70031-bib-0181] Mellbrand, K. , Lavery, P. S. , Hyndes, G. , & Hambäck, P. A. (2011). Linking land and sea: Different pathways for marine subsidies. Ecosystems, 14(5), 732–744. 10.1007/s10021-011-9442-x

[jpy70031-bib-0182] Middelburg, J. J. (1989). A simple rate model for organic matter decomposition in marine sediments. Geochimica et Cosmochimica Acta, 53(7), 1577–1581. 10.1016/0016-7037(89)90239-1

[jpy70031-bib-0183] Moreda, U. , Mazarrasa, I. , Cebrian, E. , Kaal, J. , Ricart, A. M. , Serrano, E. , & Serrano, O. (2024). Role of macroalgal forests within Mediterranean shallow bays in blue carbon storage. Science of the Total Environment, 934, 173219. 10.1016/j.scitotenv.2024.173219 38750738

[jpy70031-bib-0184] Morrison, J. M. , Murphy, C. L. , Baker, K. , Zamor, R. M. , Nikolai, S. J. , Wilder, S. , Elshahed, M. S. , & Youssef, N. H. (2017). Microbial communities mediating algal detritus turnover under anaerobic conditions. PeerJ, 5, e2803. 10.7717/peerj.2803 28097050 PMC5228501

[jpy70031-bib-0185] Nedzarek, A. , & Rakusa‐Suszczewski, S. (2004). Decomposition of macroalgae and the release of nutrient in Admiralty Bay, King George Island, Antarctica. Polar Bioscience, 17, 26–35.

[jpy70031-bib-0186] Niklas, K. J. , Cobb, E. D. , & Matas, A. J. (2017). The evolution of hydrophobic cell wall biopolymers: From algae to angiosperms. Journal of Experimental Botany, 68(19), 5261–5269. 10.1093/jxb/erx215 28666381

[jpy70031-bib-0187] Norderhaug, K. M. , Fredriksen, S. , & Nygaard, K. (2003). Trophic importance of *Laminaria hyperborea* to kelp forest consumers and the importance of bacterial degradation to food quality. Marine Ecology Progress Series, 255(June 2003), 135–144. 10.3354/meps255135

[jpy70031-bib-0188] Norderhaug, K. M. , Nygaard, K. , & Fredriksen, S. (2006). Importance of phlorotannin content and C:N ratio of *Laminaria hyperborea* in determining its palatability as food for consumers. Marine Biology Research, 2(6), 367–371. 10.1080/17451000600962789

[jpy70031-bib-0189] Nowicki, M. , DeVries, T. , & Siegel, D. A. (2022). Quantifying the carbon export and sequestration pathways of the ocean's biological carbon pump. Global Biogeochemical Cycles, 36(3), e2021GB007083. 10.1029/2021GB007083

[jpy70031-bib-0190] Oades, J. M. (1989). An introduction to organic matter in mineral soils. In B. Dixon , & S. B. Weed (Eds.), Minerals in soil environments (2nd ed., Vol. 1, pp. 89–159). John Wiley & Sons. 10.2136/sssabookser1.2ed.c3

[jpy70031-bib-0191] Ogawa, H. , Amagai, Y. , Koike, I. , Kaiser, K. , & Benner, R. (2001). Production of refractory dissolved organic matter by bacteria. Science, 292(5518), 917–920. 10.1126/science.1057627 11340202

[jpy70031-bib-0192] Olabarria, C. , Lastra, M. , & Garrido, J. (2007). Succession of macrofauna on macroalgal wrack of an exposed sandy beach: Effects of patch size and site. Marine Environmental Research, 63(1), 19–40. 10.1016/j.marenvres.2006.06.001 16890281

[jpy70031-bib-0193] Olson, A. M. , Hessing‐Lewis, M. , Haggarty, D. , & Juanes, F. (2019). Nearshore seascape connectivity enhances seagrass meadow nursery function. Ecological Applications, 29(5), e01897. 10.1002/eap.1897 31125160

[jpy70031-bib-0194] Ørberg, S. B. , Quesada, C. M. D. , Geraldi, N. , Sejr, M. K. , Wegeberg, S. , Hansen, J. L. S. , & Krause‐Jensen, D. (2023). Prevalent fingerprint of marine macroalgae in arctic surface sediments. Science of the Total Environment, 898, 165507. 10.1016/j.scitotenv.2023.165507 37442464

[jpy70031-bib-0195] Oren, A. (2007). Diversity of organic osmotic compounds and osmotic adaptation in cyanobacteria and algae. In J. Seckbach (Ed.), Algae and cyanobacteria in extreme environments (Vol. 11, pp. 639–655). Springer. 10.1007/978-1-4020-6112-7_35

[jpy70031-bib-0196] Ortega, A. , Geraldi, N. R. , Alam, I. , Kamau, A. A. , Acinas, S. G. , Logares, R. , Gasol, J. M. , Massana, R. , Krause‐Jensen, D. , & Duarte, C. M. (2019). Important contribution of macroalgae to oceanic carbon sequestration. Nature Geoscience, 12(9), 748–754. 10.1038/s41561-019-0421-8

[jpy70031-bib-0197] Ouyang, X. , Kristensen, E. , Zimmer, M. , Thornber, C. , Yang, Z. , & Lee, S. Y. (2023). Response of macrophyte litter decomposition in global blue carbon ecosystems to climate change. Global Change Biology, 29(13), 3806–3820. 10.1111/gcb.16693 36946867

[jpy70031-bib-0198] Ouyang, X. , Lee, S. Y. , & Connolly, R. M. (2017). The role of root decomposition in global mangrove and saltmarsh carbon budgets. Earth‐Science Reviews, 166, 53–63. 10.1016/j.earscirev.2017.01.004

[jpy70031-bib-0199] Paalme, T. , Kukk, H. , Kotta, J. , & Orav, H. (2002). ‘In vitro’ and ‘in situ’ decomposition of nuisance macroalgae *Cladophora glomerata* and *Pilayella littoralis* . Hydrobiologia, 475, 469–476. 10.1007/978-94-017-2464-7_36

[jpy70031-bib-0200] Paine, E. R. , Schmid, M. , Boyd, P. W. , Diaz‐Pulido, G. , & Hurd, C. L. (2021). Rate and fate of dissolved organic carbon release by seaweeds: A missing link in the coastal ocean carbon cycle. Journal of Phycology, 57(5), 1375–1391. 10.1111/jpy.13198 34287891

[jpy70031-bib-0201] Paul, V. J. , Puglisi, M. P. , & Ritson‐Williams, R. (2006). Marine chemical ecology. Natural Product Reports, 23(2), 153. 10.1039/b404735b 16572226

[jpy70031-bib-0202] Pawlowicz, R. (2013). Key physical variables in the ocean: Temperature, salinity, and density. Nature Education Knowledge, 4(4), 13. https://www.nature.com/scitable/knowledge/library/key‐physical‐variables‐in‐the‐ocean‐temperature‐102805293/

[jpy70031-bib-0203] Pedersen, M. F. , Filbee‐Dexter, K. , Frisk, N. L. , Sárossy, Z. , & Wernberg, T. (2021). Carbon sequestration potential increased by incomplete anaerobic decomposition of kelp detritus. Marine Ecology Progress Series, 660, 53–67. 10.3354/meps13613

[jpy70031-bib-0204] Pedersen, M. F. , & Johnsen, K. L. (2017). Nutrient (N and P) dynamics of the invasive macroalga *Gracilaria vermiculophylla*: Nutrient uptake kinetics and nutrient release through decomposition. Marine Biology, 164(8), 1–12. 10.1007/s00227-017-3197-7 27980349

[jpy70031-bib-0205] Pedersen, M. F. , Stæhr, P. A. , Wernberg, T. , & Thomsen, M. S. (2005). Biomass dynamics of exotic *Sargassum muticum* and native *Halidrys siliquosa* in Limfjorden, Denmark—Implications of species replacements on turnover rates. Aquatic Botany, 83(1), 31–47. 10.1016/j.aquabot.2005.05.004

[jpy70031-bib-0206] Pendleton, L. , Donato, D. C. , Murray, B. C. , Crooks, S. , Jenkins, W. A. , Sifleet, S. , Craft, C. , Fourqurean, J. W. , Kauffman, J. B. , Marbà, N. , Megonigal, P. , Pidgeon, E. , Herr, D. , Gordon, D. , & Baldera, A. (2012). Estimating global “blue carbon” emissions from conversion and degradation of vegetated coastal ecosystems. PLoS ONE, 7(9), e43542. 10.1371/journal.pone.0043542 22962585 PMC3433453

[jpy70031-bib-0207] Perkins, A. K. , Rose, A. L. , Grossart, H.‐P. , Schulz, K. G. , Neubauer, D. , Tonge, M. P. , Rosentreter, J. A. , Eyre, B. D. , Rojas‐Jimenez, K. , Deschaseaux, E. , & Oakes, J. M. (2023). Fungi increases kelp (*Ecklonia radiata*) remineralisation and dissolved organic carbon, alkalinity, and dimethylsulfoniopropionate (DMSP) production. Science of the Total Environment, 905, 166957. 10.1016/j.scitotenv.2023.166957 37704140

[jpy70031-bib-0208] Pessarrodona, A. , Franco‐Santos, R. M. , Wright, L. S. , Vanderklift, M. A. , Howard, J. , Pidgeon, E. , Wernberg, T. , & Filbee‐Dexter, K. (2023). Carbon sequestration and climate change mitigation using macroalgae: A state of knowledge review. Biological Reviews, 98, 1945–1971. 10.1111/brv.12990 37437379

[jpy70031-bib-0209] Pessarrodona, A. , Moore, P. J. , Sayer, M. D. J. , & Smale, D. A. (2018). Carbon assimilation and transfer through kelp forests in the NE Atlantic is diminished under a warmer ocean climate. Global Change Biology, 24(9), 4386–4398. 10.1111/gcb.14303 29862600 PMC6120504

[jpy70031-bib-0210] *PlotDigitizer* (Version 3.1.5) . (2024). [Computer software]. https://plotdigitizer.com

[jpy70031-bib-0211] Popper, Z. A. , Michel, G. , Hervé, C. , Domozych, D. S. , Willats, W. G. T. , Tuohy, M. G. , Kloareg, B. , & Stengel, D. B. (2011). Evolution and diversity of plant cell walls: From algae to flowering plants. Annual Review of Plant Biology, 62, 567–590. 10.1146/annurev-arplant-042110-103809 21351878

[jpy70031-bib-0212] Queirós, A. M. , Stephens, N. , Widdicombe, S. , Tait, K. , McCoy, S. J. , Ingels, J. , Rühl, S. , Airs, R. , Beesley, A. , Carnovale, G. , Cazenave, P. , Dashfield, S. , Hua, E. , Jones, M. , Lindeque, P. , McNeill, C. L. , Nunes, J. , Parry, H. , Pascoe, C. , … Somerfield, P. J. (2019). Connected macroalgal‐sediment systems: Blue carbon and food webs in the deep coastal ocean. Ecological Monographs, 89(3), 1–21. 10.1002/ecm.1366

[jpy70031-bib-0213] Queirós, A. M. , Tait, K. , Clark, J. R. , Bedington, M. , Pascoe, C. , Torres, R. , Somerfield, P. J. , & Smale, D. A. (2023). Identifying and protecting macroalgae detritus sinks toward climate change mitigation. Ecological Applications, 33(3), 1–16. 10.1002/eap.2798 36504412

[jpy70031-bib-0214] R Core Team . (2023). *R: A Language and Environment for Statistical Computing* [Computer software]. R Foundation for Statistical Computing. https://www.R‐project.org/

[jpy70031-bib-0215] Raut, Y. , & Capone, D. G. (2021). Macroalgal detrital systems: An overlooked ecological niche for heterotrophic nitrogen fixation. Environmental Microbiology, 23(8), 4372–4388. 10.1111/1462-2920.15622 34097341

[jpy70031-bib-0216] Renaud, P. E. , Løkken, T. S. , Jørgensen, L. L. , Berge, J. , & Johnson, B. J. (2015). Macroalgal detritus and food‐web subsidies along an Arctic fjord depth‐gradient. Frontiers in Marine Science, 2, 31. 10.3389/fmars.2015.00031

[jpy70031-bib-0217] Reynolds, G. T. , & Lutz, R. A. (2001). Sources of light in the deep ocean. Reviews of Geophysics, 39(1), 123–136. 10.1029/1999RG000071

[jpy70031-bib-0218] Rice, D. (1982). The detritus nitrogen problem: New observations and perspectives from organic geochemistry. Marine Ecology Progress Series, 9, 153–162. 10.3354/meps009153

[jpy70031-bib-0219] Rice, D. , & Tenore, K. R. (1981). Dynamics of carbon and nitrogen during the decomposition of detritus derived from estuarine macrophytes. Estuarine, Coastal and Shelf Science, 13(6), 681–690. 10.1016/S0302-3524(81)80049-7

[jpy70031-bib-0220] Rieper‐Kirchner, M. (1989). Microbial degradation of North Sea macroalgae: Field and laboratory studies. Botanica Marina, 32, 241–252. 10.1515/botm.1989.32.3.241

[jpy70031-bib-0221] Rieper‐Kirchner, M. (1990). Macroalgal decomposition: Laboratory studies with particular regard to microorganisms and meiofauna. Helgolander Meeresuntersuchungen, 44(3‐4), 397–410. 10.1007/BF02365476

[jpy70031-bib-0222] Robinson, J. D. , Mann, K. H. , & Novitsky, J. A. (1982). Conversion of the particulate fraction of seaweed detritus to bacterial biomass. Limnology and Oceanography, 27(6), 1072–1079. 10.4319/lo.1982.27.6.1072

[jpy70031-bib-0223] Rodil, I. F. , Olabarria, C. , Lastra, M. , & López, J. (2008). Differential effects of native and invasive algal wrack on macrofaunal assemblages inhabiting exposed sandy beaches. Journal of Experimental Marine Biology and Ecology, 358(1), 1–13. 10.1016/j.jembe.2007.12.030

[jpy70031-bib-0224] Rogers‐Bennett, L. , & Catton, C. A. (2019). Marine heat wave and multiple stressors tip bull kelp forest to sea urchin barrens. Scientific Reports, 9(1), 15050. 10.1038/s41598-019-51114-y 31636286 PMC6803666

[jpy70031-bib-0225] Rossi, F. (2006). Small‐scale burial of macroalgal detritus in marine sediments: Effects of *Ulva* spp. on the spatial distribution of macrofauna assemblages. Journal of Experimental Marine Biology and Ecology, 332(1), 84–95. 10.1016/j.jembe.2005.11.003

[jpy70031-bib-0226] Rothäusler, E. , Reinwald, H. , López, B. A. , Tala, F. , & Thiel, M. (2018). High acclimation potential in floating *Macrocystis pyrifera* to abiotic conditions even under grazing pressure – A field study. Journal of Phycology, 54(3), 368–379. 10.1111/jpy.12643 29533462

[jpy70031-bib-0227] Roussel, J.‐M. , Paillisson, J.‐M. , Tréguier, A. , & Petit, E. (2015). The downside of eDNA as a survey tool in water bodies. Journal of Applied Ecology, 52(4), 823–826.

[jpy70031-bib-0228] Safak, I. , Wiberg, P. L. , Richardson, D. L. , & Kurum, M. O. (2015). Controls on residence time and exchange in a system of shallow coastal bays. Continental Shelf Research, 97, 7–20. 10.1016/j.csr.2015.01.009

[jpy70031-bib-0229] Sakai, T. , Kimura, H. , & Kato, I. (2002). A marine strain of Flavobacteriaceae utilizes brown seaweed fucoidan. Marine Biotechnology, 4(4), 399–405. 10.1007/s10126-002-0032-y 14961251

[jpy70031-bib-0230] Salathe, R. , & Riera, P. (2012). The role of *Talitrus saltator* in the decomposition of seaweed wrack on sandy beaches in northern Brittany: An experimental mesocosm approach. Cahiers de Biologie Marine, 53, 517–524.

[jpy70031-bib-0231] Salovius, S. , & Bonsdorff, E. (2004). Effects of depth, sediment and grazers on the degradation of drifting filamentous algae (*Cladophora glomerata* and *Pilayella littoralis*). Journal of Experimental Marine Biology and Ecology, 298(1), 93–109. 10.1016/j.jembe.2003.08.006

[jpy70031-bib-0232] Schimani, K. , Zacher, K. , Jerosch, K. , Pehlke, H. , Wiencke, C. , & Bartsch, I. (2022). Video survey of deep benthic macroalgae and macroalgal detritus along a glacial Arctic fjord: Kongsfjorden (Spitsbergen). Polar Biology, 45(7), 1291–1305. 10.1007/s00300-022-03072-x

[jpy70031-bib-0233] Schmidt, M. W. I. , Torn, M. S. , Abiven, S. , Dittmar, T. , Guggenberger, G. , Janssens, I. A. , Kleber, M. , Kögel‐Knabner, I. , Lehmann, J. , Manning, D. A. C. , Nannipieri, P. , Rasse, D. P. , Weiner, S. , & Trumbore, S. E. (2011). Persistence of soil organic matter as an ecosystem property. Nature, 478(7367), 49–56. 10.1038/nature10386 21979045

[jpy70031-bib-0234] Seebens, H. , Blackburn, T. M. , Dyer, E. E. , Genovesi, P. , Hulme, P. E. , Jeschke, J. M. , Pagad, S. , Pyšek, P. , Winter, M. , Arianoutsou, M. , Bacher, S. , Blasius, B. , Brundu, G. , Capinha, C. , Celesti‐Grapow, L. , Dawson, W. , Dullinger, S. , Fuentes, N. , Jäger, H. , … Essl, F. (2017). No saturation in the accumulation of alien species worldwide. Nature Communications, 8(1), 14435. 10.1038/ncomms14435 PMC5316856 28198420

[jpy70031-bib-0235] Shank, G. C. , Zepp, R. G. , Vähätalo, A. , Lee, R. , & Bartels, E. (2010). Photobleaching kinetics of chromophoric dissolved organic matter derived from mangrove leaf litter and floating *Sargassum* colonies. Marine Chemistry, 119(1–4), 162–171. 10.1016/j.marchem.2010.01.003

[jpy70031-bib-0236] Shen, Y. , & Benner, R. (2018). Mixing it up in the ocean carbon cycle and the removal of refractory dissolved organic carbon. Scientific Reports, 8(1), 2542. 10.1038/s41598-018-20857-5 29416076 PMC5803198

[jpy70031-bib-0237] Shtein, I. , Bar‐On, B. , & Popper, Z. A. (2018). Plant and algal structure: From cell walls to biomechanical function. Physiologia Plantarum, 164(1), 56–66. 10.1111/ppl.12727 29572853

[jpy70031-bib-0238] Shukla, P. S. , Borza, T. , Critchley, A. T. , & Prithiviraj, B. (2016). Carrageenans from red seaweeds as promoters of growth and elicitors of defense response in plants. Frontiers in Marine Science, 3, 81. 10.3389/fmars.2016.00081

[jpy70031-bib-0239] Sichert, A. , Corzett, C. H. , Schechter, M. S. , Unfried, F. , Markert, S. , Becher, D. , Fernandez‐Guerra, A. , Liebeke, M. , Schweder, T. , Polz, M. F. , & Hehemann, J. H. (2020). Verrucomicrobia use hundreds of enzymes to digest the algal polysaccharide fucoidan. Nature Microbiology, 5(8), 1026–1039. 10.1038/s41564-020-0720-2 32451471

[jpy70031-bib-0240] Siegel, D. A. , DeVries, T. , Cetinić, I. , & Bisson, K. M. (2023). Quantifying the ocean's biological pump and its carbon cycle impacts on global scales. Annual Review of Marine Science, 15, 329–356. 10.1146/annurev-marine-040722-115226 36070554

[jpy70031-bib-0241] Simpson, L. T. , Chapman, S. K. , Simpson, L. M. , & Cherry, J. A. (2023). Do global change variables alter mangrove decomposition? A systematic review. Global Ecology and Biogeography, 32(11), 1874–1892. 10.1111/geb.13743

[jpy70031-bib-0242] Smale, D. A. , Pessarrodona, A. , King, N. , & Moore, P. J. (2022). Examining the production, export, and immediate fate of kelp detritus on open‐coast subtidal reefs in the Northeast Atlantic. Limnology and Oceanography, 67(S2), S36–S49. 10.1002/lno.11970

[jpy70031-bib-0243] Smeaton, C. , Austin, W. E. N. , Davies, A. L. , Baltzer, A. , Howe, J. A. , & Baxter, J. M. (2017). Scotland's forgotten carbon: A national assessment of mid‐latitude fjord sedimentary carbon stocks. Biogeosciences, 14(24), 5663–5674. 10.5194/bg-14-5663-2017

[jpy70031-bib-0244] Smith, B. D. , & Foreman, R. E. (1984). An assessment of seaweed decomposition within a southern Strait of Georgia seaweed community. Marine Biology, 84(2), 197–205. 10.1007/BF00393005

[jpy70031-bib-0245] Smith, J. G. , Malone, D. , & Carr, M. H. (2024). Consequences of kelp forest ecosystem shifts and predictors of persistence through multiple stressors. Proceedings of the Royal Society B: Biological Sciences, 291(2016), 20232749. 10.1098/rspb.2023.2749 PMC10846955 38320605

[jpy70031-bib-0246] Sosik, E. , & Simenstad, C. (2013). Isotopic evidence and consequences of the role of microbes in macroalgae detritus‐based food webs. Marine Ecology Progress Series, 494, 107–119. 10.3354/meps10544

[jpy70031-bib-0247] Steneck, R. S. , & Dethier, M. N. (1994). A functional group approach to the structure of algal‐dominated communities. Oikos, 69(3), 476. 10.2307/3545860

[jpy70031-bib-0248] Sterner, R. W. , & Hessen, D. O. (1994). Algal nutrient limitation and the nutrition of aquatic herbivores. Annual Review of Ecology and Systematics, 25, 1–29.

[jpy70031-bib-0249] Stuart, V. , Lucas, M. , & Newell, R. (1981). Heterotrophic utilisation of particulate matter from the kelp *Laminaria pallida* . Marine Ecology Progress Series, 4, 337–348. 10.3354/meps004337

[jpy70031-bib-0250] Synytsya, A. , Čopíková, J. , Kim, W. J. , & Park, Y. I. (2015). Cell wall polysaccharides of marine algae. In S.‐K. Kim (Ed.), Springer handbook of marine biotechnology (pp. 543–590). Springer. 10.1007/978-3-642-53971-8_22

[jpy70031-bib-0251] Tala, F. , López, B. A. , Velásquez, M. , Jeldres, R. , Macaya, E. C. , Mansilla, A. , Ojeda, J. , & Thiel, M. (2019). Long‐term persistence of the floating bull kelp *Durvillaea* *antarctica* from the south‐East Pacific: Potential contribution to local and transoceanic connectivity. Marine Environmental Research, 149, 67–79. 10.1016/j.marenvres.2019.05.013 31154063

[jpy70031-bib-0252] Targett, N. M. , Coen, L. D. , Boettcher, A. A. , & Tanner, C. E. (1992). Biogeographic comparisons of marine algal polyphenolics: Evidence against a latitudinal trend. Oecologia, 89(4), 464–470. 10.1007/BF00317150 28311874

[jpy70031-bib-0253] Tenore, K. R. , Hanson, R. B. , Dornseif, B. E. , & Wiederhold, C. N. (1979). The effect of organic nitrogen supplement on the utilization of different sources of detritus. Limnology and Oceanography, 24(2), 350–355. 10.4319/lo.1979.24.2.0350

[jpy70031-bib-0254] Thomson, A. , Kristensen, E. , Valdemarsen, T. , & Quintana, C. (2020). Short‐term fate of seagrass and macroalgal detritus in *Arenicola* *marina* bioturbated sediments. Marine Ecology Progress Series, 639, 21–35. 10.3354/meps13281

[jpy70031-bib-0255] Trevathan‐Tackett, S. M. , Jeffries, T. C. , Macreadie, P. I. , Manojlovic, B. , & Ralph, P. (2020). Long‐term decomposition captures key steps in microbial breakdown of seagrass litter. Science of the Total Environment, 705, 135806. 10.1016/j.scitotenv.2019.135806 31838420

[jpy70031-bib-0256] Trevathan‐Tackett, S. M. , Kelleway, J. , Macreadie, P. I. , Beardall, J. , Ralph, P. , & Bellgrove, A. (2015). Comparison of marine macrophytes for their contributions to blue carbon sequestration. Ecology, 96(11), 3043–3057. 10.1890/15-0149.1 27070023

[jpy70031-bib-0257] Twilley, R. R. , Ejdung, G. , Romare, P. , & Kemp, W. M. (1986). A comparative study of decomposition, oxygen consumption and nutrient release for selected aquatic plants occurring in an estuarine environment. Oikos, 47(2), 190–198. 10.2307/3566045

[jpy70031-bib-0258] Urban‐Malinga, B. , & Burska, D. (2009). The colonization of macroalgal wrack by the meiofauna in the Arctic intertidal. Estuarine, Coastal and Shelf Science, 85(4), 666–670. 10.1016/j.ecss.2009.09.033

[jpy70031-bib-0259] Urban‐Malinga, B. , Gheskiere, T. , Degraer, S. , Derycke, S. , Opalinski, K. W. , & Moens, T. (2008). Gradients in biodiversity and macroalgal wrack decomposition rate across a macrotidal, ultradissipative sandy beach. Marine Biology, 155(1), 79–90. 10.1007/s00227-008-1009-9

[jpy70031-bib-0260] van der Heijden, L. H. , & Kamenos, N. A. (2015). Reviews and syntheses: Calculating the global contribution of coralline algae to total carbon burial. Biogeosciences, 12(21), 6429–6441. 10.5194/bg-12-6429-2015

[jpy70031-bib-0261] van Hees, D. , Olsen, Y. , Mattio, L. , Ruiz‐Montoya, L. , Wernberg, T. , & Kendrick, G. (2018). Cast adrift: Physiology and dispersal of benthic *Sargassum spinuligerum* in surface rafts: Seaweed acclimation in rafts. Limnology and Oceanography, 64, 1–15. 10.1002/lno.11057

[jpy70031-bib-0262] Verdugo, P. , Alldredge, A. L. , Azam, F. , Kirchman, D. L. , Passow, U. , & Santschi, P. H. (2004). The oceanic gel phase: A bridge in the DOM–POM continuum. Marine Chemistry, 92(1), 67–85. 10.1016/j.marchem.2004.06.017

[jpy70031-bib-0263] Vichkovitten, T. , & Holmer, M. (2004). Contribution of plant carbohydrates to sedimentary carbon mineralization. Organic Geochemistry, 35(9), 1053–1066. 10.1016/j.orggeochem.2004.04.007

[jpy70031-bib-0265] Wada, S. , Aoki, M. N. , Tsuchiya, Y. , Sato, T. , Shinagawa, H. , & Hama, T. (2007). Quantitative and qualitative analyses of dissolved organic matter released from *Ecklonia* *cava* Kjellman, in Oura Bay, Shimoda, Izu peninsula, Japan. Journal of Experimental Marine Biology and Ecology, 349(2), 344–358. 10.1016/j.jembe.2007.05.024

[jpy70031-bib-0266] Wada, S. , & Hama, T. (2013). The contribution of macroalgae to the coastal dissolved organic matter pool. Estuarine, Coastal and Shelf Science, 129, 77–85. 10.1016/j.ecss.2013.06.007

[jpy70031-bib-0267] Wada, S. , Omori, Y. , Kayamyo, Y. , Tashiro, Y. , & Hama, T. (2015). Photoreactivity of dissolved organic matter from macroalgae. Regional Studies in Marine Science, 2, 12–18. 10.1016/j.rsma.2015.08.018

[jpy70031-bib-0268] Wagner, S. , Schubotz, F. , Kaiser, K. , Hallmann, C. , Waska, H. , Rossel, P. E. , Hansman, R. , Elvert, M. , Middelburg, J. J. , Engel, A. , Blattmann, T. M. , Catalá, T. S. , Lennartz, S. T. , Gomez‐Saez, G. V. , Pantoja‐Gutiérrez, S. , Bao, R. , & Galy, V. (2020). Soothsaying DOM: A current perspective on the future of oceanic dissolved organic carbon. Frontiers in Marine Science, 7, 341. 10.3389/fmars.2020.00341

[jpy70031-bib-0269] Wang, A. , Ye, X. , Xu, X. , Yin, X. , & Xu, Y. (2018). Settling flux and origin of particulate organic carbon in a macro‐tidal semi‐enclosed embayment: Luoyuan Bay, Southeast China coast. Estuarine, Coastal and Shelf Science, 206, 38–48. 10.1016/j.ecss.2017.03.023

[jpy70031-bib-0270] Watanabe, K. , Yoshida, G. , Hori, M. , Umezawa, Y. , Moki, H. , & Kuwae, T. (2020). Macroalgal metabolism and lateral carbon flows can create significant carbon sinks. Biogeosciences, 17(9), 2425–2440. 10.5194/bg-17-2425-2020

[jpy70031-bib-0271] Wegner, C.‐E. , Richter‐Heitmann, T. , Klindworth, A. , Klockow, C. , Richter, M. , Achstetter, T. , Glöckner, F. O. , & Harder, J. (2013). Expression of sulfatases in *Rhodopirellula baltica* and the diversity of sulfatases in the genus *Rhodopirellula* . Marine Genomics, 9, 51–61. 10.1016/j.margen.2012.12.001 23273849

[jpy70031-bib-0272] Wernberg, T. , & Filbee‐Dexter, K. (2018). Grazers extend blue carbon transfer by slowing sinking speeds of kelp detritus. Scientific Reports, 8(1), 17180. 10.1038/s41598-018-34721-z 30464260 PMC6249265

[jpy70031-bib-0273] Wider, R. K. , & Lang, G. E. (1982). A critique of the analytical methods used in examining decomposition data obtained from litter bags. Ecology, 63(6), 1636–1642. 10.2307/1940104

[jpy70031-bib-0274] Williams, S. L. (1984). Decomposition of the tropical macroalga *Caulerpa cupressoides* (West) C. Agardh: Field and laboratory studies. Journal of Experimental Marine Biology and Ecology, 80(2), 109–124. 10.1016/0022-0981(84)90007-8

[jpy70031-bib-0275] Wright, L. S. , & Foggo, A. (2021). Photosynthetic pigments of co‐occurring Northeast Atlantic *Laminaria* spp. are unaffected by decomposition. Marine Ecology Progress Series, 678, 227–232. 10.3354/meps13886

[jpy70031-bib-0276] Wright, L. S. , & Kregting, L. (2023). Genus‐specific response of kelp photosynthetic pigments to decomposition. Marine Biology, 170(11), 144. 10.1007/s00227-023-04289-y

[jpy70031-bib-0277] Wright, L. S. , Pessarrodona, A. , & Foggo, A. (2022). Climate‐driven shifts in kelp forest composition reduce carbon sequestration potential. Global Change Biology, 28(18), 5514–5531. 10.1111/gcb.16299 35694894 PMC9545355

[jpy70031-bib-0278] Wright, L. S. , Simpkins, T. , Filbee‐Dexter, K. , & Wernberg, T. (2024). Temperature sensitivity of detrital photosynthesis. Annals of Botany, 133(1), 17–28. 10.1093/aob/mcad167 38142363 PMC10921823

[jpy70031-bib-0279] Xie, Y. (2020). A meta‐analysis of critique of litterbag method used in examining decomposition of leaf litters. Journal of Soils and Sediments, 20(4), 1881–1886. 10.1007/s11368-020-02572-9

[jpy70031-bib-0280] Xie, Y. , Su, J. , Shao, K. , Hu, T. , Ming, H. , Shi, T. , Wang, W. , & Fan, J. (2024). Long‐term response of the microbial community to the degradation of DOC released from *Undaria pinnatifida* . Marine Environmental Research, 194, 106313. 10.1016/j.marenvres.2023.106313 38211474

[jpy70031-bib-0281] Xing, Q. , Tosi, L. , Braga, F. , Gao, X. , & Gao, M. (2015). Interpreting the progressive eutrophication behind the world's largest macroalgal blooms with water quality and ocean color data. Natural Hazards, 78(1), 7–21. 10.1007/s11069-015-1694-x

[jpy70031-bib-0282] Yang, H. , & Janssen, B. (2001). A mono‐component model of carbon mineralization with a dynamic rate constant. European Journal of Soil Science, 51, 517–529. 10.1046/j.1365-2389.2000.00319.x

[jpy70031-bib-0283] Yang, X. , Lin, K. , Tan, L. , & Wang, J. (2021). Utilization and release of biogenic elements by macroalgae *Ulva prolifera*: A mesocosm experiment off the coast of Qingdao, China. Marine Pollution Bulletin, 170, 112612. 10.1016/j.marpolbul.2021.112612 34139585

[jpy70031-bib-0284] Yates, M. C. , Fraser, D. J. , & Derry, A. M. (2019). Meta‐analysis supports further refinement of eDNA for monitoring aquatic species‐specific abundance in nature. Environmental DNA, 1(1), 5–13. 10.1002/edn3.7

[jpy70031-bib-0285] Zhang, L. , Li, X. , Zhang, X. , Li, Y. , & Wang, L. (2021). Bacterial alginate metabolism: An important pathway for bioconversion of brown algae. Biotechnology for Biofuels, 14(1), 158. 10.1186/s13068-021-02007-8 34275475 PMC8286568

[jpy70031-bib-0286] Zhang, L. , Liao, W. , Huang, Y. , Wen, Y. , Chu, Y. , & Zhao, C. (2022). Global seaweed farming and processing in the past 20 years. Food Production, Processing and Nutrition, 4(1), 23. 10.1186/s43014-022-00103-2

[jpy70031-bib-0287] Zhang, M. , Qin, H. , Wang, Z. , Li, B. , & Ma, Y. (2022). The interaction between DOC released by cultured kelp (*Saccharina japonica*) and the bacterial community reveals the potential for increasing marine carbon sequestration by macroalgae culture. Frontiers in Marine Science, 9, 985548. 10.3389/fmars.2022.985548

[jpy70031-bib-0288] Zhang, Y.‐S. , Zhang, Y.‐Q. , Zhao, X.‐M. , Liu, X.‐L. , Qin, Q.‐L. , Liu, N.‐H. , Xu, F. , Chen, X.‐L. , Zhang, Y.‐Z. , & Li, P.‐Y. (2024). Metagenomic insights into the dynamic degradation of brown algal polysaccharides by kelp‐associated microbiota. Applied and Environmental Microbiology, 90(2), e02025‐23. 10.1128/aem.02025-23 38259074 PMC10880675

[jpy70031-bib-0289] Zhang, Z. , Wu, Y. , & Zhang, X.‐H. (2018). Cultivation of microbes from the deep‐sea environments. Deep Sea Research Part II: Topical Studies in Oceanography, 155, 34–43. 10.1016/j.dsr2.2017.07.008

[jpy70031-bib-0290] Zielinski, K. (1981). Benthic macroalgae of Admiralty Bay (King George Island, South Shetland Islands) and circulation of algal matter between the water and the shore. Polish Polar Research, 2(3–4), 71–94.

