# Supplementary material for: A systematic review of marine macroalgal degradation: Toward a better understanding of macroalgal carbon sequestration potential
Source: J Phycol. 2025 May 27;61(3):399–432. doi: 10.1111/jpy.70031 (PMC12168113; doi:10.1111/jpy.70031)
Supplement: Supplementary file 1 — Figure S1. PRISMA (Preferred Reporting Items for Systematic Reviews and Meta‐Analyses) flow diagram for this systematic review. Figure S2. Example degradation trajectory that exhibits a three‐parameter exponential decay relationship (black circles). If the experimental duration is confined to the initial decay period (e.g., red triangles), the degradation trajectory may appear linear, and recalcitrance is challenging to estimate. Longer experiment durations are beneficial as it enables us to understand the longevity of macroalgal biomaterial. Figure S3. Violin plots demonstrating how macroalgal half‐life (log10 transformed) varies across macroalgal functional groups (a) and macroalgal classification (b). Neither class nor functional group was a significant predictor of half‐life. Table S1. Details of the systematic search conducted by the authors including details on who conducted searches and when and the exact search terms used for each database. Table S2. Global mean values for all macroalgal degradation studies included in this review. Decay constant values were only extracted from models that were best described by exponential decay curves and degradation rate values were only estimated from data described by linear models. Data are presented as mean ± standard error, sample size refers to the number of individual observations. Table S3. To analyze the impacts of various factors on macroalgal half‐life we used a multifactor linear mixed effects model, with study included as a random effect. The model formula was: half‐life ~ functional group + class + experimental temperature + degradation environment + litterbag mesh size + light availability + pretreatment + (1|reference). Both half‐life and litterbag mesh size data were log‐transformed. The final row refers to the significance of the random effect of study. Table S4. To analyze the impacts of various factors on macroalgal recalcitrance (%) we used a multifactor generalized linear mixed effects model with a z [file JPY-61-399-s001.docx]

**Supplemental Materials for:**

**A systematic review of marine macroalgal degradation: Towards a better understanding of macroalgal carbon sequestration potential**

Jessica R. Kennedy, Caitlin O. Blain

**Link to Supplemental Datafiles 1-6:**

https://osf.io/vmtfd/files/osfstorage

DOI: 10.17605/OSF.IO/VMTFD

**Figure S1.** PRISMA (Preferred Reporting Items for Systematic Reviews and Meta-Analyses) flow diagram for this systematic review.

**
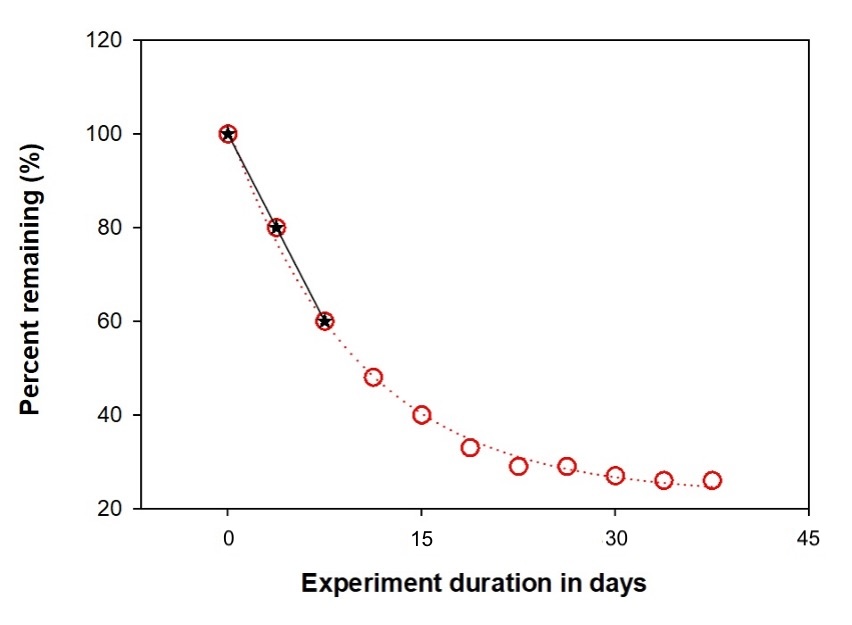
**

**Figure S2.** Example degradation trajectory that exhibits a three-parameter exponential decay relationship (black circles). If the experimental duration is confined to the initial decay period (e.g., red triangles), the degradation trajectory may appear linear, and recalcitrance is challenging to estimate. Longer experiment durations are beneficial as it enables us to understand the longevity of macroalgal biomaterial.

**Table S1.** Details of the systematic search conducted by the authors including details on who conducted searches and when and the exact search terms used for each database.

| **Search engine** | **Search number** | **Papers returned** | **Papers reviewed** | **Final Search Date** | **Reviewer** | **Exact search terms** |
| --- | --- | --- | --- | --- | --- | --- |
| Web of Science Core Collection (initial search) | 1 | 773 | 355 | 15/09/2022 | C.B. | (TS=(kelp OR fucoid)) AND TS=(carbon OR detritus) |
| Web of Science Core Collection | 1 | 347 | 347 | 08/08/2024 | J.K. | (macroalga* OR seaweed OR kelp OR fucoid) AND (detrit* OR litter OR wrack OR POM OR DOM OR POC OR DOC) AND (decompos* OR degrad* OR decay) (All Fields) |
| Scopus | 2 | 271 | 271 | 08/08/2024 | J.K. | TITLE-ABS-KEY (macroalga* OR seaweed OR kelp OR fucoid AND detrit* OR litter OR wrack OR pom OR dom OR poc OR doc AND decompos* OR degrad* OR decay ) AND ( LIMIT-TO ( PUBSTAGE , "final" ) ) AND ( LIMIT-TO ( DOCTYPE , "ar" ) ) AND ( LIMIT-TO ( LANGUAGE , "English" ) ) |
| Google Scholar (supplemental search) | 3 | 11,600 | First 500 results | 08/08/2024 | J.K. | macroalga* OR seaweed OR kelp OR fucoid AND POM OR DOM OR POC OR DOC OR detritus OR detrital OR wrack OR litter AND decompos* OR degrad* OR decay |

**Table S2**. Global mean values for all macroalgal degradation studies included in this review. Decay constant values were only extracted from models that were best described by exponential decay curves and degradation rate values were only estimated from data described by linear models. Data are presented as mean ± standard error, sample size refers to the number of individual observations.

|  | **Mean ± Standard error** | **Sample size** |
| --- | --- | --- |
| Half-life (in days) | 50.79 ± 5.5 | 426 |
| Percent recalcitrance (%) | 20.44 ± 1.66 | 235 |
| Decay constant (k value) | 0.195 ± 0.0182 | 275 |
| Degradation rate (% loss per day) | 1.83 ± 0.134 | 197 |

**Table S3.** To analyze the impacts of various factors on macroalgal half-life we used a multifactor linear mixed effects model, with study included as a random effect. The model formula was: half-life ~ functional group + class + experimental temperature + degradation environment + litterbag mesh size + light availability + pretreatment + (1|reference). Both half-life and litterbag mesh size data were log-transformed. The final row refers to the significance of the random effect of study.

|  | **Estimate** | **Standard error** | ***df*** | ***t* value** | ***p* value** |
| --- | --- | --- | --- | --- | --- |
| (Intercept) | 4.566 | 0.7232 | 59 | 6.313 | **<0.00001** |
| Experimental temperature (°C) | -0.07799 | 0.01849 | 145 | -4.211 | **<0.00001** |
| Litterbag mesh size (mm) | 0.3671 | 0.1782 | 146 | 2.06 | **0.0411** |
| Lit - Dark | 0.3240 | 0.3896 | 93 | 0.832 | 3 |
| Pretreated - Not pretreated | -0.3310 | 0.3561 | 94 | -0.93 | 0.3549 |
| **Functional group contrasts** | **Contrast** | **Standard error** | ***df*** | ***t* ratio** | ***p* value** |
| Foliose - Fucoid | 0.5398 | 0.541 | 159 | 0.998 | 0.7509 |
| Foliose - Kelp | 0.9789 | 0.458 | 159 | 2.138 | 0.1456 |
| Foliose - Turf | 0.0459 | 0.344 | 157 | 0.133 | 0.9991 |
| Fucoid - Kelp | 0.4392 | 0.401 | 156 | 1.094 | 0.6937 |
| Fucoid - Turf | -0.4939 | 0.547 | 155 | -0.903 | 0.8032 |
| Kelp - Turf | -0.9330 | 0.493 | 148 | -1.892 | 0.2360 |
| **Class contrasts** | **Contrast** | **Standard error** | ***df*** | ***t* ratio** | ***p* value** |
| Brown algae - Green algae | 0.128 | 0.359 | 147 | 0.358 | 0.9319 |
| Brown algae - Red algae | 0.631 | 0.405 | 159 | 1.556 | 0.2679 |
| Green algae - Red algae | 0.502 | 0.335 | 155 | 1.498 | 0.2946 |
| **Degradation environment contrasts** | **Contrast** | **Standard error** | ***df*** | ***t* ratio** | ***p* value** |
| Intertidal - Lab | -0.631 | 0.608 | 36 | -1.037 | 0.7290 |
| Intertidal - Seafloor | 0.325 | 0.616 | 36 | 0.528 | 0.9518 |
| Intertidal - Suspended | 0.476 | 0.692 | 46 | 0.689 | 0.9008 |
| Lab - Seafloor | 0.956 | 0.429 | 98 | 2.230 | 0.1224 |
| Lab - Suspended | 1.107 | 0.478 | 125 | 2.317 | 0.0997 |
| Seafloor - Suspended | 0.151 | 0.410 | 124 | 0.369 | 0.9827 |
| (1\|Study) |  |  | 1 |  | **<0.00001** |

**Table S4.** To analyze the impacts of various factors on macroalgal recalcitrance (%) we used a multifactor generalized linear mixed effects model with a zero-inflated negative binomial distribution, and study included as a random effect. The model formula was: precent recalcitrance (%) ~ functional group + class + experimental temperature + experiment duration + degradation environment + litterbag mesh size + light availability + pretreatment + (1|reference). Litterbag mesh size data were log-transformed. The final row refers to the significance of the random effect of study.

|  | **Estimate** | **Standard error** | ***z* value** | ***p* value** |
| --- | --- | --- | --- | --- |
| (Intercept) | 2.913 | 0.6842 | 4.257 | **<0.00001** |
| Experimental temperature (°C) | 0.01770 | 0.01904 | 0.929 | 0.3528 |
| Litterbag mesh size (mm) | 0.4403 | 0.2495 | 1.765 | 0.07758 |
| Experiment duration (days) | -0.0009864 | 0.001171 | -0.843 | 0.3993 |
| Lit - Dark | -0.2112 | 0.4214 | -0.501 | 0.6163 |
| Pretreated - Not pretreated | 0.8638 | 0.3198 | 2.701 | **0.00691** |
| **Functional group contrasts** | **Estimate** | **Standard error** | ***z* ratio** | ***p* value** |
| Foliose - Fucoid | -0.697 | 0.499 | -1.397 | 0.501 |
| Foliose - Kelp | -0.0974 | 0.487 | -0.2 | 0.9972 |
| Foliose - Turf | -0.5924 | 0.311 | -1.904 | 0.2265 |
| Fucoid - Kelp | 0.5996 | 0.262 | 2.289 | 0.1004 |
| Fucoid - Turf | 0.1046 | 0.412 | 0.254 | 0.9943 |
| Kelp - Turf | -0.4949 | 0.402 | -1.233 | 0.6061 |
| **Class contrasts** | **Estimate** | **Standard error** | ***z* ratio** | ***p* value** |
| Brown algae - Green algae | 0.564 | 0.362 | 1.558 | 0.2639 |
| Brown algae - Red algae | 0.95 | 0.395 | 2.402 | **0.043** |
| Green algae - Red algae | 0.386 | 0.43 | 0.897 | 0.6424 |
| **Degradation habitat contrasts** | **Estimate** | **Standard error** | ***z* ratio** | ***p* value** |
| Intertidal - Lab | 0.0837 | 0.392 | 0.213 | 0.9966 |
| Intertidal - Seafloor | 0.525 | 0.363 | 1.447 | 0.4696 |
| Intertidal - Suspended | -0.2281 | 0.566 | -0.403 | 0.9778 |
| Lab - Seafloor | 0.4413 | 0.313 | 1.41 | 0.4932 |
| Lab - Suspended | -0.3118 | 0.539 | -0.578 | 0.9386 |
| Seafloor - Suspended | -0.7531 | 0.583 | -1.293 | 0.5675 |
| (1\|Study) |  |  |  | 1 |

**
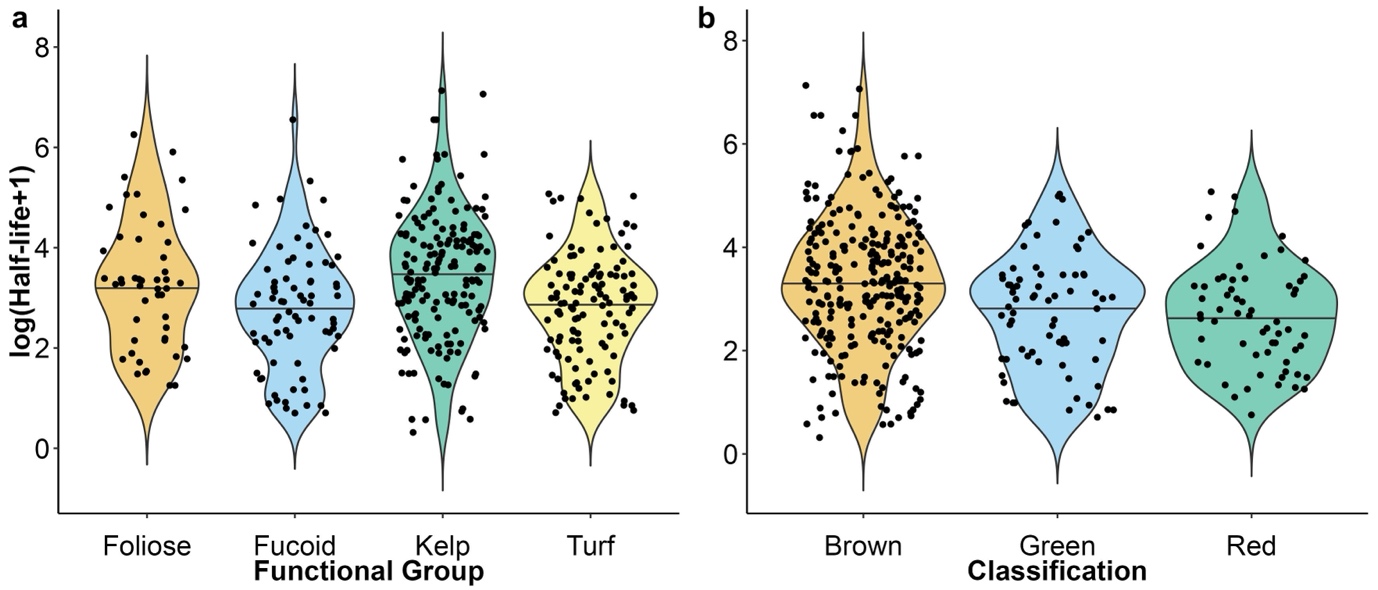
**

**Figure S3.** Violin plots demonstrating how macroalgal half-life (log_10_ transformed) varies across macroalgal functional groups (a) and macroalgal classification (b). Neither class nor functional group was a significant predictor of half-life.

**Table S5.** Findings of studies which compared how algae from different functional groups degrade. In the half-life column, species are listed in order of shortest to longest half-life, in days. In the recalcitrance column, species are listed in order of smallest to biggest portion of percent recalcitrant material. Comparisons are based on this review’s independent estimates of half-life and percent recalcitrance. Turf algal species are in green text, kelps are brown, foliose are black, fucoids are blue. Asterisks denote studies which did not test if the difference in degradation dynamics were statistically significant.

| **Half-life (in days) comparison** | **Percent recalcitrance comparison** | **Citation** |
| --- | --- | --- |
| *Undaria pinnatifida* (3.49) *Grateloupia* spp. (3.65)  *Ulva* spp. (7.7) | *Ulva* spp. (2.04%)  *Undaria pinnatifida* (5.81%)  *Grateloupia* spp. (22.54%) | (Hwang et al., 2023) |
| *Fucus vesiculosus* (17.04)  *Corallina officinalis* (19.14)  *Ulva intestinalis* (21.53) | Insufficient data for all species | (Kim et al., 2021) |
| *Ulva compressa* (1.34)  *Ulva rigida* (1.74)  *Gracilaria vermiculophylla* (2.00) *Agardhiella subulata* (2.61)  *Fucus vesiculosus* (7.97) | Insufficient data for all species | (Conover et al., 2016) |
| *Gracilaria tikvahiae* (2.8)  *Fucus vesiculosis* (9.88)  *Ulva lactuca* (13.60) | *Gracilaria tikvahiae* (0%) = *Ulva lactuca* (0%)  *Fucus vesiculosis* (16%) | (Buchsbaum et al., 1991)* |

**Table S6.** A summary of the findings of prior studies which tested how a variety of discrete methodological and environmental factors impact macroalgal degradation dynamics. Asterisks denote studies which did not verify statistically whether the factor effected degradation.

| **Factor** | **Species** | **Levels** | **Finding** | **Study** |
| --- | --- | --- | --- | --- |
| **Submersion** | *Himantothallus grandifolius* and *Leptosomia simplex* | Placed intertidally or submerged | Degradation rate was quicker when placed intertidally (only true for *H. grandifolius*, not *L. simplex*)* | (Zielinski, 1981) |
|  | *Ascophyllum nodosum* and *Fucus vesiculosus* | Placed intertidally at the wrack line or submerged | “Decomposition under continuously submerged conditions was faster than on the marsh surface” | (Josselyn & Mathieson, 1980) |
| **Sediment type** | *Fucus vesiculosus* | Salt marsh (soft sediment), or rocky shore | Decay was slightly quicker on the rocky shore, but detritus from the soft sediment salt marsh had a refractory portion while the rocky shore didn’t* | (Hunter, 1976) |
| **Sediment contact** | *Fucus serratus* | Buried under sediment or placed on sediment surface | Without grazers present, detritus degraded slower when buried, but with addition of grazer there is no difference between buried vs. not* | (Kristensen & Mikkelsen, 2003) |
|  | *Saccharina latissima* and *Alaria esculenta* | Buried >0.5 cm under sediment surface or placed on sediment surface | Degradation was faster on the surface, compared to sub-surface | (Boldreel et al., 2023) |
|  | *Agarophyton vermiculophyllum* | Buried under sediment or placed on sediment surface | Buried detritus decomposed more quickly than detritus placed on the sediment surface | (Haram et al., 2020) |
|  | *Cladophora glomerata* | Detritus bags kept in the water column or placed on the seafloor | Detritus degraded significantly faster when in contact with the sediment | (Salovius & Bonsdorff, 2004) |
|  | *Caulerpa cupressoides* | Detritus bags kept in the water column or placed on the seafloor | Detritus degraded faster when in contact with the sediment | (Williams, 1984) |
|  | *Gracilaria lemaneiformis* | Tank with just seawater, or tank with sediment and seawater | No difference | (Luo et al., 2021) |
| **Mesh diameter of litterbag** | *Saccharina latissima* | 1 mm or 50 mm | Algae in 1 mm mesh bags degraded faster | (Bedford & Moore, 1984) |
|  | *Laminaria digitata, Laminaria hyperborea,* and *Saccharina latissima* | 2 mm or 15 mm | No discernible impact* | (Wright & Kregting, 2023) |
|  | *Ulva* sp. | 80 µm, 1 mm or 5 mm | No difference | (Catenazzi & Donnelly, 2007) |
| **Pre-treatment of detritus** | *Laminaria hyperborea* | Old blades or new blades | Old blades degraded faster than new ones | (de Bettignies et al., 2020) |
|  | *Gracilaria lemaneiformis* | Oven dried or not dried | Oven dried algae degraded faster compared to fresh algae | (Luo, Xie, et al., 2022) |
|  | *Fucus serratus* | Pre-decomposed for 55 days then frozen or un-treated | Pre-decomposed algae decayed **slower**, compared to freshly frozen algae | (Kristensen & Mikkelsen, 2003) |
|  | *Gracilaria lamniformis* | Air dried for 3 days or not dried | Similar degradation trajectories, although un-treated algae had significantly less biomass left after 50 days | (Hu et al., 2023) |
|  | *Gracilaria lemaneiformis* and *Gracilaria lichenoidies* | Oven dried or frozen | No discernible impact* | (Luo, Dai, et al., 2022) |
|  | *Saccharina latissima* and *Alaria esculenta* | Frozen or not frozen | No discernible impact* | (Boldreel et al., 2023) |

**Table S7.** Comparison of the results of our review and the findings other comprehensive reviews on marine biomaterial decomposition. Data are presented as mean ± standard error *or* as mean (5th percentile-95th percentile) *or* as just the mean value. N.S. stands for not specified.

| **Detritus Source** | **Detritus Type** | **Mean decay constant or k value** | **Mean degradation rate (% loss ⋅ day^-1^)** | **Sample Size** | **Citation** |
| --- | --- | --- | --- | --- | --- |
| Mangroves | Leaves | 0.009 ± 0.0005 |  | 327 | (Simpson et al., 2023) |
|  | Wood | 0.001 ± 0.0003 |  | 70 | (Simpson et al., 2023) |
|  | Roots | 0.002 ± 0.0001 |  | 69 | (Simpson et al., 2023) |
|  |  | 0.015 ± 0.001 |  | 494 | (Ouyang et al., 2023) |
|  | Roots |  | 0.15 (0.076-0.262) | 110 | (Ouyang et al., 2017) |
|  | Roots | Oxic: 0.0007  Anoxic: 0.0004 |  | N.S. | (Lovelock et al., 2017) |
|  | Leaves | Oxic: 0.06  Anoxic: 0.03 |  | N.S. | (Lovelock et al., 2017) |
|  | Wood | Oxic: 0.0007  Anoxic: 0.0004 |  | N.S. | (Lovelock et al., 2017) |
| Salt marsh | Roots |  | 0.12 (0.052 -0.278) | 66 | (Ouyang et al., 2017) |
|  | Roots | Oxic: 0.007  Anoxic: 0.0032 |  | N.S. | (Lovelock et al., 2017) |
|  | Above ground biomass | Oxic: 0.01  Anoxic: 0.005 |  | N.S. | (Lovelock et al., 2017) |
|  |  | 0.006 ± 0.0005 |  | 325 | (Ouyang et al., 2023) |
| Seagrasses | Above ground biomass | Oxic: 0.02  Anoxic: 0.01 |  | N.S. | (Lovelock et al., 2017) |
|  | Roots | Oxic: 0.002  Anoxic: 0.001 |  | N.S. | (Lovelock et al., 2017) |
|  |  | 0.016 ± 0.001 |  | 146 | (Ouyang et al., 2023) |
| DOC* |  | 0.066 ± 0.065 |  | 127 | (Lønborg & Álvarez-Salgado, 2012) |
| Macroalgae |  | **0.195 ± 0.0182** |  | 275 | This review |
|  |  |  | **1.83 ± 0.134** | 197 | This review |

**Table 8.** Linear mixed effects model analyzing the effect of carbon and nitrogen content on macroalgal half-life. Formula: Half-life ~ Initial C:N ratio + Initial percent carbon + Initial percent nitrogen + (1|Study).

|  | ***DF*** | ***F* value** | ***p* value** | ***n*** |
| --- | --- | --- | --- | --- |
| Initial C:N ratio | 1 | 0.2052 | 0.6598 | 170 |
| Initial percent carbon | 1 | 0.0570 | 0.8157 | 57 |
| Initial percent nitrogen | 1 | 0.6935 | 0.4320 | 87 |
| (1\|Reference) | 1 |  | 0.7199 |  |

**References**

Bedford, A. P., & Moore, P. G. (1984). Macrofaunal involvement in the sublittoral decay of kelp debris: The detritivore community and species interactions. *Estuarine, Coastal and Shelf Science*, *18*(1), 97–111. https://doi.org/10.1016/0272-7714(84)90009-X

Boldreel, E., Attard, K., Hancke, K., & Glud, R. (2023). Microbial degradation dynamics of farmed kelp deposits from *Saccharina latissima* and *Alaria esculenta*. *Marine Ecology Progress Series*, *709*, 1–15. https://doi.org/10.3354/meps14285

Buchsbaum, R., Valiela, I., Swain, T., Dzierzeski, M., & Allen, S. (1991). Available and refractory nitrogen in detritus of coastal vascular plants and macroalgae. *Marine Ecology Progress Series*, *72*, 131–143. https://doi.org/10.3354/meps072131

Catenazzi, A., & Donnelly, M. A. (2007). Role of supratidal invertebrates in the decomposition of beach-cast green algae *Ulva* sp. *Marine Ecology Progress Series*, *349*, 33–42. https://doi.org/10.3354/meps07106

Conover, J., Green, L. A., & Thornber, C. S. (2016). Biomass decay rates and tissue nutrient loss in bloom and non-bloom-forming macroalgal species. *Estuarine, Coastal and Shelf Science*, *178*, 58–64. https://doi.org/10.1016/j.ecss.2016.05.018

de Bettignies, F., Dauby, P., Thomas, F., Gobet, A., Delage, L., Bohner, O., Loisel, S., & Davoult, D. (2020). Degradation dynamics and processes associated with the accumulation of *Laminaria hyperborea* (Phaeophyceae) kelp fragments: An in situ experimental approach. *Journal of Phycology*, *56*(6), 1481–1492. https://doi.org/10.1111/jpy.13041

Hanisak, M. D. (1993). Nitrogen release from decomposing seaweeds: Species and temperature effects. *Journal of Applied Phycology*, *5*(2), 175–181. https://doi.org/10.1007/BF00004014

Haram, L., Sotka, E., & Byers, J. (2020). Effects of novel, non-native detritus on decomposition and invertebrate community assemblage. *Marine Ecology Progress Series*, *643*, 49–61. https://doi.org/10.3354/meps13335

Hu, X., Cao, Y., Zhao, X., Su, H., Wen, G., & Yang, Y. (2023). Effect of bacterial community succession on environmental factors during litter decomposition of the seaweed *Gracilaria lemaneiformis*. *Marine Pollution Bulletin*, *197*, 115797. https://doi.org/10.1016/j.marpolbul.2023.115797

Hunter, R. D. (1976). Changes in carbon and nitrogen content during decomposition of three macrophytes in freshwater and marine environments. *Hydrobiologia*, *51*, 9–128.

Hwang, J.-M., Kim, H.-G., Kim, H., Hwang, C.-H., & Oh, C.-W. (2023). Meiofaunal assemblages associated with macroalgal detritus decomposition. *Regional Studies in Marine Science*, *68*, 103285. https://doi.org/10.1016/j.rsma.2023.103285

Josselyn, M. N., & Mathieson, A. C. (1980). Seasonal influx and decomposition of autochthonous macrophyte litter in a north temperate estuary. *Hydrobiologia*, *71*(3), 197–208. https://doi.org/10.1007/BF03216236

Kim, H.-G., Hawkins, L. E., Godbold, J. A., Bohn, K., Khim, J. S., & Hawkins, S. J. (2021). The influence of the composition of algal detritus on nematode assemblages. *Regional Studies in Marine Science*, *48*, 102004. https://doi.org/10.1016/j.rsma.2021.102004

Kristensen, E., & Mikkelsen, O. (2003). Impact of the burrow-dwelling polychaete *Nereis diversicolor* on the degradation of fresh and aged macroalgal detritus in a coastal marine sediment. *Marine Ecology Progress Series*, *265*, 141–153. https://doi.org/10.3354/meps265141

Lønborg, C., & Álvarez-Salgado, X. A. (2012). Recycling versus export of bioavailable dissolved organic matter in the coastal ocean and efficiency of the continental shelf pump. *Global Biogeochemical Cycles*, *26*(3). https://doi.org/10.1029/2012GB004353

Lovelock, C. E., Fourqurean, J. W., & Morris, J. T. (2017). Modeled CO2 Emissions from Coastal Wetland Transitions to Other Land Uses: Tidal Marshes, Mangrove Forests, and Seagrass Beds. *Frontiers in Marine Science*, *4*. https://doi.org/10.3389/fmars.2017.00143

Luo, H., Dai, X., Yang, Y., & Xie, S. (2022). The evaluation of C, N, P release and contribution to the water environment during *Gracilaria* litters biomass decay. *Estuarine, Coastal and Shelf Science*, *276*, 108052. https://doi.org/10.1016/j.ecss.2022.108052

Luo, H., Wang, Q., Zhang, C., Zhang, L., & Yang, Y. (2021). Bioaccumulation and release of heavy metals during growth and decomposition of cultivated *Gracilaria lemaneiformis*. *Marine Pollution Bulletin*, *173*, 113130. https://doi.org/10.1016/j.marpolbul.2021.113130

Luo, H., Xie, S., Dai, X., Wang, Q., & Yang, Y. (2022). Biomass decomposition and heavy metal release from seaweed litter, *Gracilaria lemaneiformis*, and secondary pollution evaluation. *Journal of Environmental Management*, *310*, 114729. https://doi.org/10.1016/j.jenvman.2022.114729

Ouyang, X., Kristensen, E., Zimmer, M., Thornber, C., Yang, Z., & Lee, S. Y. (2023). Response of macrophyte litter decomposition in global blue carbon ecosystems to climate change. *Global Change Biology*, *29*(13), 3806–3820. https://doi.org/10.1111/gcb.16693

Ouyang, X., Lee, S. Y., & Connolly, R. M. (2017). The role of root decomposition in global mangrove and saltmarsh carbon budgets. *Earth-Science Reviews*, *166*, 53–63. https://doi.org/10.1016/j.earscirev.2017.01.004

Salovius, S., & Bonsdorff, E. (2004). Effects of depth, sediment and grazers on the degradation of drifting filamentous algae (*Cladophora glomerata* and *Pilayella littoralis*). *Journal of Experimental Marine Biology and Ecology*, *298*(1), 93–109. https://doi.org/10.1016/j.jembe.2003.08.006

Simpson, L. T., Chapman, S. K., Simpson, L. M., & Cherry, J. A. (2023). Do global change variables alter mangrove decomposition? A systematic review. *Global Ecology and Biogeography*, *32*(11), 1874–1892. https://doi.org/10.1111/geb.13743

Williams, S. L. (1984). Decomposition of the tropical macroalga *Caulerpa cupressoides* (West) C. Agardh: Field and laboratory studies. *Journal of Experimental Marine Biology and Ecology*, *80*(2), 109–124. https://doi.org/10.1016/0022-0981(84)90007-8

Wright, L. S., & Kregting, L. (2023). Genus-specific response of kelp photosynthetic pigments to decomposition. *Marine Biology*, *170*(11), 144. https://doi.org/10.1007/s00227-023-04289-y

Zielinski, K. (1981). Benthic macroalgae of Admiralty Bay (King George Island, South Shetland Islands) and circulation of algal matter between the water and the shore. *Polish Polar Research*, *2*(3–4), 71–94.
