# Supplementary material for: A systematic review of marine macroalgal degradation: Toward a better understanding of macroalgal carbon sequestration potential
Source: J Phycol. 2025 May 27;61(3):399–432. doi: 10.1111/jpy.70031 (PMC12168113; doi:10.1111/jpy.70031)
Supplement: Supplementary file 2 — Appendix S1. Systematic review reference list. [file JPY-61-399-s002.docx]

**Supplemental File: Systematic Review Reference List**

Full reference list of papers selected for inclusion in “A systematic review of marine macroalgal degradation: Towards a better understanding of macroalgal carbon sequestration potential” (n = 105).

Alber, M., & Valiela, I. (1994). Production of microbial organic aggregates from macrophyte‐derived dissolved organic material. *Limnology and Oceanography*, *39*(1), 37–50. <https://doi.org/10.4319/lo.1994.39.1.0037>

Alkemade, R., & Van Rijswijk, P. (1993). Path analyses of the influence of substrate composition on nematode numbers and on decomposition of stranded seaweed at an Antarctic coast. *Netherlands Journal of Sea Research*, *31*(1), 63–70. <https://doi.org/10.1016/0077-7579(93)90018-N>

Bedford, A. P., & Moore, P. G. (1984). Macrofaunal involvement in the sublittoral decay of kelp debris: The detritivore community and species interactions. *Estuarine, Coastal and Shelf Science*, *18*(1), 97–111. <https://doi.org/10.1016/0272-7714(84)90009-X>

Birch, P. B., Gabrielson, J. O., & Hamel, K. S. (1983). Decomposition of *Cladophora*. I. Field Studies in the Peel-Harvey Estuarine System, Western Australia. *Botanica Marina*, *26*(4). <https://doi.org/10.1515/botm.1983.26.4.165>

Boldreel, E., Attard, K., Hancke, K., & Glud, R. (2023). Microbial degradation dynamics of farmed kelp deposits from *Saccharina latissima* and *Alaria esculenta*. *Marine Ecology Progress Series*, *709*, 1–15. <https://doi.org/10.3354/meps14285>

Bourguès, S., Auby, I., De Wit, R., & Labourg, P. (1996). Differential anaerobic decomposition of seagrass (*Zostera noltii*) and macroalgal (*Monostroma obscurum*) biomass from Arcachon Bay (France). *Hydrobiologia*, *329*, 121–131. <https://doi.org/10.1007/BF00034552>

Brouwer, P. E. M. (1996). Decomposition in situ of the sublittoral Antarctic macroalga *Desmarestia anceps* Montagne. *Polar Biology*, *16*(2), 129–137. <https://doi.org/10.1007/BF02390433>

Buchsbaum, R., Valiela, I., Swain, T., Dzierzeski, M., & Allen, S. (1991). Available and refractory nitrogen in detritus of coastal vascular plants and macroalgae. *Marine Ecology Progress Series*, *72*, 131–143. <https://doi.org/10.3354/meps072131>

Castaldelli, G., Welsh, D. T., Flachi, G., Zucchini, G., Colombo, G., Rossi, R., & Fano, E. A. (2003). Decomposition dynamics of the bloom forming macroalga *Ulva rigida* C. Agardh determined using a 14C-carbon radio-tracer technique. *Aquatic Botany*, *75*(2), 111–122. <https://doi.org/10.1016/S0304-3770(02)00167-5>

Catenazzi, A., & Donnelly, M. A. (2007). Role of supratidal invertebrates in the decomposition of beach-cast green algae *Ulva* sp. *Marine Ecology Progress Series*, *349*, 33–42. <https://doi.org/10.3354/meps07106>

Chen, J., Li, H., Zhang, Z., He, C., Shi, Q., Jiao, N., & Zhang, Y. (2020). DOC dynamics and bacterial community succession during long-term degradation of *Ulva prolifera* and their implications for the legacy effect of green tides on refractory DOC pool in seawater. *Water Research*, *185*, 116268. <https://doi.org/10.1016/j.watres.2020.116268>

Chown, S. L. (1996). Kelp degradation by *Paractora trichosterna* (Thomson) (Diptera: Helcomyzidae) at sub-Antarctic South Georgia. *Polar Biology*, *16*(3), 171–178. <https://doi.org/10.1007/s003000050042>

Conover, J., Green, L. A., & Thornber, C. S. (2016). Biomass decay rates and tissue nutrient loss in bloom and non-bloom-forming macroalgal species. *Estuarine, Coastal and Shelf Science*, *178*, 58–64. <https://doi.org/10.1016/j.ecss.2016.05.018>

Crafford, J. E., & Scholtz, C. H. (1987). Phenology of stranded kelp degradation by the kelp fly *Paractora dreuxi mirabilis* (Helcomyzidae) at Marion island. *Polar Biology*, *7*(5), 289–294. <https://doi.org/10.1007/BF00443946>

de Bettignies, F., Dauby, P., Thomas, F., Gobet, A., Delage, L., Bohner, O., Loisel, S., & Davoult, D. (2020). Degradation dynamics and processes associated with the accumulation of *Laminaria hyperborea* (Phaeophyceae) kelp fragments: An in situ experimental approach. *Journal of Phycology*, *56*(6), 1481–1492. <https://doi.org/10.1111/jpy.13041>

Dufour, C., Probert, P., & Savage, C. (2012). Macrofaunal colonisation of stranded *Durvillaea antarctica* on a southern New Zealand exposed sandy beach. *New Zealand Journal of Marine and Freshwater Research*, *46*(3), 369–383. <https://doi.org/10.1080/00288330.2012.676557>

Duggins, D. O., Gómez-Buckley, M. C., Buckley, R. M., Lowe, A. T., Galloway, A. W. E., & Dethier, M. N. (2016). Islands in the stream: Kelp detritus as faunal magnets. *Marine Biology*, *163*(1), 17. <https://doi.org/10.1007/s00227-015-2781-y>

Feng, X., Li, H., Zhang, Z., Xiong, T., Shi, X., He, C., Shi, Q., Jiao, N., & Zhang, Y. (2022). Microbial-mediated contribution of kelp detritus to different forms of oceanic carbon sequestration. *Ecological Indicators*, *142*(July), 109186. <https://doi.org/10.1016/j.ecolind.2022.109186>

Filbee-Dexter, K., Feehan, C. J., Smale, D. A., Krumhansl, K. A., Augustine, S., de Bettignies, F., Burrows, M. T., Byrnes, J. E. K., Campbell, J., Davoult, D., Dunton, K. H., Franco, J. N., Garrido, I., Grace, S. P., Hancke, K., Johnson, L. E., Konar, B., Moore, P. J., Norderhaug, K. M., … Wernberg, T. (2022). Kelp carbon sink potential decreases with warming due to accelerating decomposition. *PLoS Biology*, *20*(8), 1–22. <https://doi.org/10.1371/journal.pbio.3001702>

Franzitta, G., Hanley, M. E., Airoldi, L., Baggini, C., Bilton, D. T., Rundle, S. D., & Thompson, R. C. (2015). Home advantage? Decomposition across the freshwater-estuarine transition zone varies with litter origin and local salinity. *Marine Environmental Research*, *110*, 1–7. <https://doi.org/10.1016/j.marenvres.2015.07.012>

Frontier, N., De Bettignies, F., Foggo, A., & Davoult, D. (2021). Sustained productivity and respiration of degrading kelp detritus in the shallow benthos: Detached or broken, but not dead. *Marine Environmental Research*, *166*, 105277. <https://doi.org/10.1016/j.marenvres.2021.105277>

Frontier, N., Mulas, M., Foggo, A., & Smale, D. A. (2022). The influence of light and temperature on detritus degradation rates for kelp species with contrasting thermal affinities. *Marine Environmental Research*, *173*(November 2021), 105529. <https://doi.org/10.1016/j.marenvres.2021.105529>

Gabrielson, J. O., Birch, P. B., & Hamel, K. S. (1983). Decomposition of *Cladophora* II. In vitro Studies of Nitrogen and Phosphorus Regeneration. *Botanica Marina*, *26*(4), 173–180. <https://doi.org/10.1515/botm.1983.26.4.173>

Gao, Y., Zhang, Y., Du, M., Lin, F., Jiang, W., Li, W., Li, F., Lv, X., Fang, J., & Jiang, Z. (2021). Dissolved organic carbon from cultured kelp *Saccharina japonica*: Production, bioavailability, and bacterial degradation rates. *Aquaculture Environment Interactions*, *13*, 101–110. <https://doi.org/10.3354/AEI00393>

Gilson, A. R., Smale, D. A., Burrows, M. T., & O’Connor, N. E. (2021). Spatio-temporal variability in the deposition of beach-cast kelp (wrack) and inter-specific differences in degradation rates. *Marine Ecology Progress Series*, *674*, 89–102. Scopus. <https://doi.org/10.3354/meps13825>

Gladstone-Gallagher, R. V., Lohrer, A. M., Lundquist, C. J., & Pilditch, C. A. (2016). Effects of detrital subsidies on soft-sediment ecosystem function are transient and source-dependent. *PLOS ONE*, *11*(5), e0154790. <https://doi.org/10.1371/journal.pone.0154790>

Gómez, M., Barreiro, F., López, J., & Lastra, M. (2018). Effect of upper beach macrofauna on nutrient cycling of sandy beaches: Metabolic rates during wrack decay. *Marine Biology*, *165*(8), 133. <https://doi.org/10.1007/s00227-018-3392-1>

Griffiths, C. L., & Stenton-Dozey, J. (1981). The fauna and rate of degradation of stranded kelp. *Estuarine, Coastal and Shelf Science*, *12*(6), 645–653. <https://doi.org/10.1016/S0302-3524(81)80062-X>

Hamersley, M. R., Sohm, J. A., Burns, J. A., & Capone, D. G. (2015). Nitrogen fixation associated with the decomposition of the giant kelp *Macrocystis pyrifera*. *Aquatic Botany*, *125*, 57–63. <https://doi.org/10.1016/j.aquabot.2015.05.003>

Hanisak, M. D. (1993). Nitrogen release from decomposing seaweeds: Species and temperature effects. *Journal of Applied Phycology*, *5*(2), 175–181. <https://doi.org/10.1007/BF00004014>

Haram, L., Sotka, E., & Byers, J. (2020). Effects of novel, non-native detritus on decomposition and invertebrate community assemblage. *Marine Ecology Progress Series*, *643*, 49–61. <https://doi.org/10.3354/meps13335>

Hollohan, B. T., Dabinett, P. E., & Gow, J. A. (1986). Bacterial succession during biodegradation of the kelp *Alaria esculenta* (L.) Greville. *Canadian Journal of Microbiology*, *32*(6), 505–512. <https://doi.org/10.1139/m86-092>

Hosen, M. J., Hossain, M., Martinez, J. G., Sumaya, N. H., Mei, Y., & Manandhar, S. (2011). Effect of bacterivorous and predatory nematodes on macroalgal detritus decomposition. *Proceedings of the Pakistan Academy of Sciences*, *48*(3), 137–142.

Hu, X., Cao, Y., Zhao, X., Su, H., Wen, G., & Yang, Y. (2023). Effect of bacterial community succession on environmental factors during litter decomposition of the seaweed *Gracilaria lemaneiformis*. *Marine Pollution Bulletin*, *197*, 115797. <https://doi.org/10.1016/j.marpolbul.2023.115797>

Hunter, R. D. (1976). Changes in carbon and nitrogen content during decomposition of three macrophytes in freshwater and marine environments. *Hydrobiologia*, *51*, 9–128.

Hwang, J.-M., Kim, H.-G., Kim, H., Hwang, C.-H., & Oh, C.-W. (2023). Meiofaunal assemblages associated with macroalgal detritus decomposition. *Regional Studies in Marine Science*, *68*, 103285. <https://doi.org/10.1016/j.rsma.2023.103285>

Inglis, G. (1989). The colonisation and degradation of stranded *Macrocystis pyrifera* (L.) C. Ag. By the macrofauna of a New Zealand sandy beach. *Journal of Experimental Marine Biology and Ecology*, *125*, 203–217.

Ishii, Y., Kokubu, H., Miyazaki, H., Borjigin, S., & Yabe, T. (2021). Laboratory studies on the biodegradation of organisms for estimating carbon storage potential in coastal aquatic ecosystems. *Coastal Engineering Journal*, *63*(3), 323–334. <https://doi.org/10.1080/21664250.2021.1917918>

Jiménez-Ramos, R., Tomas, F., Reynés, X., Romera-Castillo, C., Pérez-Lloréns, J. L., & Egea, L. G. (2022). Carbon metabolism and bioavailability of dissolved organic carbon (DOC) fluxes in seagrass communities are altered under the presence of the tropical invasive alga *Halimeda incrassata*. *Science of the Total Environment*, *839*(May). <https://doi.org/10.1016/j.scitotenv.2022.156325>

Josselyn, M. N., & Mathieson, A. C. (1980). Seasonal influx and decomposition of autochthonous macrophyte litter in a north temperate estuary. *Hydrobiologia*, *71*(3), 197–208. <https://doi.org/10.1007/BF03216236>

Kim, H.-G., Hawkins, L. E., Godbold, J. A., Bohn, K., Khim, J. S., & Hawkins, S. J. (2021). The influence of the composition of algal detritus on nematode assemblages. *Regional Studies in Marine Science*, *48*, 102004. <https://doi.org/10.1016/j.rsma.2021.102004>

King, G. M., Guist, G. G., & Lauterbach, G. E. (1985). Anaerobic degradation of carrageenan from the red macroalga *Eucheuma cottonii*. *Applied and Environmental Microbiology*, *49*(3), 588–592. <https://doi.org/10.1128/aem.49.3.588-592.1985>

Kochi, K., & Yanai, S. (2006). Decomposition of leaves in coastal brackish water and their use by the macroinvertebrate *Anisogammarus pugettensis* (Gammaridea). *Marine and Freshwater Research*, *57*(5), 545. <https://doi.org/10.1071/MF05140>

Kotta, J., Orav-Kotta, H., & Paalme, T. (2010). In situ evidence on the role of benthic invertebrates on the decomposition of drifting algal mats in a brackish water ecosystem. *2010 International Conference on Biosciences*, 139–144. <https://doi.org/10.1109/BioSciencesWorld.2010.25>

Kristensen, E., Andersen, F. Ø., & Blackburn, T. H. (1992). Effects of benthic macrofauna and temperature on degradation of macroalgal detritus: The fate of organic carbon. *Limnology and Oceanography*, *37*(7), 1404–1419. <https://doi.org/10.4319/lo.1992.37.7.1404>

Kristensen, E., & Mikkelsen, O. (2003). Impact of the burrow-dwelling polychaete *Nereis diversicolor* on the degradation of fresh and aged macroalgal detritus in a coastal marine sediment. *Marine Ecology Progress Series*, *265*, 141–153. <https://doi.org/10.3354/meps265141>

Krumhansl, K., & Scheibling, R. (2012). Detrital subsidy from subtidal kelp beds is altered by the invasive green alga *Codium fragile* ssp. *Fragile*. *Marine Ecology Progress Series*, *456*, 73–85. <https://doi.org/10.3354/meps09671>

Lanari, M., S. Copertino, M., Colling, L. A., & C. Bom, F. (2018). The impact of short-term depositions of macroalgal blooms on widgeon-grass meadows in a river-dominated estuary. *Harmful Algae*, *78*, 36–46. <https://doi.org/10.1016/j.hal.2018.07.006>

Lastra, M., López, J., & Rodil, I. F. (2018). Warming intensify CO2 flux and nutrient release from algal wrack subsidies on sandy beaches. *Global Change Biology*, *24*(8), 3766–3779. <https://doi.org/10.1111/gcb.14278>

Lastra, M., Rodil, I. F., Sánchez-Mata, A., García-Gallego, M., & Mora, J. (2014). Fate and processing of macroalgal wrack subsidies in beaches of Deception Island, Antarctic Peninsula. *Journal of Sea Research*, *88*, 1–10. <https://doi.org/10.1016/j.seares.2013.12.011>

Li, H., Feng, X., Xiong, T., He, C., Wu, W., Shi, Q., Jiao, N., & Zhang, Y. (2023). Green Tides Significantly Alter the Molecular Composition and Properties of Coastal DOC and Perform Dissolved Carbon Sequestration. *Environmental Science & Technology*, *57*(1), 770–779. <https://doi.org/10.1021/acs.est.2c05684>

Li, H., Feng, X., Xiong, T., Shao, W., Wu, W., & Zhang, Y. (2023). Particulate Organic Carbon Released during Macroalgal Growth Has Significant Carbon Sequestration Potential in the Ocean. *Environmental Science & Technology*, *57*(48), 19723–19731. <https://doi.org/10.1021/acs.est.3c04959>

Li, H., Zhang, Z., Xiong, T., Tang, K., He, C., Shi, Q., Jiao, N., & Zhang, Y. (2022). Carbon sequestration in the form of recalcitrant dissolved organic carbon in a seaweed (Kelp) farming environment. *Environmental Science and Technology*, *56*(12), 9112–9122. <https://doi.org/10.1021/acs.est.2c01535>

Liu, S., Trevathan-Tackett, S. M., Ewers Lewis, C. J., Huang, X., & Macreadie, P. I. (2020). Macroalgal Blooms Trigger the Breakdown of Seagrass Blue Carbon. *Environmental Science & Technology*, 54(22), 14750–14760. https://doi.org/10.1021/acs.est.0c03720

Liang, J., Liu, J., Zhan, Y., Zhou, S., Xue, C. X., Sun, C., Lin, Y., Luo, C., Wang, X., & Zhang, X. H. (2021). Succession of marine bacteria in response to *Ulva prolifera*-derived dissolved organic matter. *Environment International*, *155*, 106687. <https://doi.org/10.1016/j.envint.2021.106687>

Litchfield, S. G., Schulz, K. G., & Kelaher, B. P. (2020). The influence of plastic pollution and ocean change on detrital decomposition. *Marine Pollution Bulletin*, *158*, 111354. <https://doi.org/10.1016/j.marpolbul.2020.111354>

Lopes, M. L., Martins, P., Ricardo, F., Rodrigues, A. M., & Quintino, V. (2011). *In situ* experimental decomposition studies in estuaries: A comparison of *Phragmites australis* and *Fucus vesiculosus*. *Estuarine, Coastal and Shelf Science*, *92*(4), 573–580. <https://doi.org/10.1016/j.ecss.2011.02.014>

Lozada, M., Diéguez, M. C., García, P. E., & Dionisi, H. M. (2023). Microbial communities associated with kelp detritus in temperate and subantarctic intertidal sediments. *Science of the Total Environment*, *857*(July 2022). <https://doi.org/10.1016/j.scitotenv.2022.159392>

Lucas, M. I., Newell, R. C., & Velimirov, B. (1981). Heterotrophic utilisation of mucilage released during fragmentation of kelp (*Ecklonia maxima* and *Laminaria pallida*). 11. Differential utilisation of dissolved organic components from kelp mucilage. *Marine Ecology Progress Series*, *4*, 43–55.

Luo, H., Dai, X., Yang, Y., & Xie, S. (2022). The evaluation of C, N, P release and contribution to the water environment during *Gracilaria* litters biomass decay. *Estuarine, Coastal and Shelf Science*, *276*, 108052. <https://doi.org/10.1016/j.ecss.2022.108052>

Luo, H., Wang, Q., Zhang, C., Zhang, L., & Yang, Y. (2021). Bioaccumulation and release of heavy metals during growth and decomposition of cultivated *Gracilaria lemaneiformis*. *Marine Pollution Bulletin*, *173*, 113130. <https://doi.org/10.1016/j.marpolbul.2021.113130>

Luo, H., Xie, S., Dai, X., Wang, Q., & Yang, Y. (2022). Biomass decomposition and heavy metal release from seaweed litter, *Gracilaria lemaneiformis*, and secondary pollution evaluation. *Journal of Environmental Management*, *310*, 114729. <https://doi.org/10.1016/j.jenvman.2022.114729>

MacLachlan, A., & McGwynne, L. (1986). Do sandy beaches accumulate nitrogen? *Marine Ecology Progress Series*, *34*, 191–195. <https://doi.org/10.3354/meps034191>

Manikandan, B., Thomas, A. M., Shetye, S. S., Balamurugan, S., Mohandass, C., & Nandakumar, K. (2021). Macroalgal release of dissolved organic carbon in coral reef and its interaction with the bacteria associated with the coral *Porites lutea*. *Environmental Science and Pollution Research*, *28*(47), 66998–67010. <https://doi.org/10.1007/s11356-021-15096-7>

Nedzarek, A., & Rakusa-Suszczewski, S. (2004). Decomposition of macroalgae and the release of nutrient in Admiralty Bay, King George Island, Antarctica. *Polar Bioscience*, *17*, 26–35.

Nitschke, U., Dixneuf, S., Schmid, M., Ruth, A. A., & Stengel, D. B. (2015). Contribution of living and degrading kelp to coastal iodine fluxes. *Marine Biology*, *162*(9), 1727–1738. <https://doi.org/10.1007/s00227-015-2699-4>

Olabarria, C., Lastra, M., & Garrido, J. (2007). Succession of macrofauna on macroalgal wrack of an exposed sandy beach: Effects of patch size and site. *Marine Environmental Research*, *63*(1), 19–40. <https://doi.org/10.1016/j.marenvres.2006.06.001>

Paalme, T., Kukk, H., Kotta, J., & Orav, H. (2002). ‘In vitro’ and ‘in situ’ decomposition of nuisance macroalgae *Cladophora glomerata* and *Pilayella littoralis*. *Hydrobiologia*, *475*, 469–476. <https://doi.org/10.1007/978-94-017-2464-7_36>

Pedersen, M. F., Filbee-Dexter, K., Frisk, N. L., Sárossy, Z., & Wernberg, T. (2021). Carbon sequestration potential increased by incomplete anaerobic decomposition of kelp detritus. *Marine Ecology Progress Series*, *660*, 53–67. <https://doi.org/10.3354/meps13613>

Pedersen, M. F., & Johnsen, K. L. (2017). Nutrient (N and P) dynamics of the invasive macroalga *Gracilaria vermiculophylla*: Nutrient uptake kinetics and nutrient release through decomposition. *Marine Biology*, *164*(8), 1–12. <https://doi.org/10.1007/s00227-017-3197-7>

Pedersen, M. F., Stæhr, P. A., Wernberg, T., & Thomsen, M. S. (2005). Biomass dynamics of exotic *Sargassum muticum* and native *Halidrys siliquosa* in Limfjorden, Denmark—Implications of species replacements on turnover rates. *Aquatic Botany*, *83*(1), 31–47. <https://doi.org/10.1016/j.aquabot.2005.05.004>

Perkins, A. K., Rose, A. L., Grossart, H.-P., Schulz, K. G., Neubauer, D., Tonge, M. P., Rosentreter, J. A., Eyre, B. D., Rojas-Jimenez, K., Deschaseaux, E., & Oakes, J. M. (2023). Fungi increases kelp (*Ecklonia radiata*) remineralisation and dissolved organic carbon, alkalinity, and dimethylsulfoniopropionate (DMSP) production. *Science of The Total Environment*, *905*, 166957. <https://doi.org/10.1016/j.scitotenv.2023.166957>

Perkins, A. K., Santos, I. R., Rose, A. L., Schulz, K. G., Grossart, H.-P., Eyre, B. D., Kelaher, B. P., & Oakes, J. M. (2022). Production of dissolved carbon and alkalinity during macroalgal wrack degradation on beaches: A mesocosm experiment with implications for blue carbon. *Biogeochemistry*, *160*(2), 159–175. <https://doi.org/10.1007/s10533-022-00946-4>

Pessarrodona, A., Foggo, A., & Smale, D. A. (2019). Can ecosystem functioning be maintained despite climate‐driven shifts in species composition? Insights from novel marine forests. *Journal of Ecology*, *107*(1), 91–104. <https://doi.org/10.1111/1365-2745.13053>

Raut, Y., Morando, M., & Capone, D. G. (2018). Diazotrophic macroalgal associations with living and decomposing *Sargassum*. *Frontiers in Microbiology*, *9*. <https://www.frontiersin.org/journals/microbiology/articles/10.3389/fmicb.2018.03127>

Rice, D. (1982). The detritus nitrogen problem: New observations and perspectives from organic geochemistry. *Marine Ecology Progress Series*, *9*, 153–162. <https://doi.org/10.3354/meps009153>

Rice, D. L., & Tenore, K. R. (1981). Dynamics of carbon and nitrogen during the decomposition of detritus derived from estuarine macrophytes. *Estuarine, Coastal and Shelf Science*, *13*(6), 681–690. <https://doi.org/10.1016/S0302-3524(81)80049-7>

Rieper-Kirchner, M. (1989). Microbial degradation of North Sea macroalgae: Field and laboratory studies. *Botanica Marina*, *32*, 241–252. <https://doi.org/10.1515/botm.1989.32.3.241>

Robinson, J. D., Mann, K. H., & Novitsky, J. A. (1982). Conversion of the particulate fraction of seaweed detritus to bacterial biomass. *Limnology and Oceanography*, *27*(6), 1072–1079. <https://doi.org/10.4319/lo.1982.27.6.1072>

Rodil, I. F., Olabarria, C., Lastra, M., & López, J. (2008). Differential effects of native and invasive algal wrack on macrofaunal assemblages inhabiting exposed sandy beaches. *Journal of Experimental Marine Biology and Ecology*, *358*(1), 1–13. <https://doi.org/10.1016/j.jembe.2007.12.030>

Salathe, R., & Riera, P. (2012). The role of *Talitrus saltator* in the decomposition of seaweed wrack on sandy beaches in northern Brittany: An experimental mesocosm approach. *Cahiers de Biologie Marine*, *53*, 517–524.

Salovius, S., & Bonsdorff, E. (2004). Effects of depth, sediment and grazers on the degradation of drifting filamentous algae (*Cladophora glomerata* and *Pilayella littoralis*). *Journal of Experimental Marine Biology and Ecology*, *298*(1), 93–109. <https://doi.org/10.1016/j.jembe.2003.08.006>

Shafique, S., Siddiqui, P. J. A., Aziz, R. A., Burhan, Z.-U.-N., & Mansoor, S. N. (2010). Weight loss and changes in organic, inorganic and chlorophyll contents in three species of seaweeds during decomposition. *Pakistan Journal of Botany*, *42*(4), 2599–2604.

Smale, D. A., Pessarrodona, A., King, N., & Moore, P. J. (2022). Examining the production, export, and immediate fate of kelp detritus on open-coast subtidal reefs in the Northeast Atlantic. *Limnology and Oceanography*, *67*(S2), S36–S49. <https://doi.org/10.1002/lno.11970>

Smith, B. D., & Foreman, R. E. (1984). An assessment of seaweed decomposition within a southern Strait of Georgia seaweed community. *Marine Biology*, *84*(2), 197–205. <https://doi.org/10.1007/BF00393005>

Stuart, V., Lucas, M., & Newell, R. (1981). Heterotrophic utilisation of particulate matter from the kelp *Laminaria pallida*. *Marine Ecology Progress Series*, *4*, 337–348. <https://doi.org/10.3354/meps004337>

Tenore, K. R., Hanson, R. B., McClain, J., Maccubbin, A. E., & Hodson, R. E. (1984). Changes in composition and nutritional value to a benthic deposit feeder of decomposing detritus pools. *Bulletin of Marine Science*, *35*.

Thomson, A., Kristensen, E., Valdemarsen, T., & Quintana, C. (2020). Short-term fate of seagrass and macroalgal detritus in *Arenicola marina* bioturbated sediments. *Marine Ecology Progress Series*, *639*, 21–35. <https://doi.org/10.3354/meps13281>

Twilley, R. R., Ejdung, G., Romare, P., & Kemp, W. M. (1986). A comparative study of decomposition, oxygen consumption and nutrient release for selected aquatic plants occurring in an estuarine environment. *Oikos*, *47*(2), 190–198. <https://doi.org/10.2307/3566045>

Urban-Malinga, B., & Burska, D. (2009). The colonization of macroalgal wrack by the meiofauna in the Arctic intertidal. *Estuarine, Coastal and Shelf Science*, *85*(4), 666–670. <https://doi.org/10.1016/j.ecss.2009.09.033>

Urban-Malinga, B., Gheskiere, T., Degraer, S., Derycke, S., Opalinski, K. W., & Moens, T. (2008). Gradients in biodiversity and macroalgal wrack decomposition rate across a macrotidal, ultradissipative sandy beach. *Marine Biology*, *155*(1), 79–90. <https://doi.org/10.1007/s00227-008-1009-9>

Vichkovitten, T., & Holmer, M. (2004). Contribution of plant carbohydrates to sedimentary carbon mineralization. *Organic Geochemistry*, *35*(9), 1053–1066. <https://doi.org/10.1016/j.orggeochem.2004.04.007>

Wada, S., Aoki, M. N., Mikami, A., Komatsu, T., Tsuchiya, Y., Sato, T., Shinagawa, H., & Hama, T. (2008). Bioavailability of macroalgal dissolved organic matter in seawater. *Marine Ecology Progress Series*, *370*, 33–44. <https://doi.org/10.3354/meps07645>

Watanabe, K., Yoshida, G., Hori, M., Umezawa, Y., Moki, H., & Kuwae, T. (2020). Macroalgal metabolism and lateral carbon flows can create significant carbon sinks. *Biogeosciences*, *17*(9), 2425–2440. <https://doi.org/10.5194/bg-17-2425-2020>

Williams, S. L. (1984). Decomposition of the tropical macroalga *Caulerpa cupressoides* (West) C. Agardh: Field and laboratory studies. *Journal of Experimental Marine Biology and Ecology*, *80*(2), 109–124. <https://doi.org/10.1016/0022-0981(84)90007-8>

Wright, L. S., & Kregting, L. (2023). Genus-specific response of kelp photosynthetic pigments to decomposition. *Marine Biology*, *170*(11), 144. <https://doi.org/10.1007/s00227-023-04289-y>

Wright, L. S., Pessarrodona, A., & Foggo, A. (2022). Climate-driven shifts in kelp forest composition reduce carbon sequestration potential. *Global Change Biology*, *28*(18), 5514–5531. <https://doi.org/10.1111/gcb.16299>

Xie, Y., Su, J., Shao, K., Hu, T., Ming, H., Shi, T., Wang, W., & Fan, J. (2024). Long-term response of the microbial community to the degradation of DOC released from *Undaria pinnatifida*. *Marine Environmental Research*, *194*, 106313. <https://doi.org/10.1016/j.marenvres.2023.106313>

Yamuza-Magdaleno, A., Jiménez-Ramos, R., Casal-Porras, I., Brun, F. G., & Egea, L. G. (2024). Long-term sediment organic carbon remineralization in different seagrass and macroalgae habitats: Implication for blue carbon storage. *Frontiers in Marine Science*, *11*. <https://doi.org/10.3389/fmars.2024.1370768>

Zielinski, K. (1981). Benthic macroalgae of Admiralty Bay (King George Island, South Shetland Islands) and circulation of algal matter between the water and the shore. *Polish Polar Research*, *2*(3–4), 71–94.

Zhang, M., Qin, H., Wang, Z., Li, B., & Ma, Y. (2022). The interaction between DOC released by cultured kelp (*Saccharina japonica*) and the bacterial community reveals the potential for increasing marine carbon sequestration by macroalgae culture. *Frontiers in Marine Science*, *9*, 985548. <https://doi.org/10.3389/fmars.2022.985548>
